# Early defects in mucopolysaccharidosis type IIIC disrupt excitatory synaptic transmission

**DOI:** 10.1172/jci.insight.142073

**Published:** 2021-08-09

**Authors:** Camila Pará, Poulomee Bose, Luigi Bruno, Erika Freemantle, Mahsa Taherzadeh, Xuefang Pan, Chanshuai Han, Peter S. McPherson, Jean-Claude Lacaille, Éric Bonneil, Pierre Thibault, Claire O’Leary, Brian Bigger, Carlos Ramon Morales, Graziella Di Cristo, Alexey V. Pshezhetsky

**Affiliations:** 1 CHU Sainte-Justine Research Center, University of Montréal, Montréal, Québec, Canada.; 2 Department of Anatomy and Cell Biology, McGill University, Montréal, Québec, Canada.; 3 Department of Neurosciences, Faculty of Medicine, University of Montréal, Montréal, Québec, Canada.; 4 Department of Neurology and Neurosurgery, Montréal Neurological Institute, McGill University, Montréal, Québec, Canada.; 5 Proteomic Platform, Institute for Research in Immunology and Cancer, University of Montréal, Montréal, Québec, Canada.; 6 Stem Cell & Neurotherapies, Division of Cell Matrix Biology and Regenerative Medicine, School of Biological Sciences, Faculty of Biology Medicine and Health, University of Manchester, Manchester, United Kingdom.

**Keywords:** Genetics, Neuroscience, Lysosomes, Synapses

## Abstract

The majority of patients affected with lysosomal storage disorders (LSD) exhibit neurological symptoms. For mucopolysaccharidosis type IIIC (MPSIIIC), the major burdens are progressive and severe neuropsychiatric problems and dementia, primarily thought to stem from neurodegeneration. Using the MPSIIIC mouse model, we studied whether clinical manifestations preceding massive neurodegeneration arise from synaptic dysfunction. Reduced levels or abnormal distribution of multiple synaptic proteins were revealed in cultured hippocampal and CA1 pyramidal MPSIIIC neurons. These defects were rescued by virus-mediated gene correction. Dendritic spines were reduced in pyramidal neurons of mouse models of MPSIIIC and other (Tay-Sachs, sialidosis) LSD as early as at P10. MPSIIIC neurons also presented alterations in frequency and amplitude of miniature excitatory and inhibitory postsynaptic currents, sparse synaptic vesicles, reduced postsynaptic densities, disorganized microtubule networks, and partially impaired axonal transport of synaptic proteins. Furthermore, postsynaptic densities were reduced in postmortem cortices of human MPS patients, suggesting that the pathology is a common hallmark for neurological LSD. Together, our results demonstrate that lysosomal storage defects cause early alterations in synaptic structure and abnormalities in neurotransmission originating from impaired synaptic vesicular transport, and they suggest that synaptic defects could be targeted to treat behavioral and cognitive defects in neurological LSD patients.

## Graphical abstract

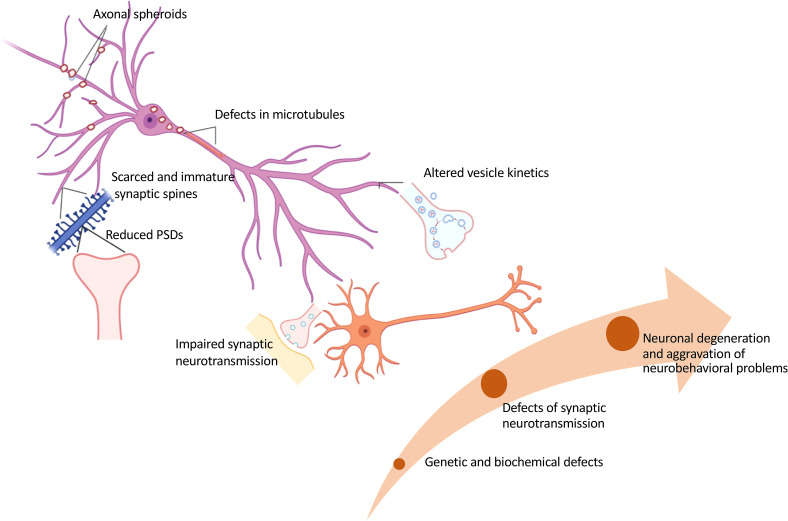

## Introduction

Lysosomal storage diseases (LSD) are progressive, pediatric multisystemic disorders, with the typical cellular landmark of storage bodies, caused by lysosomal accumulation of undigested macromolecules. More than two-thirds of LSD patients present with CNS indications involving cognitive or motor impairment (1). Neurological manifestations are particularly common among mucopolysaccharidoses (MPS), which comprise approximately 30% of LSD cases (2). Progressive and severe neurological decline is the major burden for mucopolysaccharidosis type III, also known as Sanfilippo syndrome, a rare genetic neurogenerative disease manifesting with neuropsychiatric problems, such as hyperactivity, aggressiveness, and autistic features, followed by developmental delay, speech and hearing loss, and childhood dementia (3). Most patients develop gait disorders, become wheelchair-bound during adolescence, and die before adulthood. In some cases, however, survival may exceed the fourth decade of life (3), with progressive dementia and retinitis pigmentosa (4–7). MPSIII is the most prevalent MPS disorder occurring with a combined frequency of 0.28–4.1 per 100,000 live births (2, 8). Four subtypes — A, B, C, and D — of the disease are associated with deficiencies of specific enzymes catalyzing subsequent steps of heparan sulfate (HS) catabolism (4, 5): sulfamidase (MPSIIIA, OMIM 252900) (9), α-N-acetylglucosaminidase (MPSIIIB, OMIM 252920) (10), heparan acetyl CoA α-glucosaminide N-acetyltransferase or HGSNAT (MPSIIIC, OMIM 252930) (11), and N-acetylglucosamine-6-sulfatase (MPSIIID, OMIM 252940) (12).

Our previous studies of the *Hgsnat-Geo* mouse model of MPSIIIC revealed that these animals develop hyperactivity and learning impairment at 5–6 months, followed by urinary retention and death at 10–11 months of age (13). Analysis of pathological changes in the brain demonstrated that primary accumulation of HS in microglial cells and neurons causes impaired autophagy, secondary neuronal storage of G_M2_/G_M3_ gangliosides and misfolded proteins (13), neuroinflammation, and abnormalities in mitochondrial energy metabolism, eventually leading to neuronal death (13). Importantly, neuronal loss in the MPSIIIC mice becomes significant only after 10 months of age, whereas behavioral abnormalities manifest as early as 4 months. Imaging studies in MPSIIIA and MPSIIIB patients have shown a correlation between gray matter deterioration and decrease in development quotient scores (14–16); however, the degree of atrophic change does not always correspond to clinical severity. While some patients with a severe clinical outcome demonstrated moderate MRI alterations (17), others showed extensive neuronal degradation but relatively mild neuropsychiatric signs (18). Together, these observations suggest that neurobehavioral and cognitive manifestations in animal MPSIII models and human patients could be caused by functional pathological changes within neurons.

Synaptic pathology has been previously reported in several animal models of LSD, including feline models of G_M1_ gangliosidosis (19, 20), a mouse model of Niemann-Pick type C disease (21) and the Twitcher mouse, which is natural mouse model of Krabbe disease (22). Recently, reduced excitatory synaptic strength on the somatosensory cortex was demonstrated in a mouse model of MPSIIIA (23). For the same MPSIIIA mouse model, another study reported inhibition of soluble NSF attachment receptor (SNARE) complex assembly and synaptic vesicle recycling, possibly caused by perikaryal accumulation of insoluble α-synuclein and increased proteasomal degradation of cysteine string protein α (CSPα), resulting in low availability of these proteins at the synaptic terminal (24). However, both α-synuclein accumulation and CSPα deficiency manifest in 8-month-old MPSIIIA mice (24), when these animals are at the advanced/terminal stage of the disease concomitant with the massive neuronal death. It is, therefore, implausible that these changes are the underlying cause of the early symptoms of disease, such as hyperactivity and deficits of working memory that appear in this model as early as at 3–4 months, and likely correlate with the presence of defects in synaptic neurotransmission and/or long-term potentiation (25).

In the current study, we demonstrate that synaptic pathology in hippocampal pyramidal neurons of MPSIIIC mice, including deficits in dendritic spines and synaptic vesicles, and abnormalities in neurotransmission, are very early events appearing during the postnatal period, long before the emergence of the first noticeable symptoms. Moreover, our data suggest that defects in glutamatergic neurotransmission originating from deficits in axonal transport are recurring features of multiple neurological LSD and that they should be considered as an essential part of pathophysiology and potential therapeutic targets for patients with a wide range of MPS disorders.

## Results

### Hippocampal MPSIIIC neurons present fewer and less mature dendritic spines.

**Figure 1:**
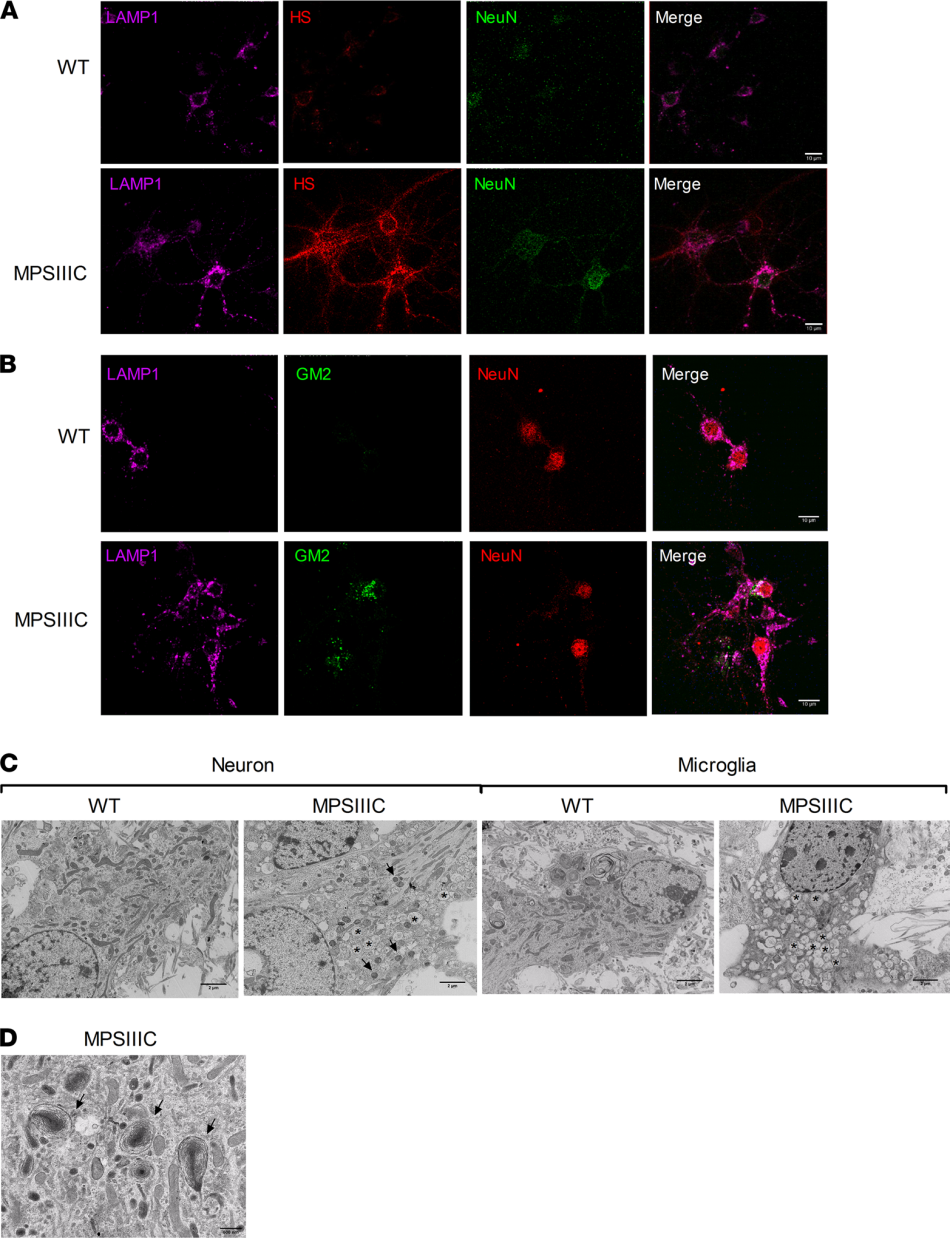
Primary and secondary storage in cultured hippocampal neurons from MPSIIIC mice. (**A**) Cultured primary hippocampal neurons from MPSIIIC mice at DIV21 contain multiple coarse HS^+^/LAMP1^+^ cytoplasmic puncta, consistent with the lysosomal storage of HS. (**B**) Cultured hippocampal MPSIIIC neurons show storage of G_M2_ ganglioside in the granules, only partially colocalizing with LAMP1^+^ vacuoles. (**C** and **D**) The dual pattern of storage is confirmed by electron microscopy, where both electron-dense storage bodies (arrows) containing lipids and misfolded proteins and electron-lucent organelles (asterisks) with glycan storage can be observed in both cultured neurons and microglia derived from the brains of MPSIIIC embryos. Scale bar: 10 μm (**A** and **B**), 2 μm (**C**), and 500 nm (**D**). Data show representative images from 3 experiments, each involving pooled embryos from at least 3 mice per genotype.

Since MPSIIIC mice demonstrate learning impairment (13), our current study focuses on hippocampus. To test for defects in synaptic morphology, we analyzed and quantified dendritic spines of hippocampal pyramidal neurons, which play an important role in learning and memory (26). Importantly, similarly to hippocampal and cortical neurons from the brain of MPSIIIC mice (13), cultured MPSIIIC neurons at day in vitro 21 (DIV21) stored primary (HS) and secondary (G_M2_ ganglioside) metabolites (Figure 1, A and B, respectively), while no storage was detected in WT neurons. HS colocalized with organelles positive for lysosomal-associated membrane protein 1 (LAMP1), while G_M2_ was only partially colocalized with LAMP1, suggesting that this ganglioside also accumulated in nonlysosomal compartments. The accumulation of both metabolites was also observed in lysosomes of NeuN^–^ glial cells present in the neuronal cultures (Figure 1, A and B). Like microglia in vivo, these cells presented more intense HS/LAMP1 staining than neurons (13). Analysis of primary cultures by transmission electron microscopy (TEM) confirmed lysosomal storage in the MPSIIIC neurons and microglia, distinguished from neurons based on their nuclei morphology and electron-dense cytoplasm (27) (Figure 1C). Both types of cells showed electron-dense lamellar bodies compatible with secondary storage of gangliosides and misfolded proteins, as well as electron-lucent vacuoles that can be attributed to the primary storage of HS (Figure 1, C and D).

**Figure 2:**
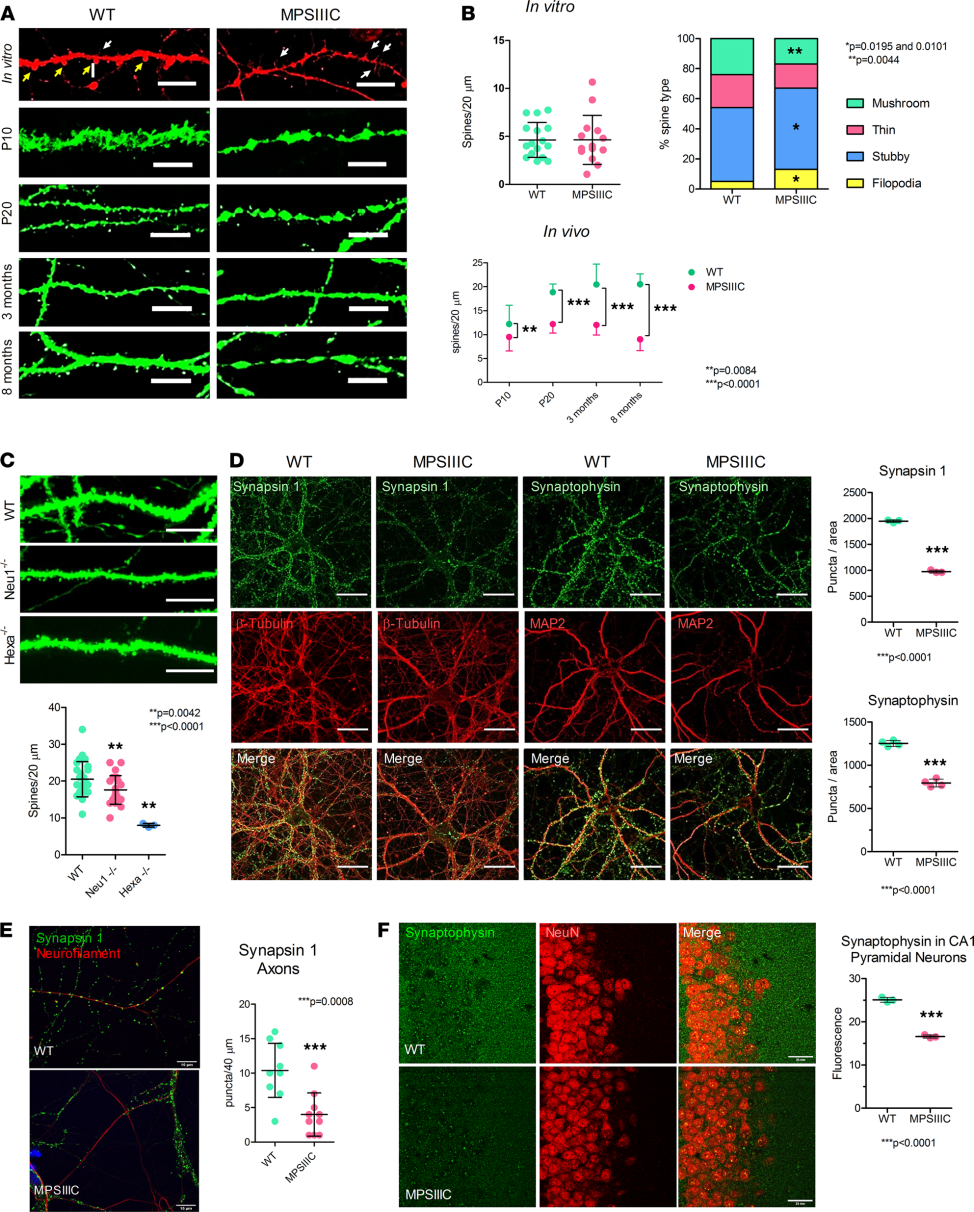
MPSIIIC hippocampal neurons present decreased density of mature dendritic spines, reduced densities of Syn1, and synaptophysin^+^ puncta in the axons. (**A**) Representative images of dendritic spines in cultured neurons at DIV21 and in CA1 pyramidal EGFP-expressing neurons of mice in vitro and at P10, P20, 3 months and 8 months. In cultured neurons, mature (mushroom) spines are marked with yellow arrows, while immature spines (filopodia) are marked with white arrows. The majority of dendritic spines in MPSIIIC are immature. Dendrites in pyramidal MPSIIIC neurons have wider areas resembling spheroids. (**B**) Top: Quantification (left) and distribution of different types of spines (right) in cultured neurons. Bottom: Quantification of dendritic spines in pyramidal neurons. (**C**) Representative images and quantification of dendritic spines in CA1 pyramidal EGFP-expressing neurons of a 3-month-old sialidosis (*Neu1*^–/–^) mouse and a Tay-Sachs (*Hexa*^–/–^) mouse and their respective WT littermates. The quantification of spines was performed in a blinded manner for 20 μm–long dendrite segments starting at 30 μm from the soma. Scale bar: 25 μm for panels and 10 μm for enlargements. (**D**) Representative images of cultured hippocampal neurons at DIV21 from WT and MPSIIIC mice stained for Syn1 and synaptophysin. MPSIIIC neurons have lower density of Syn1^+^ and synaptophysin^+^ puncta. (**E**) Representative images of cultured neurons, stained for Syn1 and an axonal marker, neurofilament medium chain protein. MPSIIIC neurons have lower density of Syn1^+^ puncta per length of the axon. (**F**) Representative images of CA1 pyramidal neurons of 2-month-old MPSIIIC and WT mice stained for synaptophysin and NeuN. MPSIIIC mice have lower density of synaptophysin^+^ puncta. Scale bar: 25 μm (**D** and **F**) and 10 μm (**E**). Graphs show quantification values, mean ± SD obtained for at least 30 cells from 3 mice per each age and genotype. *P* values were calculated using unpaired 2-tailed *t* test (**B** [upper graph] and **D**–**F**); 1-way ANOVA with Tukey post hoc test (**C**), and 2-way ANOVA with Bonferoni post hoc test (**B**, lower graph).

The total spine density (number of spines per 20 μM of the dendrite starting from 30 μm from the soma) was similar for WT and MPSIIIC neurons; however, MPSIIIC neurons displayed an approximately 2-fold higher density of immature filopodia spines and a decreased density of mature “mushroom” spines (Figure 2, A and B, upper panels). Even though the overall length of neurites in MPSIIIC neurons is smaller as compared with the WT (Supplemental Figure 1, A and B; supplemental material available online with this article; https://doi.org/10.1172/jci.insight.142073DS1), Sholl analysis of cultured hippocampal neurons demonstrated that the ramification index and number of intersections are similar between the genotypes (Supplemental Figure 1C), suggesting that immaturity of neurons is not the reason for the synaptic alterations. The dendritic spines were further studied in vivo on pyramidal neurons in the CA1 region of the hippocampus of *Hgsnat-Geo* mice with the *Thy1-EGFP* transgene that expresses the enhanced green fluorescent protein (EGFP) under the control of a modified neuron-specific Thy1 promoter (28). Dendritic spines were quantified at P10, P20, 3 months, and 8 months. As early as at P10, the density of dendritic spines in MPSIIIC mice was reduced by about 22% as compared with WT (Figure 1, A and B, lower panels). The difference between the control and MPSIIIC groups increased further with age, reaching 56% at 8 months (Figure 2B, lower panel).

To test whether reduced density of dendritic spines is a cellular pathology shared among different types of neurological LSD, we quantified spines on pyramidal CA1 neurons in mouse models of Tay-Sachs disease and sialidosis. Human Tay-Sachs patients have mutations in the *HexA* gene encoding for lysosomal β-hexosaminidase A resulting in accumulation of G_M2_ ganglioside (29), while sialidosis patients have mutations in the neuraminidase 1 (*NEU1*) gene, leading to lysosomal storage of sialylated glycoproteins (30, 31). Previously described *Hexa*-KO and *Neu1*-KO mice (13, 32–34) were crossed with the *Thy1-EGFP* strain, and GFP^+^ pyramidal neurons from the CA1 region of the hippocampus were studied by confocal microscopy. Our data reveal that neurons from 3-month-old sialidosis and Tay-Sachs mice also demonstrate reduction of dendritic spine density (Figure 2C). These cells do not accumulate HS, suggesting that spines are not reduced exclusively due to storage of this metabolite.

### Synaptic vesicles are reduced in the terminals of MPSIIIC hippocampal neurons.

To test whether the levels of synaptic vesicles are changed in cultured MPSIIIC neurons, we studied the cellular distribution and density of synapsin 1 (Syn1) and synaptophysin by immunocytochemistry. In neurons from MPSIIIC mice at DIV21, the densities of puncta positive for both proteins were significantly reduced (Figure 2D). The density of Syn1^+^ puncta was reduced by 50% and the density of synaptophysin^+^ puncta was reduced by 37%. We further measured the density of Syn1^+^ puncta specifically associated with the axonal marker, neurofilament medium chain (NF-M), and found it to be significantly reduced (Figure 2E). The density of synaptophysin puncta was also reduced in CA1 pyramidal neurons of 3-month-old MPSIIIC mice (Figure 2F).

**Figure 3:**
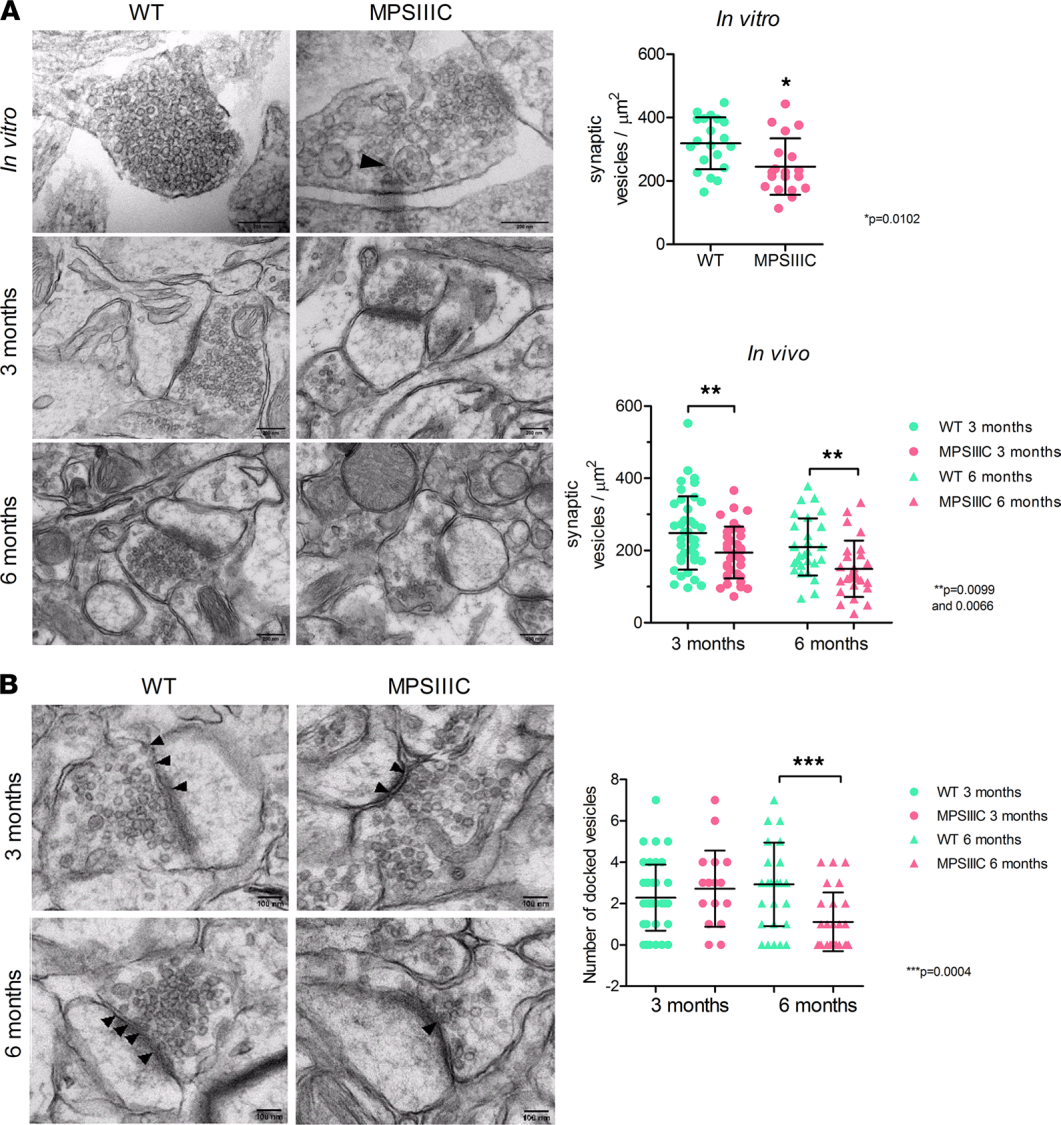
MPSIIIC hippocampal neurons present scarcity of synaptic vesicles in the synaptic terminals. (**A**) Representative TEM images and quantification of synaptic vesicles in terminals of cultured hippocampal neurons at DIV21 and of pyramidal neurons from the CA1 hippocampus region of 3- and 6-month-old mice. An autophagosome in the synaptic terminal is marked with an arrowhead. Scale bar: 200 nm. (**B**) Representative TEM images and quantification of docking synaptic vesicles (arrowheads) in terminals of CA1 neurons from 3- and 6-month-old mice. Graphs show quantification values, mean ± SD obtained for at least 30 cells from 3 mice per each age and genotype. *P* values were calculated using unpaired 2-tailed *t* test.

The density of synaptic vesicles at the synaptic terminals of hippocampal neurons in culture and in pyramidal CA1 neurons from 3- and 6-month-old mice was further analyzed by TEM. In MPSIIIC cultured neurons, the density of synaptic vesicles (total number of synaptic vesicles in the axonal terminal divided by the area of the terminal in μm^2^) was decreased by 23% (Figure 3A). In vivo, 3-month-old MPSIIIC mice also displayed a decrease of 22% in synaptic vesicles as compared with WT. This reduction was even more prominent by the age of 6 months (Figure 3A), when the difference between MPSIIIC and the WT reached 30%. At this time point, we also observed a significant reduction in the number of synaptic vesicles docking at the presynaptic terminal membrane (Figure 3B). Additionally, 10%–20% of terminals in the MPSIIIC neurons in vivo and in vitro contained multivesicular vacuoles with a double limiting membrane (Figure 3A, arrowhead) resembling the autophagosomes found in the neurons of patients with adult neurodegenerative disorders (35). Overall, our TEM results directly confirm the reduction of synaptic vesicles in vivo and in vitro predicted by decrease in Syn1^+^ and synaptophysin^+^ puncta.

### MPSIIIC neurons show alterations in distribution of excitatory synaptic markers.

**Figure 4:**
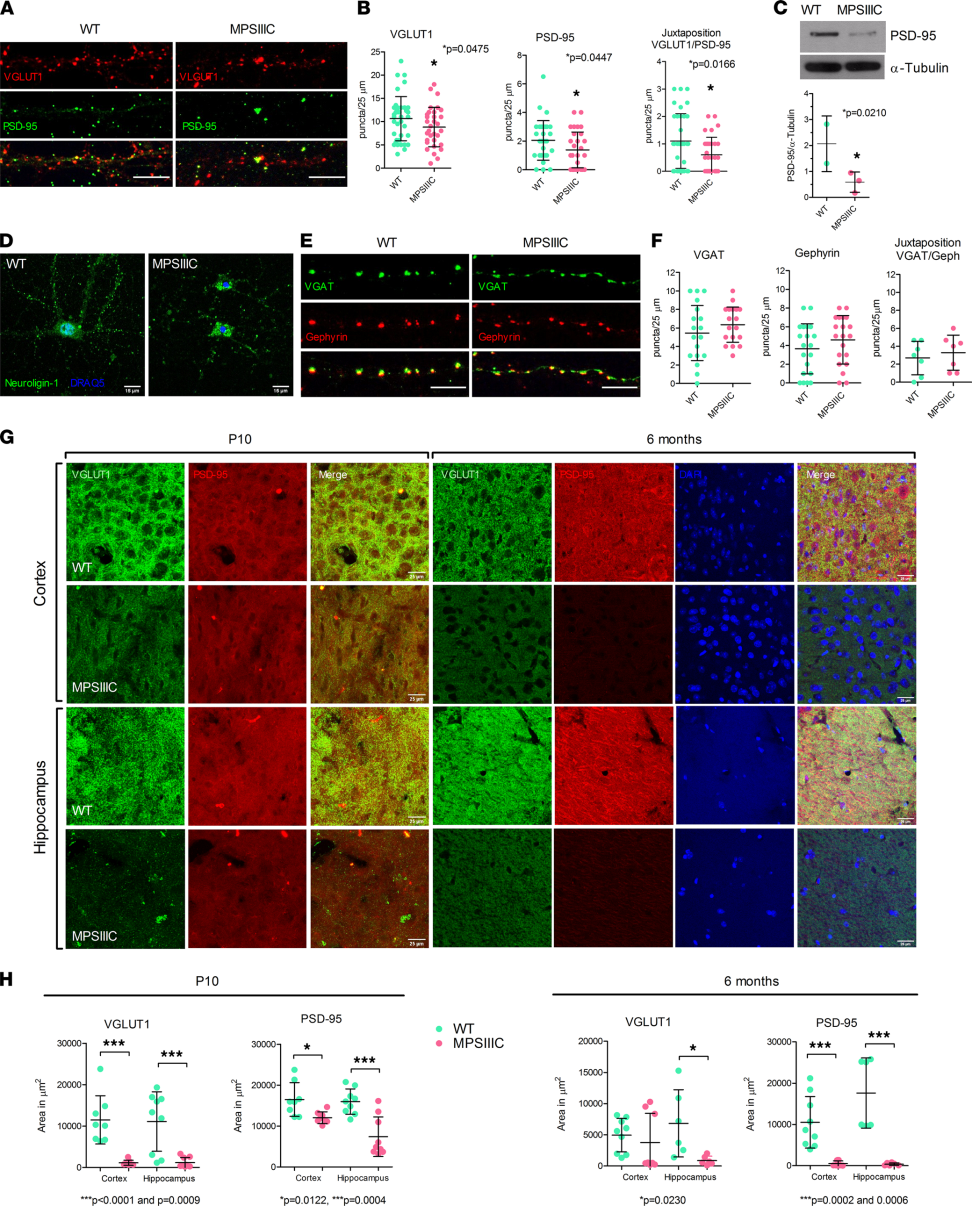
MPSIIIC hippocampal and cortical neurons present alterations in the distribution of protein markers of the excitatory synapse. (**A**) Representative images of hippocampal cultured neuron at DIV21 stained for VGLUT1 and PSD-95. Scale bars: 10 μm. (**B**) MPSIIIIC cells show significantly lower densities of PSD-95^+^ puncta and PSD-95^+^ puncta in juxtaposition with VGLUT1^+^ puncta. (**C**) Immunoblotting confirms the reduction of PSD-95 in cultured hippocampal MPSIIIC neurons. (**D**) Representative images of cultured hippocampal neurons stained for Nlgn1 and nuclear marker DRAQ5. In MPSIIIC neurons, Nlgn1 shows perinuclear accumulation instead of fine puncta observed in the neurites of WT cells. Scale bars: 15 μm. (**E**) Representative images of hippocampal cultured neuron at DIV21 stained for VGAT and gephyrin. Juxtaposition between the VGAT^+^ and gephyrin^+^ puncta indicates functional synapses. Scale bars: 10 μm. (**F**) Quantification of VGAT^+^ puncta, gephyrin^+^ puncta, and their juxtaposition. **A**, **C**, **D**, and **E** show representative results of 3 experiments, each involving pooled embryos from at least 3 mice per genotype. The quantification of puncta in **B** and **F** was performed within 25 μm segments of dendrites at 30 μm from the soma in a double-blinded manner, using cultures from 3 independent experiments with a total of 10 cells being analyzed for each experiment. All graphs show individual data, mean ± SD. *P* values were calculated by 2-tailed *t* test. (**G**) Representative confocal images of somatosensory cortex (layers 2/3) and CA1 hippocampal regions of WT and MPSIIIC mice at P10 and 6 months stained for the markers of excitatory synapse, PSD-95 and VGLUT1. Scale bars: 25 μm. (**H**) Density of VGLUT1^+^ and PSD-95^+^ puncta were quantified using ImageJ software. The quantification of puncta was performed in a blinded manner using 3 mice per age per genotype. Three adjacent panels were analyzed in each mouse. Graph shows individual values, mean ± SD. *P* values were calculated by 2-tailed *t* test.

To test whether both excitatory and inhibitory synapses are affected in cultured hippocampal MPSIIIC neurons, we studied by immunocytochemistry the distribution and density of pre- and postsynaptic markers associated with either glutamate-mediated excitatory or γ-aminobutyric acid–mediated (GABA-mediated) inhibitory neurotransmission. Puncta were quantified in 25 μm–long dendrite segments, 40 μm away from the soma. We counted separately isolated pre- and postsynaptic puncta and when they were in juxtaposition, indicating the presence of a functional synapse. The analysis of the excitatory markers VGLUT1 (the presynaptic transporter of glutamate in synaptic vesicles) and postsynaptic density protein 95 (PSD-95, a scaffold protein present on the postsynaptic densities, which interacts with NMDA receptors) (36) demonstrated a significant difference between MPSIIIC and WT neurons (Figure 4A). Quantification of puncta revealed a decreased density of PSD-95 in MSPS IIIC neurons and a reduced number of PSD-95^+^ puncta in juxtaposition with VGLUT1^+^ puncta, suggesting the existence of fewer functional excitatory synapses (Figure 4B). The reduction of PSD-95 in MPSIIIC cultured neurons was also confirmed by Western blot (Figure 4C). Additionally, altered cellular localization was also detected for Neuroligin-1 (Nlgn1), another key synaptic protein that binds to PSD-95 and NMDA-R1 receptor (37) and mediates cell-cell interaction in the synapses by binding to neurexins (38). In MPSIIIC cultured hippocampal neurons, the majority of Nlgn1 accumulated in perinuclear structures in the cell body instead of being localized in the fine puncta in the dendrites, as observed in WT neurons (Figure 4D). Conversely, both density and juxtaposition of puncta for the markers of the inhibitory synapse, VGAT (a transporter involved in the uptake of GABA into synaptic vesicles) and gephyrin (a protein that anchors GABA-A receptors) were similar for MPSIIIC and the WT neurons (Figure 4, E and F). We further analyzed whether PSD-95^+^ puncta and their juxtaposition with VGLUT1^+^ puncta was also reduced in vivo in the brains of MPSIIIC mice. Brain slices from WT and MPSIIIC animals at P10, 3 months (not shown), and 6 months were stained with the corresponding antibodies, and the CA1 area of the hippocampus, as well as layers 2/3 of somatosensoty cortex, analyzed by confocal microscopy (Figure 4G). We found that densities of PSD-95^+^ puncta and VGLUT1^+^ puncta were significantly reduced in both brain areas at all ages (Figure 4H).

**Figure 5:**
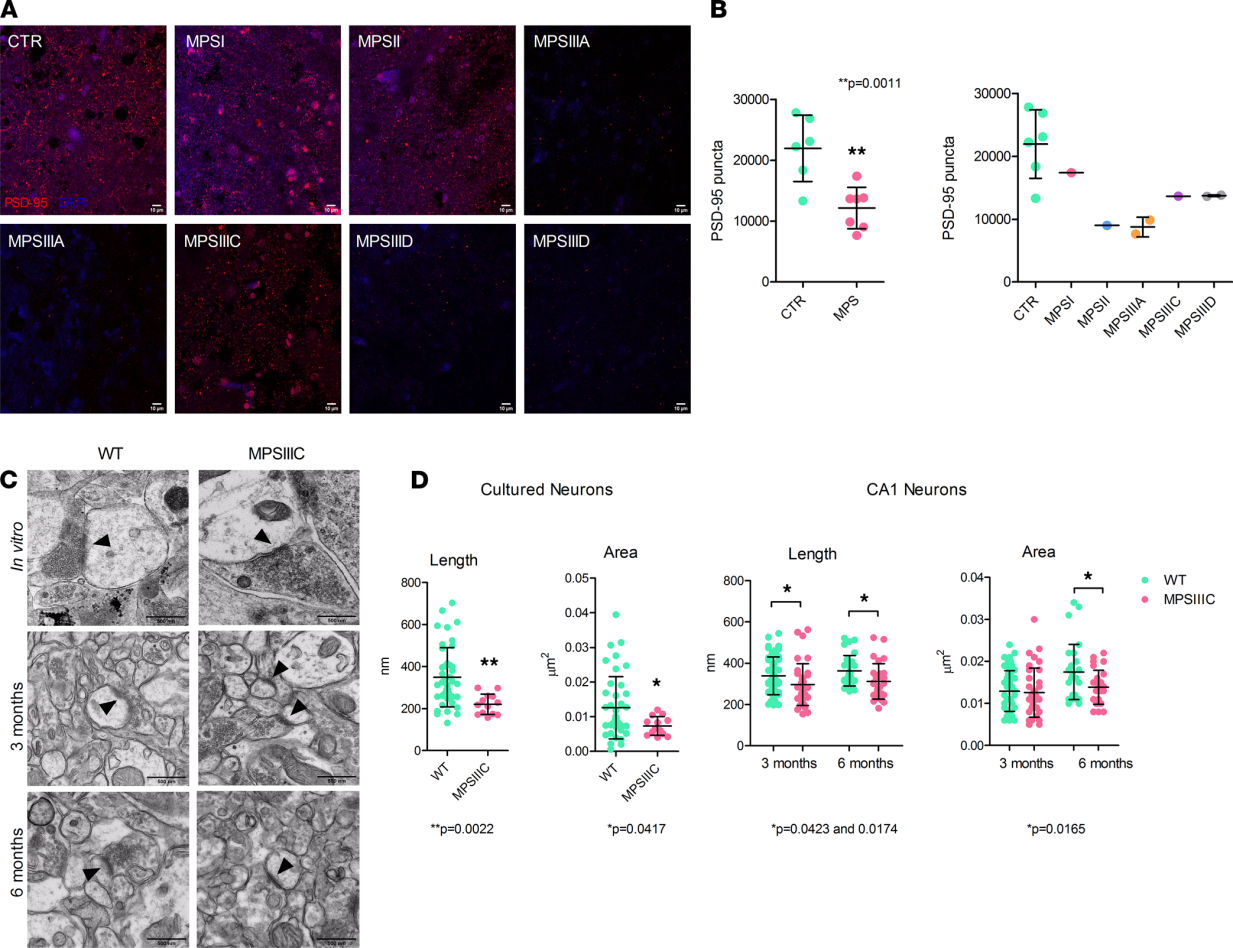
Cortical neurons of neurological MPS patients and hippocampal neurons of MPSIIIC mice present reduction of postsynaptic densities. (**A**) Representative confocal images of postmortem human cortices from controls and MPS patients stained with antibodies against PSD-95. (**B**) Density of PSD-95^+^ puncta in human cortices. Scale bar: 10 μm. (**C**) Representative TEM images of synapses in cultured hippocampal neurons at DIV21 and in pyramidal neurons from the CA1 region of the hippocampus of 3- and 6-month-old mice. PSDs are marked with arrowheads. Scale bar: 250 nm. (**D**) Quantification of length (nm) and area (μm^2^) of PSDs in hippocampal cultured neurons and in pyramidal neurons from the CA1 region of the hippocampus. Data show individual values, mean ± SD of 3 different neuronal cultures or 3 mice per genotype with at least 10 images analyzed for each experiment. Only asymmetric (excitatory) PSDs were considered for analysis. *P* values were calculated by 2-tailed *t* test.

To test if the levels of excitatory PSD are also reduced in human MPS patients, we analyzed PFA-fixed somatosensory cortex postmortem tissues collected at autopsy and donated to the NIH NeuroBioBank. Samples of 8 MPS patients (1 MPSI, 1 MPSII, 2 MPSIIIA, 1 MPSIIIC, and 2 MPSIIID) and 7 non-MPS controls, matched for age and sex, were analyzed (project 1071, MPS Synapse). The age and the cause of death, sex, race, and available clinical and neuropathological information for the patients and controls are shown in Supplemental Table 1. All MPS patients had complications from their primary disease and died prematurely (before 25 years) except for the MPSII patient 902, who died at 42 years from MPSII-related pneumonia. None of the patients had received enzyme replacement therapy or hematopoietic stem cell transplantation. This analysis confirmed that densities of PSD-95^+^ puncta were significantly reduced in human cortices from MPS patients (Figure 5, A and B), suggesting that the reduction of PSD-95 may be a synaptic hallmark common to most neurological MPS. Unfortunately, we could not achieve specific Syn1 staining of human preserved tissues, and no human hippocampi were available for analysis to reproduce results in the mouse tissues.

The length and area of PSD of asymmetric (excitatory) and symmetric (inhibitory) synapses of cultured hippocampal neurons and pyramidal CA1 neurons from 3- and 6-month-old mice were further studied by electron microscopy (EM) (Figure 5C). For each detected PSD, we measured both length (the linear dimension of the PSD along the axonal terminal) and the total area. The length of excitatory PSD in cultured MPSIIIC hippocampal neurons was about 37% less than in the WT cells (Figure 5, C and D). In vivo, excitatory PSD length was reduced by 12.5% in 3-month-old mice and by 14% in 6-month-old mice. The area of excitatory PSD was also smaller by 42% in cultured MPSIIIC neurons as compared with WT cels, whereas in vivo, it was reduced in MPSIIIC mice by 21% at 6 months but not at 3 months (Figure 5, C and D). In contrast, PSD length in symmetrical inhibitory synapses was similar for MPSIIIC and WT neurons in vivo and in vitro (Supplemental Figure 2), consistent with immunolabeling results obtained for GABAergic markers (Figure 4, E and F).

Interestingly, TEM analysis also demonstrated that synapse localization in cultured MPSIIIC neurons was significantly altered as compared with WT neurons. While in the WT neurons, the majority (~80%) of synapses was located on dendritic spines (axospinous synapses), MPSIIIC cells showed a shift toward axodendritic synapses (Supplemental Figure 3, A and B, left panel). On the other hand, we did not detect any difference in synaptic localization on dendrites of CA1 pyramidal neurons between 6-month-old WT and MPSIIIC mice (Supplemental Figure 3B, right panel).

### MPSIIIC neurons show alterations in neurotransmission.

**Figure 6:**
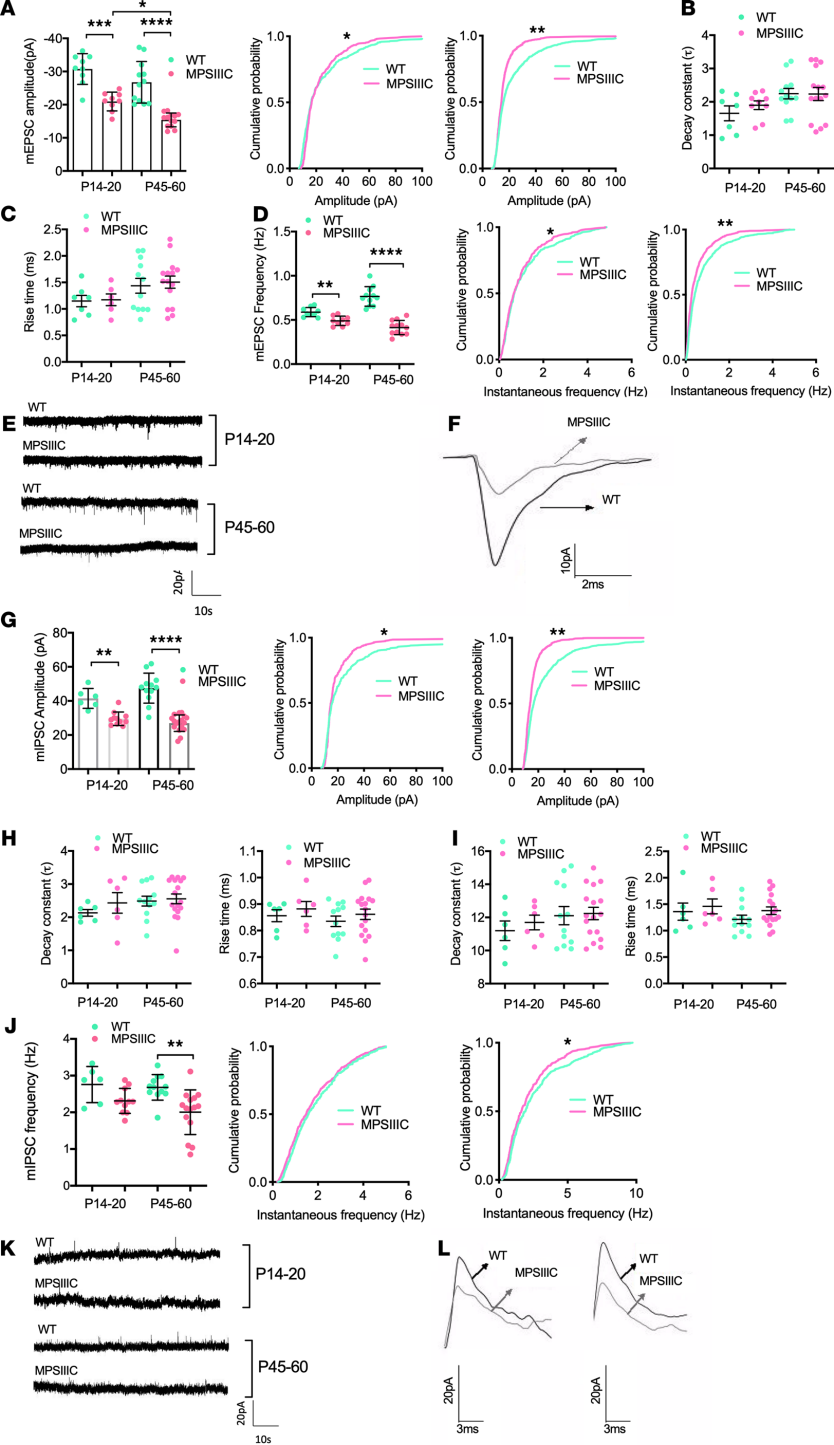
Alteration of miniature excitatory and inhibitory postsynaptic currents in MPSIIIC neurons. (**A**) Distribution of mEPSC amplitudes showing significant decrease in the mEPSC amplitude in MPSIIIC mice at P14–P20 and P45–P60. (**B** and **C**) No significant difference in the decay constant (**B**) or rise time (**C**) of mEPSC events was detected at P14–P20 or P45–P60. (**D**) Distribution of mEPSC instantaneous frequencies showing significant decrease in the mEPSC frequency in MPSIIIC mice as compared with WT in P14–P20 and P45–P60 hippocampal slices. (**E** and **F**) Representative traces of mEPSCs at P14–P20 and P45–P60 (**E**) and overlay of representative individual events from MPSIIIC and WT mice at P45–P60 (**F**). (**G**) Distribution of mIPSC amplitudes showing significant decrease in the mIPSC amplitude in MPSIIIC as compared with WT in P14–P20 and P45–P60 hippocampal slices. (**H**) No significant differences in the fast decay constant or the fast rise time at both ages. (**I**) No significant differences in the slow decay constant or the slow rise time of mIPSCs. (**J**) Distribution of mIPSC instantaneous frequencies showing significant decrease in the mIPSCs frequency in MPSIIIC mice as compared with WT in P45–P60 hippocampal slices. (**K** and **L**) Representative traces of mIPSCs at P14–P20 and P45–P60 (**K**) and overlay of representative individual mIPSC events with fast kinetics (left panel) and slow kinetics (right panel) for MPSIIIC and WT mice at P45–P60 (**L**). Statistical analyses for Gaussian-distributed events were performed using 1-way ANOVA with Tukey’s post test. Non-Gaussian–distributed events were analyzed by Kruskal-Wallis test, followed by Dunn’s test. * *P* < 0.05, ** *P* < 0.01, *** *P* < 0.001, and **** *P* < 0.0001. Kolmogorov-Smirnov test was performed for not normally distributed events.

To test if alterations in the distribution of synaptic markers in MPSIIIC mice translate into changes in synaptic transmission, we conducted electrophysiological recordings of miniature excitatory postsynaptic currents (mEPSCs) and miniature inhibitory postsynaptic currents (mIPSCs) from CA1 neurons in acute hippocampal slices of MPSIIIC and WT mice collected at P14–P20 and P45–P60. Both mEPSC amplitude and frequency were significantly reduced in MPSIIIC mice as compared with WT mice (Figure 6, A–F). At P14–P20, the mEPSC amplitude was reduced by 32%, and at P45–P60, it was reduced by 43%. Moreover, a statistically significant reduction in the mEPSC amplitudes was detected in MPSIIIC mice at P14–P20 as compared with P45–P60, indicating that a glutamatergic deficit worsened with age. A significantly decreased mEPSC frequency was also detected at both developmental timepoints (18% reduction at P14–P20 and a 49% reduction at P45–P60). No significant difference in mEPSC kinetics between the 2 animal groups was observed (Figure 6, B and C). These observations were consistent with reduced VGLUT1 expression and suggested the possibility of a global glutamatergic deficit. Both mIPSC amplitude and frequency were also reduced in MPSIIIC mice as compared with WT (Figure 6, G–L). While at P14–P20, the mIPSC amplitude was reduced by 28.8%, at P45–P60, it was reduced by 43.2%. The mIPSC frequency was also reduced by 23% at P45–P60 but not at P14–P20. Upon assessing the kinetics of the mIPSCs, we found 2 subpopulations of events with fast and slow decay constant and rise times (Figure 6, H and I). Previous studies suggest that different interneuron populations mediate cell type–specific kinetic classes of IPSCs (39, 40). However, for both slow and fast mIPSCs, the kinetics of the events was similar for MPSIIIC and WT animals (Figure 6, H and I).

At the same time, we found no significant differences in resting potential, input resistance, action potential threshold, half width of action potential, or firing frequency between hippocampal CA1 neurons from MPSIIIC and WT mice (Supplemental Table 2). This suggests that the intrinsic excitability of CA1 neurons was not altered in MPSIIIC animals.

Miniature synaptic events were also recorded in cultured hippocampal neurons from MPSIIIC and WT mice (DIV19–DIV22). We found that cultured MPSIIIC neurons (Supplemental Figure 4, A–C) displayed a significant increase in both the frequency and amplitude of mEPSCs. While the mEPSc amplitude was increased by 21.5%, the mEPSC frequency was increased by 43.75% as compared with WT controls. The cumulative probability distribution (Supplemental Figure 4, B and C) also showed significance for mEPSCs with increased amplitude and a decreased interevent interval. In contrast, the mean frequency and amplitude of the inhibitory events in cultured MPSIIIC hippocampal neurons (Supplemental Figure 4, D–F) were not significantly different from those in the WT neurons. The cumulative probability distribution (Supplemental Figure 4B), however, showed significance for mIPSCs with increased amplitude and a decreased interevent interval of mIPSCs. Thus, miniature excitatory synaptic transmission was consistently increased, but inhibitory transmission was not.

### Rescue of PSD-95 deficiency in MPSIIIC neurons.

To establish a causative relationship between the deficits in the expression of protein markers for excitatory synapses and HGSNAT deficiency, we attempted to rescue this phenotype by a virus overexpressing the WT human HGSNAT enzyme. Human codon-optimized HGSNAT cDNA fused with that of GFP was cloned into a third-generation lentiviral vector (LV) under the control of a CMV promoter. The LV was tested in HEK-293T cells transduced with a multiplicity of infection (MOI) of 10. The transduced cells expressed HGSNAT-GFP fusion protein correctly targeted to the lysosomes and had 120-fold increased HGSNAT activity as compared with nontransduced cells (Supplemental Figure 5).

**Figure 7:**
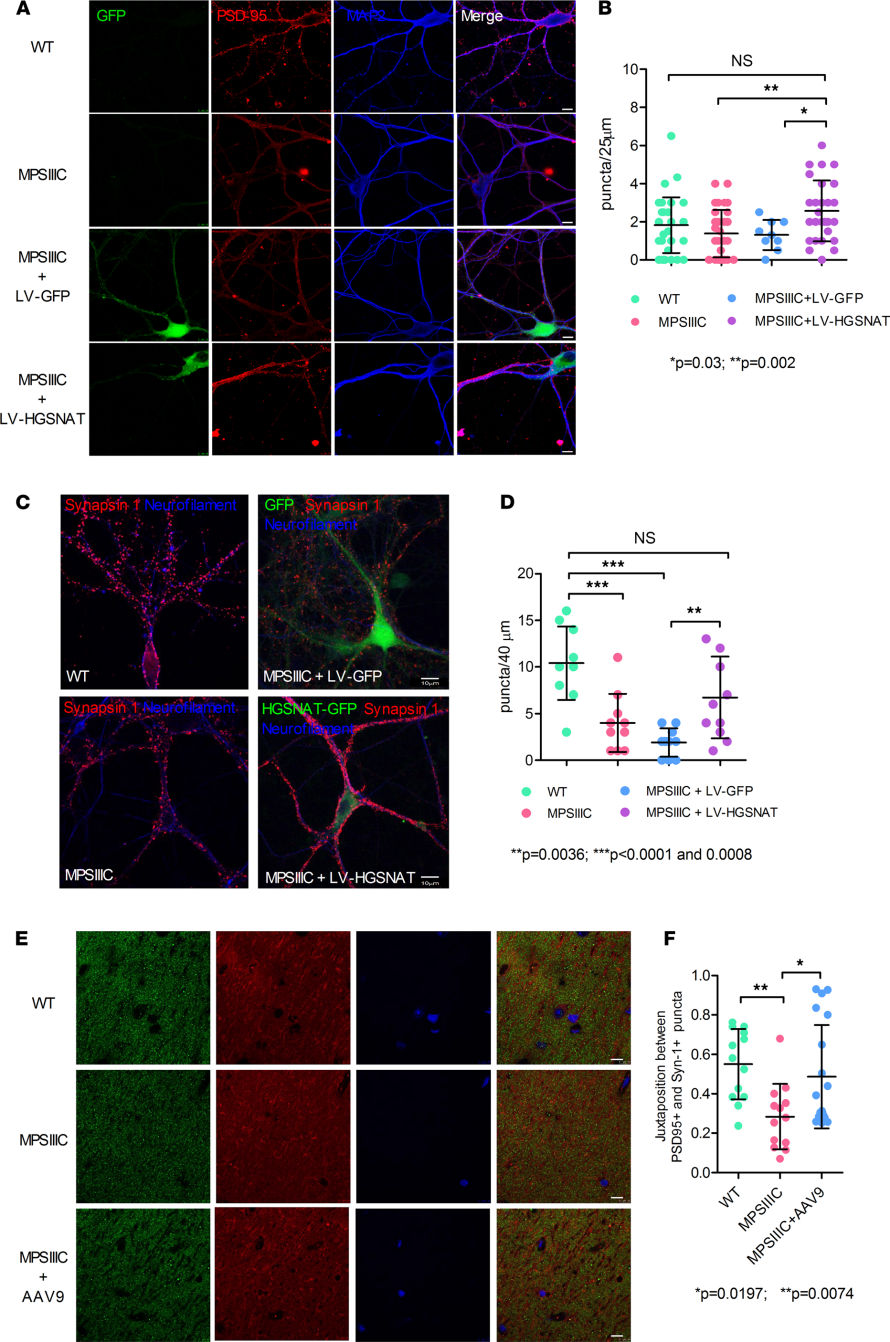
Deficit of PSD-95 and Syn1 in MPSIIIC neurons is rescued in vitro and in vivo by transduction with viral vectors encoding for WT human HGSNAT. ( **A**) Representative images of cultured hippocampal WT and MPSIIIC neurons, and MPSIIIC neurons, transduced with either LV-GFP or LV-HGSNAT-GFP, stained with anti–PSD-95 and anti-MAP2 antibodies. (**B**) Quantification of PSD-95 puncta in WT, and MPSIIIC cells — as well as in MPSIIIC cells — transduced with LV-GFP or LV-HGSNAT-GFP. (**C**) Representative images of cultured WT and MPSIIIC neurons, and MPSIIIC neurons, transduced with either LV-GFP or LV-HGSNAT-GFP, stained with anti-Syn1 and anti-neurofilament antibodies. (**D**) Quantification of Syn1 puncta in the axons of cultured neurons. Graphs in **B** and **D** show individual data, mean ± SD for at least 9 cells from 3 independent cultures, each with cells pooled from 3 or more embryos per genotype. *P* values were calculated using 1-way ANOVA with Bonferroni post hoc test. (**E**) Representative images of CA1 region of the hippocampus from untreated WT and MPSIIIC mice or MPSIIIC mice treated with AAV9-HGSNAT, stained with anti-Syn1 and anti–PSD-95 antibodies. Scale bar: 10 μm. (**F**) Quantification of Syn1^+^ and PSD-95^+^ puncta in juxtaposition. Graphs show data from 4 or 5 different mice per condition, with 3 images analyzed per animal. *P* value was calculated by 1-way ANOVA with Bonferroni analysis for multiple comparisons.

We then transduced MPSIIIC primary hippocampal neurons at DIV3 with either LV-GFP or LV-HGSNAT-GFP, kept them in culture until DIV21, then fixed and analyzed by confocal fluorescence microscopy to measure PSD-95 density in dendrites (Figure 7, A and B) and Syn1 in axons (Figure 7, C and D). Quantification of images (Figure 7B) reveal that the density of PSD-95 puncta in MPSIIIC hippocampal neurons transduced with LV-HGSNAT-GFP, but not with LV-GFP, was increased to a level similar to that in WT neurons. The density of Syn1^+^ puncta associated with NF-stained axons, and calculated per 40 μm of the axon significantly reduced in MPSIIIC neurons as compared with the WT cells, was significantly increased in the MPSIIIC cells transduced with LV-HGSNAT-GFP as compared with the LV-GFP–transduced cells (Figure 7D).

Previously, we demonstrated a rescue of the behavioral defects, primary and secondary lysosomal storage, and neuroinflammation in female MPSIIIC mice receiving intracranial injections of AAV vectors (AAV9 and AAV2 true type [TT]) encoding for human untagged HGSNAT (41). We now analyzed cryopreserved brains from this study to investigate if AAV vectors also rescued synaptic deficits. At the age of 4 weeks, the mice were injected in both striatum with 2.6 × 10^9^ AAV9 vg/hemisphere in saline or with saline alone, and were than sacrificed at 6 months after treatment with their brains fixed and cryopreserved. The brains were compared with the control of those from MPSIIIC and WT female mice that received sham injections of saline. The brain slices of AVV9-HGSNAT–treated and sham-treated mice were stained with antibodies against PSD-95 and Syn1, and the density of PSD-95^+^ and Syn1^+^ puncta in juxtaposition was assessed in the hippocampal CA1 area (Figure 7E). HGSNAT expression significantly increased the density of PSD-95^+^ puncta in juxtaposition with Syn1^+^ puncta (Figure 7F). There was also a trend for an increase of Syn1^+^ puncta density, but the results were not statistically significant, perhaps due to a high difference between the individual animals in the groups (data not shown). Thus, by restoring the primary HGSNAT deficiency, we could rescue PSD-95 defects both in vivo and in vitro.

### Analysis of synaptosomes from MPSIIIC mice reveals a dramatic decrease in synaptic vesicle associated and mitochondrial proteins.

**Figure 8:**
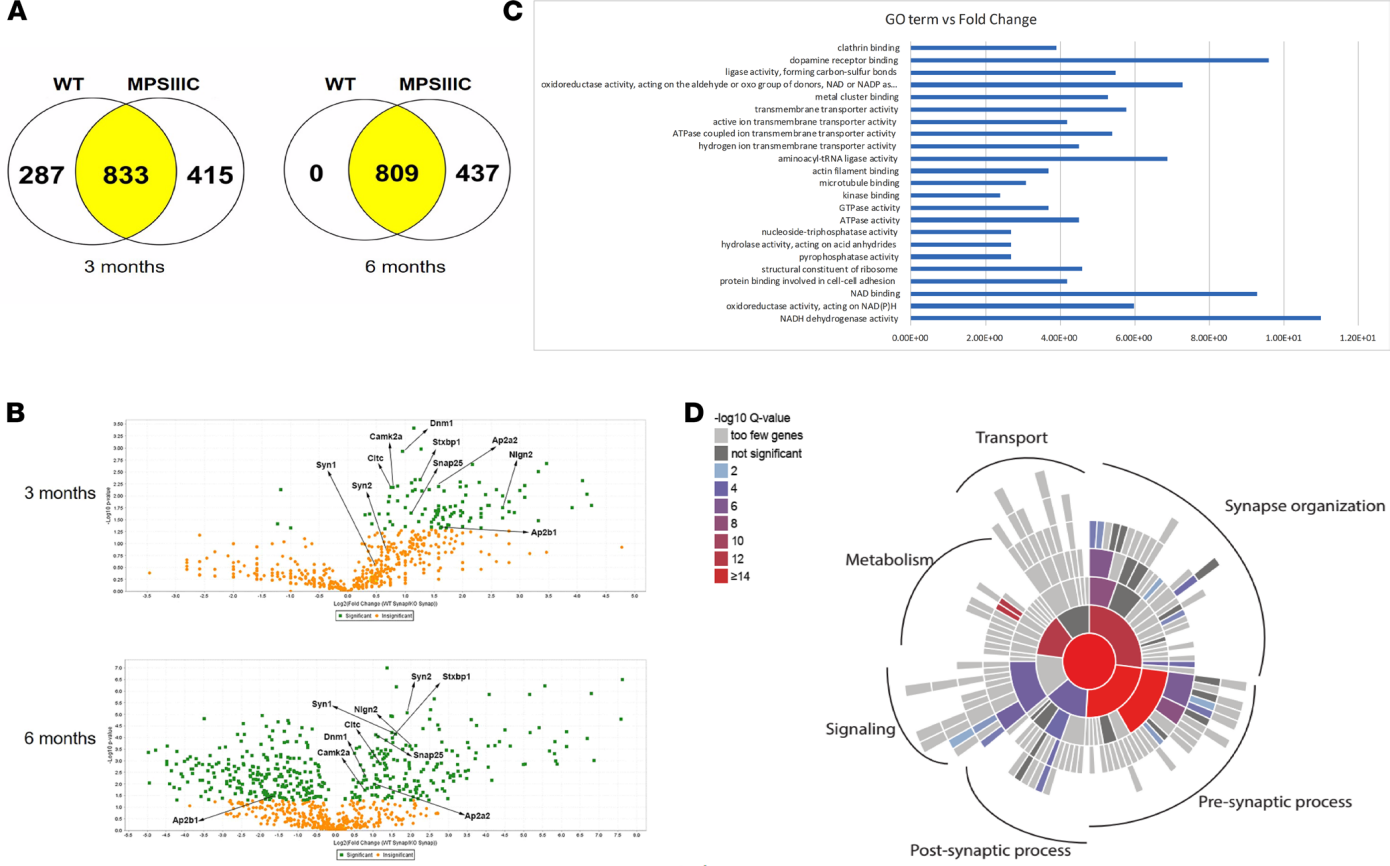
Semiquantitative LC-MS/MS analysis of proteins present in synaptosomes from brains of 3- and 6-month-old mice reveals deficiencies of synaptic, mitochondrial, and trafficking vesicle–associated proteins in MPSIIIC mice. (**A**) Total number of proteins identified by LC-MS/MS in synaptosomes extracted from the brains of WT and MPSIIIC mice at 3 and 6 months of age. (**B**) Volcano plots of the proteins identified in synaptosomes showing proteins that are statistically different between WT and MPSIIIC. (**C**) Gene ontology (GO) terms versus fold-change of the proteins reduced in MPSIIIC synaptosomes. The data represent values where the Benjamini-Hochberg-corrected *P* value is below the highest Benjamini-Hochberg-corrected *P* value for the GO terms. (**D**) Numbers of *q* values per ontology term showing significantly enriched biological processes. The *q* values represent *P* values adjusted for FDR: *q* value = *P* value × (total number of hypotheses tested)/(rank of the *P* value).

**Figure 9:**
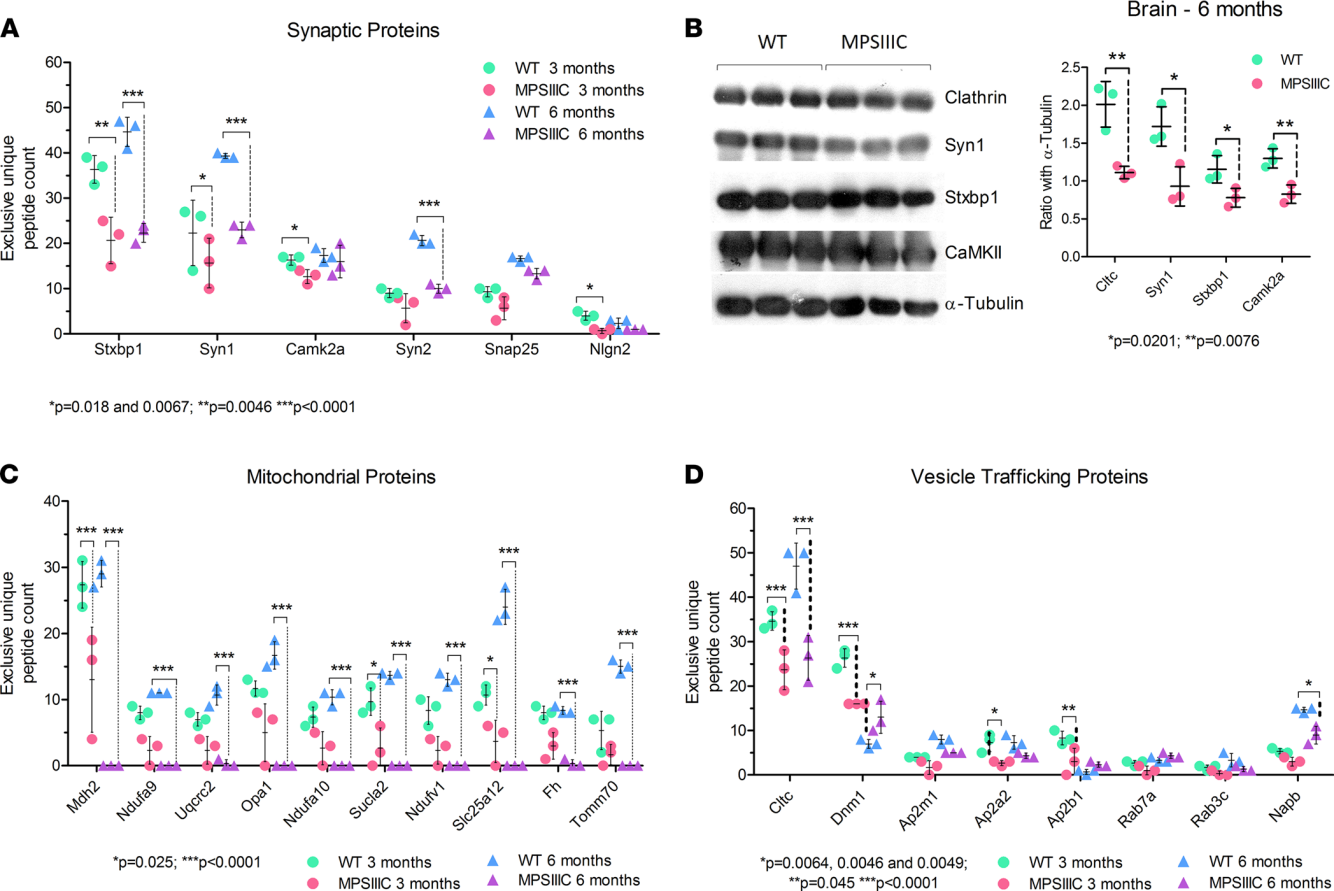
Deficiencies of synaptic, mitochondrial, and trafficking vesicle–associated proteins in brains of 3- and 6-month-old MPSIIIC mice. (**A**) Exclusive unique peptide counts of synaptic proteins. (**B**) Western blots of total protein extracts from brains of 6-month-old mice and their respective quantifications confirming changes in protein abundance identified by proteomic analysis. (**C**) Exclusive unique peptide counts of mitochondrial proteins. (**D**) Exclusive unique peptide counts of proteins associated with intracellular vesicle trafficking and endocytosis. Proteomic analyses and Western blots were performed using synaptosomes extracted from 3 different animals per age per genotype. *P* values for the exclusive unique peptide counts areas on the peptide chromatograms were calculated using 2-way ANOVA with Bonferroni post hoc test. *P* values for **B** were calculated using 2-tailed *t* test.

To gain insights into the molecular mechanisms underlying synaptic deficits in MPSIIIC neurons, we performed proteomic analyses of mouse brain synaptosomes (42). Synaptosomes were isolated from MPSIIIC and WT mice at 3 and 6 months of age by differential centrifugation using a commercially available kit, and their protein content was analyzed by quantitative proteomics using label-free liquid chromatography–tandem mass spectrometry (LC-MS/MS). Based on immunoblot analysis, the synaptosome preparation was > 3-fold enriched in synaptic proteins as compared with the whole brain homogenate (Supplemental Figure 6). The LC-MS/MS analysis identified 1120 proteins in the synaptosomes from WT and 1248 proteins in those from MPSIIIC 3-month-old mice (FDR ≤ 1%). In turn, 809 proteins were identified in synaptosomes of 6-month-old WT, and 1246 proteins, in synaptosomes of 6-month-old MPSIIIC animals (Figure 8A). In 3-month-old mice, 133 proteins were reduced in abundance in MPSIIIC synaptosomes as compared with WT, whereas 395 proteins were reduced at 6 months (Figure 8B and Supplemental Table 3). These proteins were then classified according to their biological function and linked to a particular metabolic or signaling pathway using automated gene ontology (GO) annotation (Figure 8, C and D) (43). The 3 protein groups that have major reductions in MPSIIIC synaptosomes at both 3 and 6 months of age were mitochondrial proteins (27.3% of all proteins), synaptic proteins (14%), and proteins involved in vesicle trafficking (5.5%). In particular, synaptic proteins that were reduced in MPSIIIC mice at both ages (Figure 9A) and/or showed further reduction with age included the following proteins: syntaxin-binding protein 1 (Stxbp1/Munc18-1), responsible for docking and fusion of synaptic vesicles in the synaptic terminal; Syn1 and synapsin 2 (Syn2), that coat synaptic vesicles and function in the regulation of neurotransmitter release; calcium/calmodulin-dependent protein kinase II α (Camk2a), a member of the NMDAR signaling complex in excitatory synapses that functions in long-term potentiation and neurotransmitter release; and Nlgn2, a transmembrane protein that acts on the recruitment and clustering of synaptic proteins. In contrast to Nlgn1, which is specific to glutamatergic synapse and is mislocalized in MPSIIIC cultured neurons (Figure 4D), Nlgn2 is exclusively localized to inhibitory synapses (44). Specifically, Stxbp1 was reduced by 43.1% in MPSIIIC synaptosomes from 3-month-old mice and by 50% by the age of 6 months. Syn1 was 29.8% lower at 3 months and reached 41.5% reduction by the age of 6 months. Syn2 was reduced by 51.6% at the age of 6 months, Camk2a was reduced by 22.4% at 3 months, and Nlgn2 was reduced by 83.3% at 3 months. In general, deficiency of Syn1, PSD-95, and Nlgn1 detected by proteomics was consistent the results of immunocytochemistry. Significant reduction of Syn1, Stxbp1, and Camk2a in the brain (frontal part) homogenates from 6-month-old MPSIIIC mice has been also independently confirmed by immunoblots (Figure 9B).

The second group of proteins that was significantly changed in MPSIIIC neurons consisted of mitochondrial proteins and enzymes. Multiple proteins were reduced in MPSIIIC at the age of 3 months (Figure 9C), with a further drastic decrease by the age of 6 months, when many of them were diminished to below the detection levels for the LC-MS/MS technique. This confirmed progressive deficiency of mitochondrial function, which we previously described in the neurons of MPSIIIC mice (13).

The third major group of proteins deficient or reduced in the synaptosomes from MPSIIIC mouse brains is involved in vesicle trafficking and endocytosis (Figure 9D). In particular, we observed major alterations in the levels of clathrin heavy chain 1 (Cltc/CHC) and dynamin-1 (Dnm1). Cltc was reduced by 32% at 3 months and by 42% at 6 months in the MPSIIIC synaptosomes. Cltc reduction in the MPSIIIC mouse brain homogenates was confirmed by immunoblot (Figure 9B). Dnm1 was reduced by 39% at 3 months but increased by 46% at 6 months. Subunits AP2a2 and AP2b1 of the adaptor protein complex 2 (AP-2) involved in recruiting CHC for reformation of synaptic vesicles (45) were also reduced in MPSIIIC brains at the age of 3 months. The NSF attachment protein β (Napb) was significantly reduced in MPSIIIC synaptosomes at 6 months. Napb belongs to the group of SNAP proteins that play a role in SNARE complex dissociation and recycling (synaptic vesicle docking), and its deficiency has been associated with the emerging of seizures in human patients (46) and in mouse models (47). Together, the deficiency of proteins involved in vesicle trafficking and endocytosis suggested that these processes could be affected in the MPSIIIC neurons. At the same time, the majority of abundant neuronal proteins were similar in WT and MPSIIIC mice (Supplemental Table 4) demonstrating that the changes were specific for the above groups of proteins.

### MPSIIIC hippocampal neurons have impaired synaptic vesicle trafficking and turnover.

**Figure 10:**
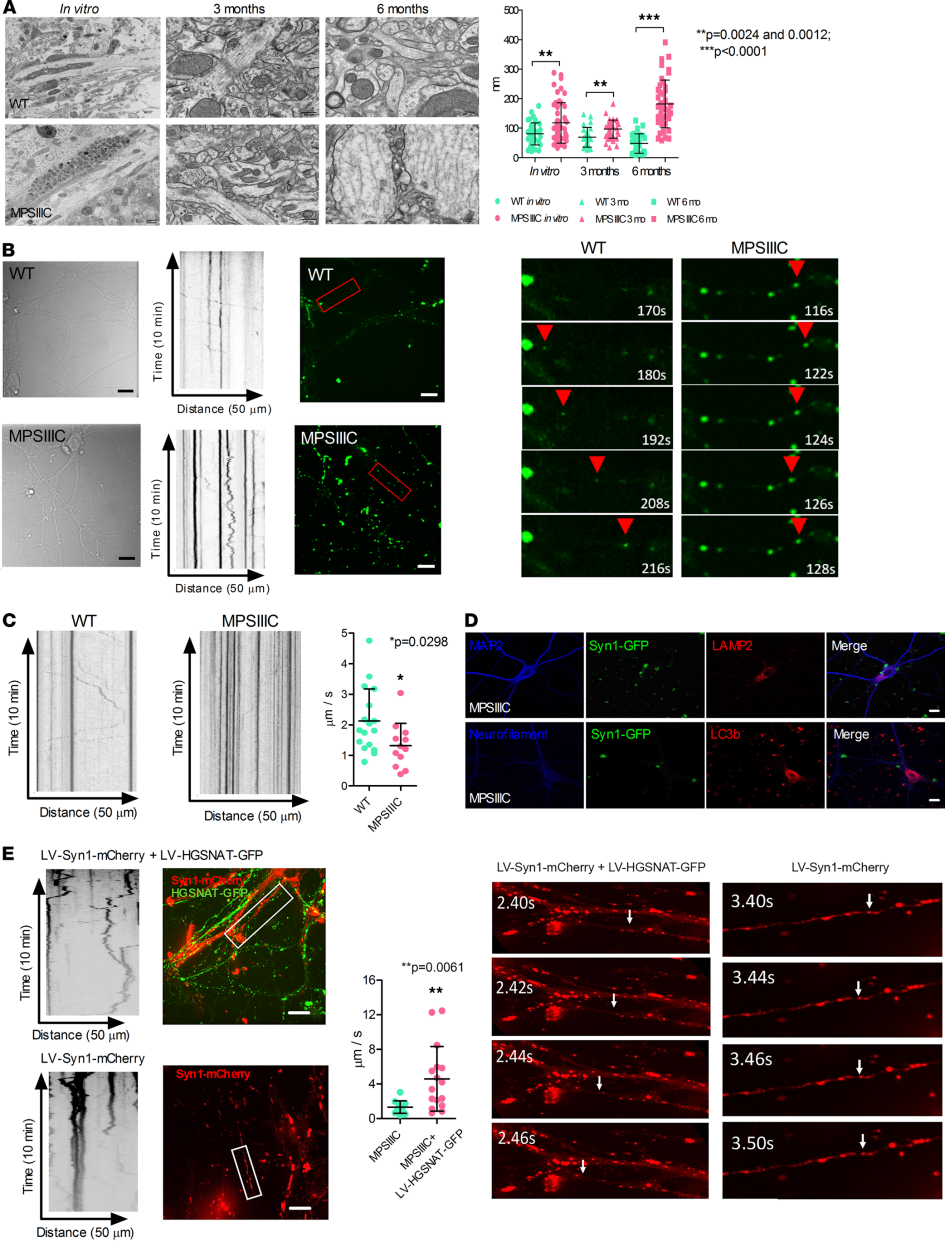
Vesicle transport defects in MPSIIIC neurons. (**A**) Microtubules in MPSIIIC hippocampal cultured neurons are disorganized, sparse, and nonparallel, with storage vacuoles between the filaments. Graphs show individual distances between adjacent microtubules, mean ± SD from at least 30 cells from 3 experiments with different neuronal cultures or 3 different animals per genotype. *P* values were calculated using 2-tailed *t* test. (**B**) Bright-field images (left), fluorescence images (right), and kymographs of Syn1^+^ vesicles (middle) in WT and MPSIIIC neurons transduced with LV-Syn1-GFP. In MPSIIIC neurons, moving GFP^+^ vesicles show a wiggling pattern, while in WT cells, the majority of vesicles travel in 1 direction (red arrowhead). (**C**) GFP^+^ vesicles in MPSIIIC neurons have a slower speed. Videos were recorded every 2 seconds for 10 minutes. The bar graph shows the speed of individual Syn1^+^ vesicles, mean ± SD measured in 17 WT and 12 MPSIIIC cells in 3 different sets of experiments for each genotype; *P* value was calculated by 2-tailed *t* test. (**D**) The Syn1-GFP^+^ granules in MPSIIIC neurons do not colocalize with LAMP2 or LC3. (**E**) Fluorescence images (left) and kymographs (right) of Syn1-mCherry^+^ vesicles in MPSIIIC hippocampal neurons transduced with LV-Syn1-mCherry or cotransduced with LV-Syn1-mCherry and LV-HGSNAT-GFP. In GFP^–^ neurons, moving mCherry^+^ vesicles show a wiggling pattern, while in GFP^+^ cells, the majority of moving vesicles travel in 1 direction (white arrowhead). mCherry^+^ vesicles in the MPSIIIC neurons expressing HGSNAT-GFP move at a higher speed than those in nontransduced MPSIIIC cells. Videos were recorded every 2 seconds for 10 minutes. The bar graph shows the speed of individual Syn1^+^ vesicles, mean ± SD measured in 10 nontransduced (17 vesicles) and 9 transduced (17 vesicles) MPSIIIC cells originating from 3 different sets of experiments. *P* value was calculated by 2-tailed *t* test. Scale bars: 500 nm (**A**) and 10 μm (**B**, **D**, and **E**).

Besides reduced density of synaptic vesicles and smaller areas/lengths of PSD, TEM analysis also identified defects of microtubules in the MPSIIIC hippocampal neurons. Most of the microtubules were disorganized, sparse, and nonparallel, with multiple storage bodies present between the microtubule filaments (Figure 10A). In both cultured neurons and in CA1 neurons of MPSIIIC mice at 3 and 6 months, the average distance between microtubules was significantly increased when compared with WT cells, and this defect was aggravated with age (Figure 10A, right panel). This observation, together with reduced levels of proteins involved in vesicle targeting and turnover observed by proteomic analysis, suggested that the low density of synaptic vesicles at the axonal terminals could be caused by impaired vesicular trafficking of synaptic vesicle precursor organelles transported from the cell body. To test this hypothesis, we studied the axonal trafficking of synaptic vesicle precursors. On DIV3, cells were transduced with LV encoding for Syn1-GFP. On DIV21, 10-minute videos were recorded using a spinning disk microscope to visualize trafficking of GFP^+^ vesicles (Figure 10B). In MPSIIIC neurons, the majority of Syn1-GFP^+^ vesicles remained stationary or wiggled back and forth instead of showing a constant motion in 1 direction (Figure 10B and Supplemental Video 1). Conversely, in WT neurons, the moving vesicles were progressing in a stable direction (Figure 10B and Supplemental Video 2), and their speed was faster as compared with that of vesicles in MPSIIIC cells (Figure 10C). In addition, while most of Syn1-GFP in WT neurons were associated with fine punctate distributed over the axons, in the MPSIIIC neurons, they were localized in coarse intensely stained spheroid granules (Figure 10B). This type of staining was consistent with the hypothesis that Syn1-GFP is accumulated along the axon. These bodies were negative for the lysosomal marker LAMP2, or autophagosomal marker LC3, suggesting that the inclusions containing Syn1-GFP were different from lysosomal storage bodies or autophagosomes (Figure 10D).

We further attempted to rescue the defects in movement of Syn1^+^ vesicles by a virus overexpressing the WT human HGSNAT enzyme in order to see if this phenotype is associated with HGSNAT deficiency. The MPSIIIC mouse neurons were transduced at DIV3 with above-described LV-HGSNAT-GFP vector and at DIV5 with LV encoding for Syn1-mCherry. On DIV21, movement of mCherry^+^ vesicles was recorded in the processes of neurons either positive or negative for the expression of HGSNAT-GFP protein. Our results showed that, while most of mCherry^+^ vesicles in axons of HGSNAT-GFP-negative MPSIIIC neurons were either stationary or wiggled back and forth, the vesicles in the neurons expressing HGSNAT-GFP showed a constant motion in 1 direction and with increased speed (Figure 10E and Supplemental Videos 3 and 4), indicating that WT active HGSNAT rescues vesicle movement defects.

## Discussion

Neurodegeneration, neuroinflammation, defects in autophagy, and microgliosis are commonly though to be the major causes of behavioral alterations and developmental delay in patients with neurological LSD. Our current data demonstrate that both excitatory and inhibitory synaptic inputs to the pyramidal CA1 hippocampal neurons are drastically reduced in the mouse model of Sanfilippo C syndrome already at P14–P20, at least 2–3 months before the development of other neuronal pathologies such as neuroinflammation, mitochondrial damage as well as neuronal accumulation of simple gangliosides and misfolded proteins (13). We observed that the amplitude and frequency of both mEPSCs and mIPSC are reduced at both P14–P20 and P45–P60. For comparison, in mouse models of MPSIIIA, only slightly reduced mEPSC amplitude, with no changes in frequency, was detected in the somatosensory cortex (23), while in the mouse model of Niemann-Pick type C disease, neurons presented enhanced glutamatergic neurotransmission in the hippocampus, leading to hyperexcitability (48). Our electrophysiological data closely parallel the marked reduction of the VGLUT1/PSD-95 colocalization as observed in cultured MPSIIIC neurons and in the hippocampal and cortical neurons of MPSIIIC mice at ages of P10, 3 months, and 6 months, together suggesting an overall synaptic deficit that aggravates with age. Moreover, the density of dendritic synaptic spines of pyramidal CA1 hippocampal neurons was reduced already at P10 and never reached the levels observed in WT mice. Drastically reduced levels of synaptic vesicles, including the docking vesicles, in the terminals and smaller sizes and areas of excitatory postsynaptic densities were also found both in pyramidal CA1 hippocampal neurons at 3 and 6 months of age and in cultured hippocampal neurons at DIV21. These changes affect mainly excitatory circuits, and PSD-95 seems to be one of the most severely reduced biomarkers not only in the mouse model, but also in all studied postmortem cortical tissues of neurological MPSI, -II, -IIIA, -IIIC, and -IIID patients. Importantly, the levels of this protein in mouse MPSIIIC neurons can be rescued in vivo and in vitro by correcting the primary genetic defect, with the viral vectors expressing the WT human HGSNAT enzyme. This implicates genetic defects in HGSNAT as a primary cause of synaptic deficits, but it also suggests that improvements in the behavior of MPSIIIC mice treated by striatal administration of AAV-HGSNAT vectors (41) could be at least partially explained by the rescue of synaptic defects.

Major changes in the synaptic morphology and the levels and distribution of synaptic markers found in the hippocampus (i.e., scarcity of synaptic vesicles in the excitatory terminals, reduced PSD length, and reduced density of mature mushroom-shaped spines) were mainly recapitulated in dissociated neuronal cultures. In contrast, the alterations in excitatory or inhibitory neurotransmission had different features in vivo and in vitro. Miniature synaptic currents from hippocampal cultured neurons revealed no significant changes in amplitude or frequency of mIPSCs, whereas an almost 3-fold increased frequency was detected in mEPSCs. In contrast, our acute hippocampal slice recordings showed reduction of both frequency and amplitude of mEPSCs and mIPSCs, suggesting that both excitatory and inhibitory neurotransmission are deficient in MPSIIIC mice. It is tempting to speculate that changes in GABAergic synaptic transmission occurring in vivo*,* but not in neuronal cultures, appear due to an attempt to compensate for defects in excitatory transmission. Furthermore, the change in frequency and lack of a significant change in the kinetics of the events suggests a possible presynaptic locus of deficit. This latter observation is also in agreement with reduced density of synaptic vesicles in the axonal terminals in MPSIIIC mice as compared with WT.

We hypothesize that the increase in mEPSCs observed in cultured MPSIIIC neurons, but not in vivo, is linked to the major changes in the positioning of synapses on dendrites detected by both confocal and TEM. While the overwhelming majority of synapses in WT cultures were axospinous, in MPSIIIC neurons, > 75% of the synapses were axodendritic. Since MPSIIIC cultured neuron dendrites present mainly immature filopodia spines that lack the proper postsynaptic machinery for synaptic function (49), we speculate that they reallocate their excitatory synapses to the dendrites, altering their response to the presynaptic signaling, which could explain the increased frequency and amplitude of mEPSC observed in neuronal cultures. Such changes have not been observed in mouse brains, consistent with the reduction of both amplitude and frequency of miniature postsynaptic currents.

The disruption of the microtubule architecture in the axons of cultured MPSIIIC neurons (disorganized, sparse, and nonparallel microtubules), together with reduced levels of proteins involved in vesicle trafficking and endocytosis in the synaptosomes from MPSIIIC mouse brains, prompted us to hypothesize that the scarcity of synaptic vesicles in the axonal terminals can be caused by partially impaired trafficking of synaptic vesicle precursors from the soma. Indeed, direct analysis of the axonal movement of Syn1^+^ vesicles by live imaging microscopy demonstrated that, in MPSIIIC neurons, fewer vesicles were moving in a stable direction toward the terminal. Besides, vesicles that were moving in 1 direction had slower speed than that of vesicles in WT neurons. These movement defects of Syn1^+^ vesicles in MPSIIIC neurons were rescued by restoring HGSNAT activity, which linked these defects to the primary enzyme deficiency.

Interestingly, the majority of newly synthesized Syn1-GFP in MPSIIIC neurons was localized in coarse spheroid granules distributed along the axon. Formation of focal granular enlargements within axons (axonal spheroids or torpedoes; neuroaxonal dystrophy) and dendrites is a phenomenon described in a variety of LSDs including mannosidosis, G_M1_ and G_M2_ gangliosidosis, prosaposin deficiency, and Niemann-Pick type C (20, 21, 50–53). In the axons, spheroids often contained electron-dense concentric lamellar bodies and neurofilaments (53).

The mechanism underlying formation of the axonal spheroids in LSD is not completely understood. They could be formed by merging of lysosomes in the proximal axon since accumulation of storage materials in lysosomes impairs their onward BORC-dependent axonal transport (54). Besides, the block in anterograde transport of lysosomes prevents their merging with early endosomes and autophagosomes in the axon terminal (55). As a result, the dynein-mediated retrograde transport of autophagosomes and their maturation to autolysosomes (56) is impaired, causing their accumulation in distal axon and formation of axonal spheroids. Our data, however, show that the lysosomal storage bodies are primarily found in the soma, and axonal Syn1^+^ spheroid granules are negative for the markers of endo-lysosomal pathway or for the autophagosomal marker LC3. We speculate, therefore, that they appear in the process of accumulation of the synaptic vesicle precursors at the places in the axons where the transport is blocked due to the microtubule defects.

Together, our experiments demonstrate that lysosomal storage in MPSIIIC neurons results in appearance of drastic synaptic defects in CA1 pyramidal neurons appearing in mice as early as at P10, which corresponds to the human developmental age of 2.5–3 years — i.e., the time when a majority of MPSIIIC patients show an onset of symptoms. Further studies are necessary to define the exact mechanism; however, based on the fact that scarcity of synaptic spines were also observed in CA1 neurons of sialidosis and Tay-Sachs disease, we speculate that synaptic defects are not dependent on HS accumulation. Our current results suggest that the defects are caused by the general appearance of lysosomal storage bodies in axons, which disrupts microtubule structure and normal transport of vesicles carrying the synaptic proteins (57). This, in concert with impaired autophagy and endocytosis at the axonal terminals, could result in the deficit of synaptic vesicles affecting synaptic transmission and plasticity. Reduction of PSD-95^+^ puncta was detected in postmortem cortices of human MPS patients, suggesting that similar excitatory synaptic defects can also exist in other neurological lysosomal diseases. In light of these findings, it is tempting to speculate that drugs known to enhance excitatory synaptic transmission should be tested for their ability to improve synaptic function, as well as behavioral and cognitive defects, in MPSIIIC mice and in other animal models of neurological LSD. If confirmed, such synaptic targeted treatment — although it would not eliminate the lysosomal storage and substitute for gene correction — may, nevertheless, improve behavioral deficits in patients with advanced form of the disease.

## Methods

Supplemental Methods are available online with this article.

### Mouse models.

The mouse models of MPSIIIC (*Hgsnat-Geo*), sialidosis (*Neu1^–/–^*) and Tay-Sachs (*Hexa*^–/–^) have been previously described (13, 32–34). The Thy1-EGFP transgene mice were obtained from The Jackson Laboratory (JAX stock no. 007788) (28). The experiments were conducted for both male and female mice, and the data were analyzed to determine the differences between sexes. Because no differences between sexes were observed in the experiments conducted in this study, the data for male and female mice were combined. The animals were bred as homozygous couples for both WT and KOs. The neuronal cultures were established from pooled hippocampi of mouse embryos of both sexes at E16. Frozen or fixed cerebral cortices from clinically confirmed MPS patients (1 case of MPSI, 1 case of MPSII, 2 cases of MPSIIIA, 1 case of MPSIIIC, and 2 cases of MPSIIID) and age-matched controls with no pathological changes in the CNS were provided by NIH NeuroBioBank (project 1071, MPS Synapse). Whole-cell patch clamp recordings in dissociated hippocampal neuronal cultures after DIV19-22 were conducted on at least 8 dissociated hippocampal neuronal cultures per genotype each time established from at least 3 mice. Whole cell recordings in acute hippocampal slices were conducted essentially as described (58). For live imaging, primary hippocampal neurons were plated on poly-L-lysine–coated chambered glass slides, transduced with LV-Syn1-GFP at DIV3, and imaged at DIV21 using an inverted spinning disk confocal microscope. To study the rescue of vesicle movement defects, the neurons were transduced with LV-HGSNAT-GFP at DIV3 and with LV-Syn1-mCherry at DIV5.

### Availability of data and materials.

All data generated or analyzed during this study are included in this published article and its supplemental material.

### Statistics.

Statistical analyses were performed using GraphPad Prism 9.0.0. software (GraphPad Software, San Diego, CA). The normality for all data was checked using the D’Agostino and Pearson omnibus normality test. Significance of the difference was determined using 2-tailed *t* test (normal distribution) or Mann-Whitney test, when comparing 2 groups. One-way ANOVA test, followed by the Bonferroni or Tukey multiple comparison test (normal distribution), or Kruskal-Wallis test, followed by the Dunn multiple comparison test, was used when comparing >2 groups. Two-way ANOVA, followed by Bonferroni post hoc test, was used for 2-factor analysis. *P* ≤ 0.05 was considered significant. Statistical analyses of the LC/MS data were performed using the Scaffold software v. 4.8.1.

### Study approval.

All of the experiments performed on mice have been approved by the IACUC of the Ste-Justine Hospital Research Center. Ethical approval for the research involving human tissues was granted by Research Ethics Board (Comité d’éthique de la recherche) of CHU Ste-Justine. Frozen or fixed with PFA, cerebral cortices from clinically confirmed MPS patients (1 case of MPSI, 1 case of MPSII, 2 cases of MPSIIIA, 1 case of MPSIIIC, and 2 cases of MPSIIID), and age-matched controls with no pathological changes in the CNS were provided by NIH NeuroBioBank (project 1071, MPS Synapse) together with clinical descriptions and results of the neuropathological examination. Families provided the informed consent prior to donation of tissues.

## Author contributions

CP, LB, PB, XP, MT, CH, EF, EB, and CO conducted experiments and acquired data; CP, LB, PB, XP, CH, EF, EB, CO, PSM, JCL, CRM, GDC, and AVP analyzed data; BB provided reagents; CP, PB, and AVP wrote the first draft of the manuscript; and AVP, CP, JCL, GD, PSM, PT, BB, and CRM edited the manuscript. All authors read and approved the final manuscript.

## Supplementary Material

Supplemental data

Supplemental Video 1

Supplemental Video 2

Supplemental Video 3

Supplemental Video 4

## Version 1 06/22/2021

In-Press Preview

## Version 2 08/09/2021

Electronic publication
