# Supplementary material for: Early defects in mucopolysaccharidosis type IIIC disrupt excitatory synaptic transmission
Source: JCI Insight. 2021 Aug 9;6(15):e142073. doi: 10.1172/jci.insight.142073 (PMC8410035; doi:10.1172/jci.insight.142073)
Supplement: Supplemental data [file jciinsight-6-142073-s137.pdf]

Materials and Methods

Animals. The mouse models of MPSIIIC (*Hgsnat-Geo*), sialidosis (*Neu1*$^{-/-}$) and Tay-Sachs (*Hexa*$^{-/-}$) in C57BL/6J genetic background have been previously described (1-4). The Thy1-EGFP transgene mice expressing enhanced green fluorescent protein (EGFP) under the control of a modified regulatory region of the mouse thy1.2 gene promoter (containing the sequences required for neuronal expression but lacking the sequences required for expression in non-neural cells) were obtained from The Jackson Laboratory (JAX stock #007788)(5). All mice were bred and maintained in compliance with the Canadian Council on Animal Care (CCAC) in the accredited animal facility of the CHU Sainte-Justine. Mice were housed in an enriched environment with continuous access to food and water, under constant and controlled temperature and humidity, on a 12 h light/dark cycle. All the experiments performed on mice have been approved by the Animal Care and User Committee of the Ste-Justine Hospital Research Center. The experiments were conducted for both male and female mice and the data analyzed to determine the differences between sexes. Because no differences between sexes were observed in the experiments conducted in this study the data for males and females were combined. The animals were bred as homozygous couples for both WT and knockouts. The neuronal cultures were established from pooled hippocampi of mouse embryos of both sexes.

Neuronal cultures and transduction. Primary hippocampal neuronal cultures were established from mouse brain tissues at embryonic day 16. The hippocampi were dissected, treated with 2.5% trypsin (Sigma-Aldrich, T4674) for 15 minutes at 37°C, washed 3 times with Hank's Balanced Salt Solution (HBSS, Gibco, 14025-092) and mechanically dissociated using borosilicate pipets with opening sizes of 3, 2 and 1 mm. The cells were then counted with the viability dye trypan blue (Thermo Fisher Scientific, 15250061) in a hemocytometer and

resuspended in Neurobasal media (Gibco, 21103-049) supplemented with B27, N2, penicillin and streptomycin. The hippocampal cells were plated at a density of 60,000 cells per well in a 12-well plate, on Poly-L-Lysine hydrobromide (Sigma Aldrich, P9155) coated coverslips. Cells were cultured for 21 days and 50% of media was changed on days 3, 10 and 17. Primary hippocampal neurons were transduced at day *in vitro* (DIV) 3 with lentivirus (LV) encoding for HGSNAT fused to green fluorescent protein (GFP) under a cytomegalovirus (CMV) promoter, for the rescue experiments, or with LV encoding for synapsin 1 (Syn1) fused to GFP (GeneCopoeia, Inc., cat.# LPP-Z5062-Lv103-100), for live-cell imaging. Transduction was performed in the presence of 8 μg/ml of protamine sulfate for 15 h.

Analyses of postsynaptic densities (PSD) in human brain tissues. Frozen or fixed with paraformaldehyde (PFA), cerebral cortices from clinically confirmed MPS patients (1 case of MPSI, 1 case of MPSII, 2 cases of MPSIIIA, 1 case of MPSIIIC and 2 cases of MPSIIID) and age-matched controls with no pathological changes in the central nervous system were provided by NIH NeuroBioBank (project 1071, MPS Synapse). Upon arrival to the laboratory, the samples were embedded in Tissue-Tek® optimum cutting temperature (OCT) Compound and stored in -80°C. Brains were cut in 40 μm sections and stored in cryopreservation buffer (0.05 M sodium phosphate buffer pH 7.4, 15% sucrose, 40% ethylene glycol) at -20°C until labelling for immunohistochemistry.

Immunocytochemistry and immunohistochemistry. Cultured neurons at DIV21 were fixed in 4% PFA/4% sucrose solution in PBS, pH 7.4, washed 3x with PBS and stored at 4°C for posterior analysis. Mouse brains were collected from animals perfused with PBS followed by perfusion with 4% PFA and left in 4% PFA overnight. Brains were placed in 30% sucrose for 2 days and then they were embedded in Tissue-Tek® OCT Compound and stored in -80°C. Brains

were cut in 40 µm sections and stored in cryopreservation buffer (0.05M sodium phosphate buffer pH 7.4, 15% sucrose, 40% ethylene glycol) at -20°C until labelling. The cultured neurons were permeabilized with 0.1% Triton X-100 and blocked with 5% goat serum in PBS for 1 h, and then incubated overnight at 4°C with primary antibodies in 5% goat serum. Brain sections (mouse or human) were washed 3 times with PBS, and permeabilized/blocked by incubating in 5% bovine serum albumin (BSA) in 1% Triton X-100 for 2 h. Incubation with primary antibodies dissolved in 1% BSA in 0.1% Triton X-100 was performed overnight at 4°C. The antibodies used and their working concentrations are shown in the Table 1:

Table 1: List of antibodies

Antigen	Host/Target species	Dilution	Manufacturer
Synapsin-1	Rabbit anti-mouse	1:200	Abcam (ab64581)
Synaptophysin	Rabbit anti-mouse	1:300	Millipore Sigma (SAB4502906)
Lysosomal-associated membrane protein 1 (LAMP1)	Rat anti-mouse	1:200	DSHB (catalog 1D4B-s)
Heparan sulfate (10E4 epitope)	mouse anti-mouse	1:100	AMSBIO (F58-10E4)
NeuN	Rabbit anti-mouse	1:250	Millipore Sigma (MABN140)
β-tubulin III	Mouse anti-mouse	1:400	Millipore Sigma (T2200)
G_{M2}	Mouse humanized	1:400	KM966
Microtubule-associated protein 2 (MAP2)	Chicken anti-mouse	1:2000	Abcam (ab5392)

Vesicular glutamate transporter 1 (VGLUT1)	Rabbit anti-mouse	1:1000 (cells) and 1:200 (tissues)	Abcam (ab104898)
Postsynaptic density protein 95 (PSD-95)	Mouse anti-mouse	1:1000 (cells) and 1:200 (tissues)	Abcam (ab99009)
Vesicular gamma aminobutyric acid (GABA) transporter (VGAT)	Rabbit anti-mouse	1:1000	Synaptic Systems (#131003)
Gephyrin	Mouse anti-mouse	1:1000	Synaptic Systems (#147021)
Neuroligin-1	Rabbit anti human, mouse, rat	1:300	Abcam (ab26305)
Neurofilament medium chain	Mouse anti-mouse	1:200	DSHB (2H3-s)
IgG	Goat anti-rabbit, anti-mouse, anti-rat or anti-chicken Alexa Fluor 488-, Alexa Fluor 555- or Alexa Fluor 633-conjugated	1:1000 (cells) and 1:400 (tissues)	Thermo Fisher Scientific

The cells and tissues were washed 3x with PBS and counterstained with Alexa Fluor for 1 h for cells or 2 h for tissues at room temperature and the nuclei were stained with Draq5 (1:1000,

Thermo Fisher Scientific) for 40 min or 4′,6-diamidino-2-phenylindole (DAPI) within the mounting media.

To visualize the synaptic spines in cultured neurons, on DIV21 the culture media was replaced by 2.5 μM 1,1'-Dioctadecyl-3,3,3',3'-Tetramethylindocarbocyanine Perchlorate (Dil, Thermo Fisher Scientific) dissolved in fresh media and the cells were incubated for 15 min at 37 ^{o}C, washed 3x with media preheated to 37 ^{o}C, and fixed with 1.5% PFA in PBS for 15 min. The slides were mounted with Prolong Gold Antifade mounting reagent (Thermo Fisher Scientific) and analyzed using Leica DM 5500 Q upright confocal microscope (63x oil objective, N.A. 1.4, zoom 1.5). Images were processed and quantified using ImageJ 1.50i software (National Institutes of Health, Bethesda, MD, USA). Quantification was double-blinded and performed for at least 3 different experiments. Quantifications of synaptic spine density and puncta were performed in 20 μm of dendrite length, respectively, 30 μm away from the neuronal soma.

Transmission electron microscopy. At 3 and 6 months, 3 mice per genotype were anesthetized with sodium pentobarbital and transcardiacally perfused with PBS followed by 2.5% glutaraldehyde in 0.2 M phosphate buffer (pH 7.2). The brains were extracted and post fixed in the same fixative for 24 h at 4°C. The hippocampi were dissected in blocks of 1 mm^3 and sections of 1 μm thickness were cut, mounted on glass slides, stained with toluidine blue and examined on a Leica DMS light microscope to select the CA1 region of the hippocampus for the electron microcopy. For the neuronal cultures, cells at DIV21 were washed with PBS and fixed overnight in 2.5% glutaraldehyde in 0.1 M sodium cacodylate buffer (pH 7.4, Electron Microscopy Sciences) at 4°C. Both tissue and cultured cell samples were stained with 1% osmium tetroxide (Mecalab) and 1.5 % potassium ferrocyanide (Thermo Fisher Scientific) followed by dehydration in a graded series of ethanol (30%-90%) and embedded in Epon. The polymerized blocks were trimmed, and

100 nm ultrathin sections were cut with an Ultracut E ultramicrotome (Reichert Jung), mounted on 200-mesh copper grids (Electron Microscopy Sciences), stained with uranyl acetate (Electron Microscopy Sciences) and lead citrate (Thermo Fisher Scientific) and examined on a FEI Tecnai 12 transmission electron microscope (FEI Company) operating at an accelerating voltage of 120 kV equipped with an XR-80C AMT 8 megapixel CCD camera. For quantification, the micrographs were taken with 13,000 x and 49,000 x magnification.

Whole-cell patch clamp recordings in dissociated hippocampal neuronal cultures. Experiments were performed after DIV19-22 on at least 8 dissociated hippocampal neuronal cultures per genotype each time established from at least 3 mice. Cultures were maintained in a humidified atmosphere of 5% CO_2 and 95% O_2 at 37°C. Coverslips with cultured hippocampal neurons were placed in a recording chamber mounted on an inverted microscope (Nikon Eclipse Ti-S). Whole-cell recordings were obtained from neurons, selected by visual identification in phase contrast, using borosilicate pipettes (3–6 MΩ). The intracellular solution for recording miniature excitatory postsynaptic currents (mEPSCs) and inhibitory postsynaptic current (mIPSCs) contained (in mM) 132 $CsMeSO_3$, 8 CsCl, 0.6 EGTA, 10 diNa-phosphocreatine, 10 HEPES, 4 ATP-Mg^{2+}, 0.4 GTP-Na_3. The solution (275-280 mOsmol) was filtered and adjusted to pH 7.25-7.30 with CsOH. mEPSCs and mIPSCs were recorded in the presence of tetrodotoxin (TTX) (1 µM; Abcam) in artificial cerebrospinal fluid (ACSF) containing (in mM) 132.3 NaCl, 3 KCl, 15 HEPES, 1.25 NaH_2PO_4, 2 $CaCl_2$, 2 $MgSO_4$, 10 D-Glucose. ACSF was adjusted to pH 7.37-7.41 with NaOH and perfused at 1 ml/min. Recordings were obtained using a Multiclamp 700B amplifier and a 1440A Digidata acquisition board (Molecular Devices). Signals were low-pass-filtered at 2 kHz, digitized at 20 kHz and stored on a PC. Upon whole cell configuration, holding potential was initially maintained at -70 mV for mEPSCs and subsequently reduced to 0

mV for mIPSCs to obtain 5 min quality recordings of each in the same cell. Signals were routinely monitored, and recordings were only included if access resistance was less than 30 MΩ and varied for less than 25%. For analysis, mEPSCs and mIPSCs were Bessel filtered at 2.8 kHz using pClamp10 software (Molecular Devices). Miniature events were analyzed using MiniAnalysis (Synaptosoft). Two sided unpaired tests were used for statistical comparison of mEPSC and mIPSC frequencies and amplitudes. Where data was normally distributed (mEPSC frequency) Student's t-test were used, where data was not normally distributed (mEPSC amplitude, mIPSC frequency and amplitude) Mann-Whitney tests were used. For cumulative probability distribution analysis, data was binned into 5% intervals with 20-300 events per cell distributed in 20 bins. Significance of the probability distributions between groups was assessed by Kolmogorov-Smirnov tests.

Whole cell recordings in acute hippocampal slices. Acute hippocampal slices were prepared as described (6). Briefly, animals were anaesthetized deeply with isoflurane and decapitated. The brain was dissected carefully and transferred rapidly into an ice-cold (0–4°C) solution containing the following (in mM): 250 sucrose, 2 KCl, 1.25 NaH_2PO_4, 26 $NaHCO_3$, 7 $MgSO_4$, 0.5 $CaCl_2$ and 10 glucose, pH 7.4. The solution was oxygenated continuously with 95% O_2 and 5% CO_2, 330–340 mOsm/L. Transverse hippocampal slices (thickness, 300 µm) were cut using a vibratome (VT1000S; Leica Microsystems), transferred to a bath at room temperature (23°C) with standard ACSF at pH 7.4 containing the following (in mM): 126 NaCl, 3 KCl, 1 NaH_2PO_4, 25 $NaHCO_3$, 2 $MgSO_4$, 2 $CaCl_2$, 10 glucose, continuously saturated with 95% O_2 and 5% CO_2 and allowed to recover for 1 h. During the experiments, slices were transferred to the recording chamber at physiological temperature (30–33 °C) continuously perfused with standard ACSF, as described above, at 2 ml/min. Pyramidal CA1 neurons from the hippocampus were

identified visually using a 40X water immersion objective. Whole-cell patch-clamp recordings were obtained from single cells in voltage- or current-clamp mode and only 1 cell per slice was recorded to enable post-hoc identification and immunohistochemical processing. Recording pipettes (4–6 MΩ) were filled with a K-gluconate based solution for voltage-clamp recordings (in mM): 130 K-gluconate, 10 KCl, 5 diNa-phosphocreatine, 10 HEPES, 2.5 $MgCl_2$, 0.5$CaCl_2$,1 EGTA, 3 ATP-Tris, 0.4 GTP-Li, 0.3% biocytin, pH 7.2–7.4, 280–290 mOsm/L.

After obtaining whole cell configuration, passive membrane properties were monitored for 5 min and current clamp recordings were done to measure action potential characteristics. Slices were then perfused with 0.5 µM TTX (to isolate miniature events) for 3 mins before commencing voltage clamp recordings. Cells were voltage clamped at -70 mV for mEPSCs recording and then held at 0 mV (calculated from the reversal potential of Cl) for mIPSCs recording. Data acquisition (filtered at 2–3 kHz and digitized at 15 kHz; Digidata 1440A, Molecular Devices, CA, USA) was performed using the Axopatch 200B amplifier and the Clampex 10.6 software (Molecular Devices). Both mEPSCs and mIPSCs were recorded for 7 min and a running template on a stable baseline (minimum of 30 events) was used for the analysis of miniature events on MiniAnalysis. Clampfit 10.2 software was used for analysis of action potential characteristics and other passive membrane properties.

For some experiments, to verify that all mEPSCs are blocked, slices were perfused with 5 µM DNQX (6,7-dinitroquinoxaline-2,3-dione) and 50 µM AP5 in addition to the TTX while recording mEPSCs at -70 mV after addition of α-amino-3-hydroxy-5-methyl-4-isoxazolepropionic acid (AMPA) and N-methyl-D-aspartate receptor (NMDAR) blockers. Similarly, for some experiments, slices were perfused with 100 µM BMI (bicuculline methiodide) and 50 µM AP5 in addition to the TTX in the ACSF to verify that all mIPSCS are blocked at 0 mV.

Isolation of synaptosomes. At 3 and 6 months of age, mice (3 per genotype) were anesthetized with sodium pentobarbital and sacrificed by cranial dislodgement. Brains were removed, weighted and placed in a cold Dounce tissue grinder with 2 mL of Syn-PER Synaptic Protein Extraction Reagent (Thermo Scientific) per 200 mg of brain tissue in the presence of protease and phosphatase inhibitor cocktails (cOMplete Tablets EDTA-free and PhosSTOP EASYpack tablets, Roche). The brains were homogenized on ice with 10 slow strokes, transferred to centrifuge tubes and centrifuged at 1,200 *g* for 10 min at 4°C. The supernatants were collected and centrifuged at 15,000 *g* for 20 min at 4°C. The supernatant, corresponding to the cytosolic fraction was collected and the pellet (synaptosomes) was resuspended with the Syn-PER reagent (500 µL per 200-400 mg of brain tissue). The concentration of proteins in the synaptosomes was measured using the Bio-Rad Bradford kit.

Liquid Chromatography / Mass Spectrometry. Each sample was diluted with 630 µL of 50 mM ammonium bicarbonate containing 5 mM TCEP [Tris(2-carboxyethyl) phosphine hydrochloride; Thermo Fisher Scientific], 20 mM 2-chloroactamide and vortexed for 1 h at 37°C. One µg of trypsin was added, and digestion was performed for 8 h at 37°C. Digestion was stopped with the addition of 6.3 µL of trifluoroacetic acid (TFA). The extracted peptide samples were dried down and solubilized in 5% ACN-0.2% formic acid (FA). The samples were loaded on a home-made C18 precolumn (0.3-mm inside diameter [i.d.] by 5 mm) connected directly to the switching valve. They were separated on a home-made reversed-phase column (150-µm i.d. by 150 mm) with a 56-min gradient from 10 to 30% ACN-0.2% FA and a 600-nL/min flow rate on a Ultimate 3000 nano-LC connected to an Q-Exactive Plus (Thermo Fisher Scientific, San Jose, CA). Each full MS spectrum acquired at a resolution of 70,000 was followed by 12 tandem-MS (MS-MS) spectra on the most abundant multiply charged precursor ions. Tandem-MS experiments

were performed using collision-induced dissociation (HCD) at a collision energy of 27%. The data were processed using PEAKS 8.5 (Bioinformatics Solutions, Waterloo, ON) and a mouse database. Mass tolerances on precursor and fragment ions were 10 ppm and 0.01 Da, respectively. Variable selected posttranslational modifications were carbamidomethyl (C), oxidation (M), deamidation (NQ), and phosphorylation (STY). The data were visualized with Scaffold 4.8.7 (protein threshold, 99%, with at least 2 peptides identified and a false-discovery rate [FDR] of 1% for peptides).

Live-cell imaging. Primary hippocampal neurons were plated on poly-L-lysine coated 4-well chambered glass slides at a density of 6 x10^4 cells. Neurons were transduced with LV-Syn1-GFP (GeneCopoeia^{TM}) at DIV3 and were imaged at DIV21 using an inverted spinning disk confocal microscope (Leica DMi8, 63x objective). To study the rescue of vesicle movement defects the neurons were transduced with LV-HGSNAT-GFP at DIV3 and with LV-Syn1-mCherry (GeneCopoeia^{TM}) on DIV5. During image recording the cells were maintained in a humidified atmosphere of 5% CO_2 at 37°C. Digital images were acquired with an EM-CCD camera. Cells were recorded every 2 s for a total of 600 s for 5 z-stacks of 0.4 µm each. The videos were played with 10 frames per second, all stacks combined. Kymographs were generated by analyzing the traffic of synaptic vesicles using ImageJ 1.50i software (National Institutes of Health, Bethesda, MD, USA).

Western blots. Brain tissues (frontal part of a hemisphere, approximately 25% of the brain) were homogenized in RIPA buffer (50 mM Tris-HCl pH 7.4, 150 mM NaCl, 1% NP-40, 0.25% sodium deoxycholate, 0.1% SDS, 2 mM EDTA, 1 mM PMSF, Roche protease and phosphate inhibitor cocktails, 2.5 ml per 1 g of tissue). Neuronal cells grown in culture were scraped in the same buffer (0.1 ml per million cells). The homogenates were kept on ice for 30 min and

centrifuged at 13,000 RPM at 4°C for 25 minutes. The supernatant was collected and centrifuged again for 15 min. The resulting lysates were separated by SDS-PAGE on 8% gels. Western blot analyses were performed according to standard protocols using the following antibodies: synapsin 1 (rabbit, 1:2000, Abcam, catalog ab64581), PSD-95 (rabbit, 1:2000, Abcam, catalog ab18258), Munc18-1 (rabbit, 1:3000, Abcam, catalog ab3451), calcium-calmodulin kinase II (CaMKII, mouse, 1:2000, Abcam, catalog ab22609), clathrin heavy chain (rabbit, 1:12000, Abcam, catalog ab21679) and α-Tubulin (1:2000, mouse, DSHB, catalog 12G10). Equal protein loading was confirmed by Ponceau S staining and the bands were quantified using ImageJ software.

Production of the Lentivirus. Human *HGSNAT* codon-optimized cDNA (7) fused to a GFP (*HGSNAT-GFP*) was cloned into a pENTR1A vector (Invitrogen, 11813-011) and then transferred to a 3rd generation lentiviral vector plasmid (pLenti PGK Blast DEST (w524-1), Addgene, Plasmid #19065, (8)), using Gateway™ technology according to manufacturer's instructions. The LV was produced in HEK293T cells cultured in RPMI media (Gibco) to 70-90% confluency and co-transfected with REV 6 µg per plate, VSVG 7.8 µg per plate, pMDL (gag-pol), 15 µg per plate, and *Hgsnat*-GFP, 9 µg per plate plasmids. Polyethylenimine 40 µg/ml (PEI, Linear, MW 25000, Transfection Grade, Polysciences Inc.) was used as a transfection reagent and incubated overnight at 37°C, 5% CO_2. On the following day, the medium was replaced by Dulbecco's Modified Eagle's Medium (DMEM, Gibco™) supplemented with 10% fetal bovine serum (FBS, Wisent Inc.) and 1% Penicillin-Streptomycin (Gibco™) and the cells cultured for 30 hours at 37°C, 5% CO_2. Medium was collected, filtered using a 0.22 µm low protein binding filter and then centrifuged at 50,000 g for 2 h, at 10°C with maximum acceleration and slow brake. The pellet with the virus was resuspended in 100 µl PBS, aliquoted and stored at -80°C.

Statistical analysis. Statistical analyses were performed using GraphPad Prism 9.0.0. software (GraphPad Software, San Diego, CA). The normality for all data was checked using the D'Agostino and Pearson omnibus normality test. Significance of the difference was determined using t-test (normal distribution) or Mann-Whitney test, when comparing 2 groups. One-way ANOVA test, followed by the Bonferroni or Tukey multiple comparison test (normal distribution), or Kruskal-Wallis test, followed by the Dunn multiple comparison test, was used when comparing >2 groups. Two-way ANOVA, followed by Bonferroni post hoc test, was used for 2-factor analysis. $P \leq 0.05$ was considered significant. Statistical analyses of the LC/MS data were performed using the Scaffold software v. 4.8.1.

1. Martins C, Hulkova H, Dridi L, Dormoy-Raclet V, Grigoryeva L, Choi Y, et al. Neuroinflammation, mitochondrial defects and neurodegeneration in mucopolysaccharidosis III type C mouse model. Brain. 2015;138(Pt 2):336-55.
2. Hansen GM, Markesich DC, Burnett MB, Zhu Q, Dionne KM, Richter LJ, et al. Large-scale gene trapping in C57BL/6N mouse embryonic stem cells. Genome Res. 2008;18(10):1670-9.
3. Phaneuf D, Wakamatsu N, Huang JQ, Borowski A, Peterson AC, Fortunato SR, et al. Dramatically different phenotypes in mouse models of human Tay-Sachs and Sandhoff diseases. Hum Mol Genet. 1996;5(1):1-14.
4. Pan X, De Aragao CBP, Velasco-Martin JP, Priestman DA, Wu HY, Takahashi K, et al. Neuraminidases 3 and 4 regulate neuronal function by catabolizing brain gangliosides. FASEB J. 2017;31(8):3467-83.

5. Feng G, Mellor RH, Bernstein M, Keller-Peck C, Nguyen QT, Wallace M, et al. Imaging neuronal subsets in transgenic mice expressing multiple spectral variants of GFP. Neuron. 2000;28(1):41-51.

6. Croce A, Pelletier JG, Tartas M, Lacaille JC. Afferent-specific properties of interneuron synapses underlie selective long-term regulation of feedback inhibitory circuits in CA1 hippocampus. J Physiol. 2010;588(Pt 12):2091-107.

7. Tordo J, O'Leary C, Antunes A, Palomar N, Aldrin-Kirk P, Basche M, et al. A novel adeno-associated virus capsid with enhanced neurotropism corrects a lysosomal transmembrane enzyme deficiency. Brain. 2018.

8. Campeau E, Ruhl VE, Rodier F, Smith CL, Rahmberg BL, Fuss JO, et al. A versatile viral system for expression and depletion of proteins in mammalian cells. PLoS One. 2009;4(8):e6529.

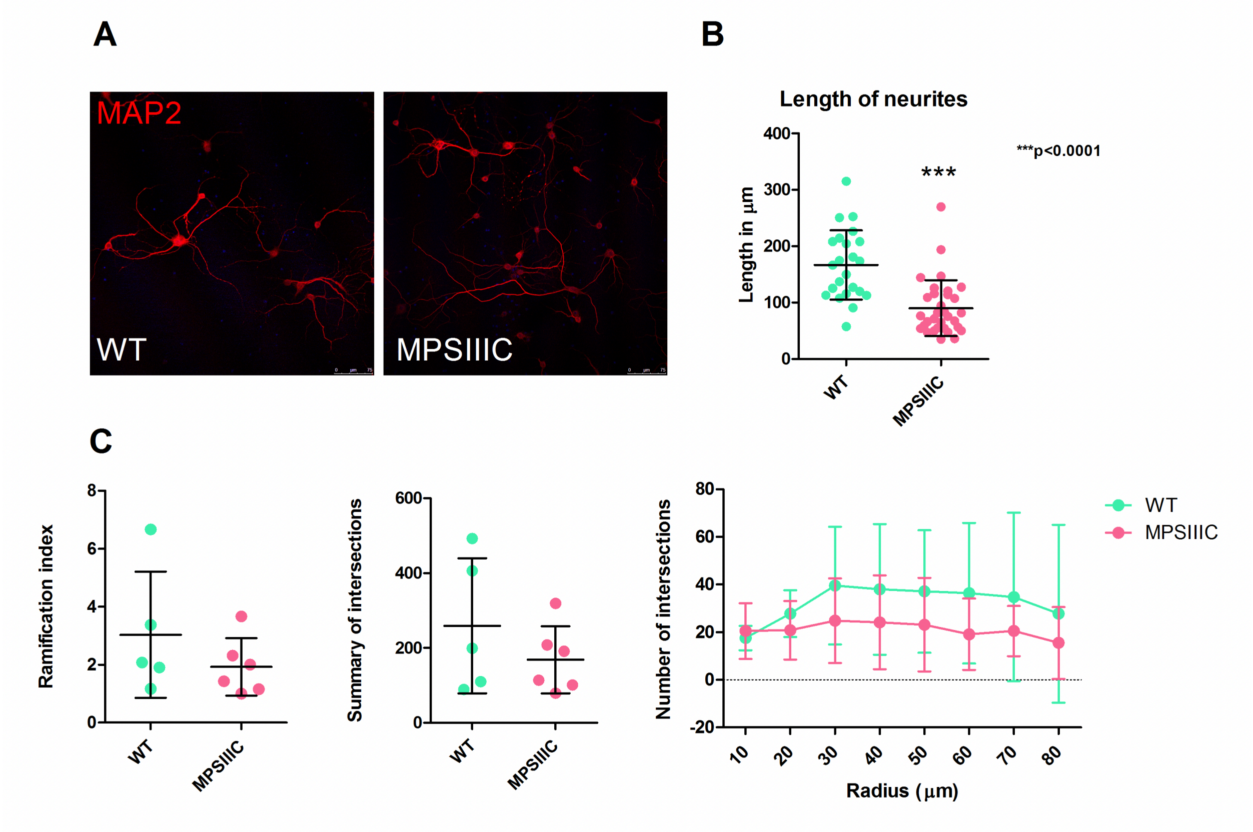

Figure S1: **Cultured hippocampal MPSIIIC neurons show smaller length of neurites as compared to the WT cells but similar ramification index and number of intersections in Sholl analysis.**

(A) Representative images of hippocampal cultured neurons stained with anti-MAP2 antibodies. **(B)** Quantification of length of neurites in hippocampal neuronal cultures. **(C)** Ramification index (left), summary of neurite intersections (middle) and number of neurite intersections at different distance from the cell soma (right) measured by Sholl analysis. Graphs show data from 3 different cultures with at least 10 cells analysed per culture. P-values were calculated by t-tests (length of neurites, ramification index, summary of neurite intersections) or two-way ANOVA (number of intersections).

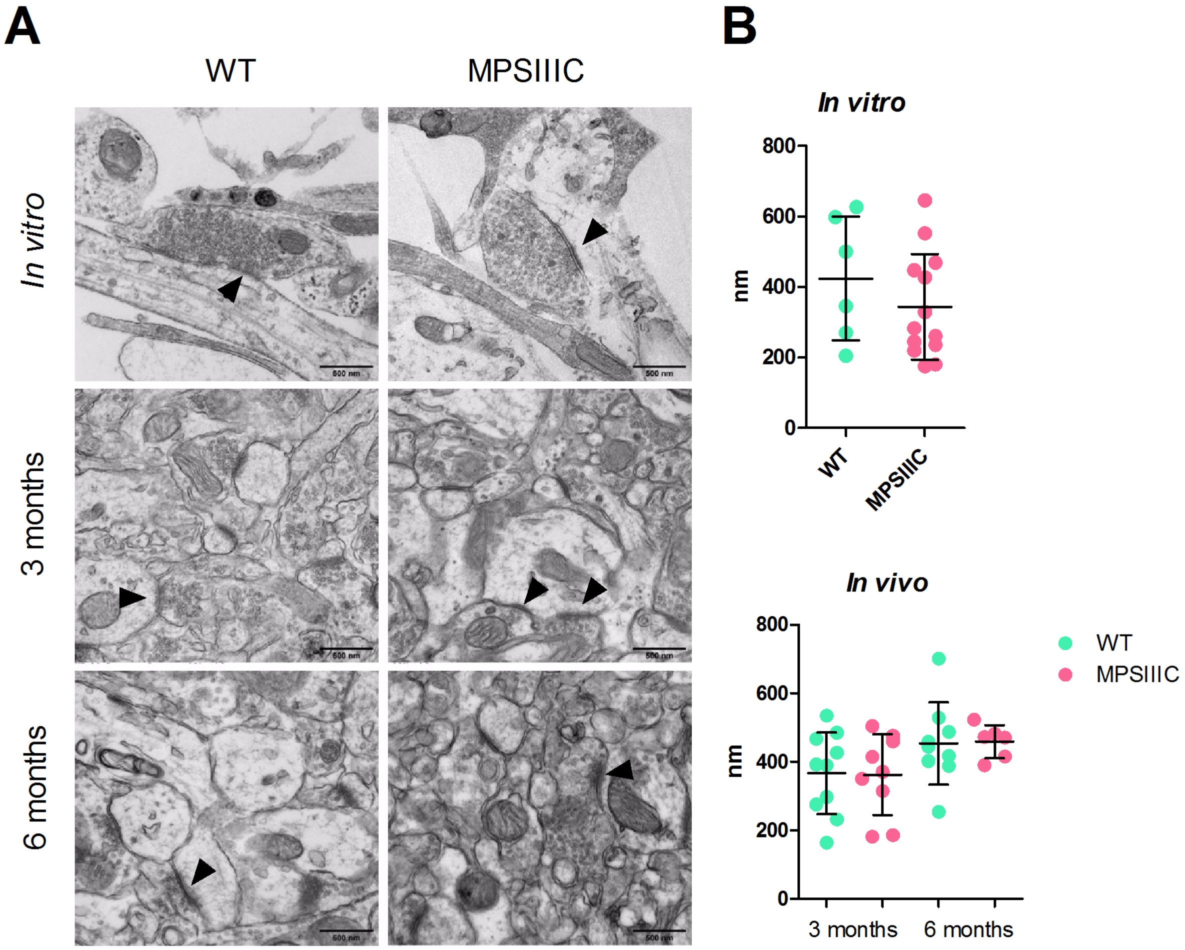

Figure S2: Lengths of PSD in symmetrical inhibitory synapses are similar between MPSIIIC and WT neurons in vivo and in vitro.

(A) Representative electron micrographs of inhibitory symmetric PSD (arrowheads) in cultured neurons and in the CA1 region of the hippocampus from 3 and 6-month-old mice.
(B) Quantification of length of PSD in vitro and in vivo. Graphs show individual values, means and SD from 3 independent sets of cultures or from 3 different mice per genotype. Three to five images were analysed per experiment. Size bars equal 500 nm.

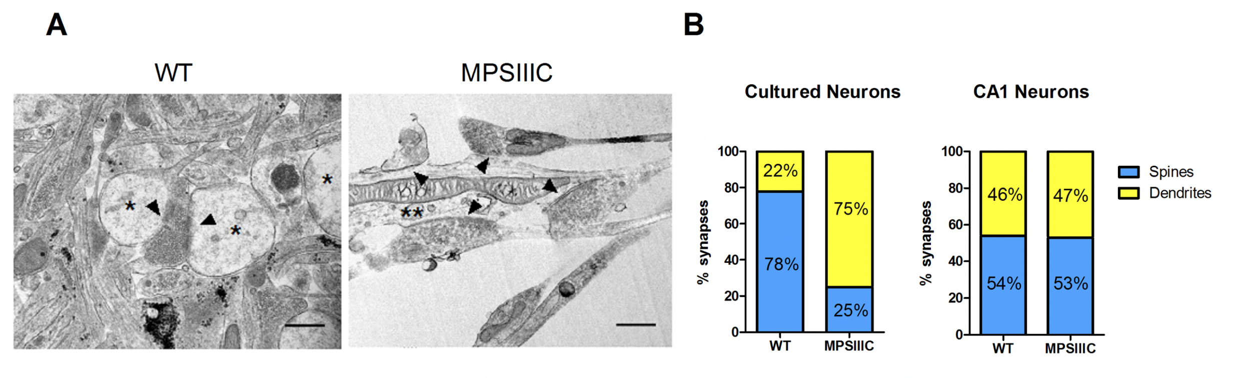

Figure S3: (A) Representative TEM images of synapses in WT and MPSIIIC cultured hippocampal neurons at DIV 21 showing alteration in the distribution of synapses. In MPSIIIC cultured neurons, there is a shift from axospinous to an axodendritic pattern. Spine (*), synaptic cleft (arrowheads) and dendrite (**). Scale bar equals 500 nm.

(B) Quantification of axospinous and axodendritic synaptic connections in cultured hippocampal neurons and in the pyramidal neurons in the CA1 region of the hippocampus of 6-month-old WT and MPSIIIC mice. Data show average values of 3 cultures or of 3 mice per genotype. At least 10 images with several synapses each, for each experiment, were analyzed.

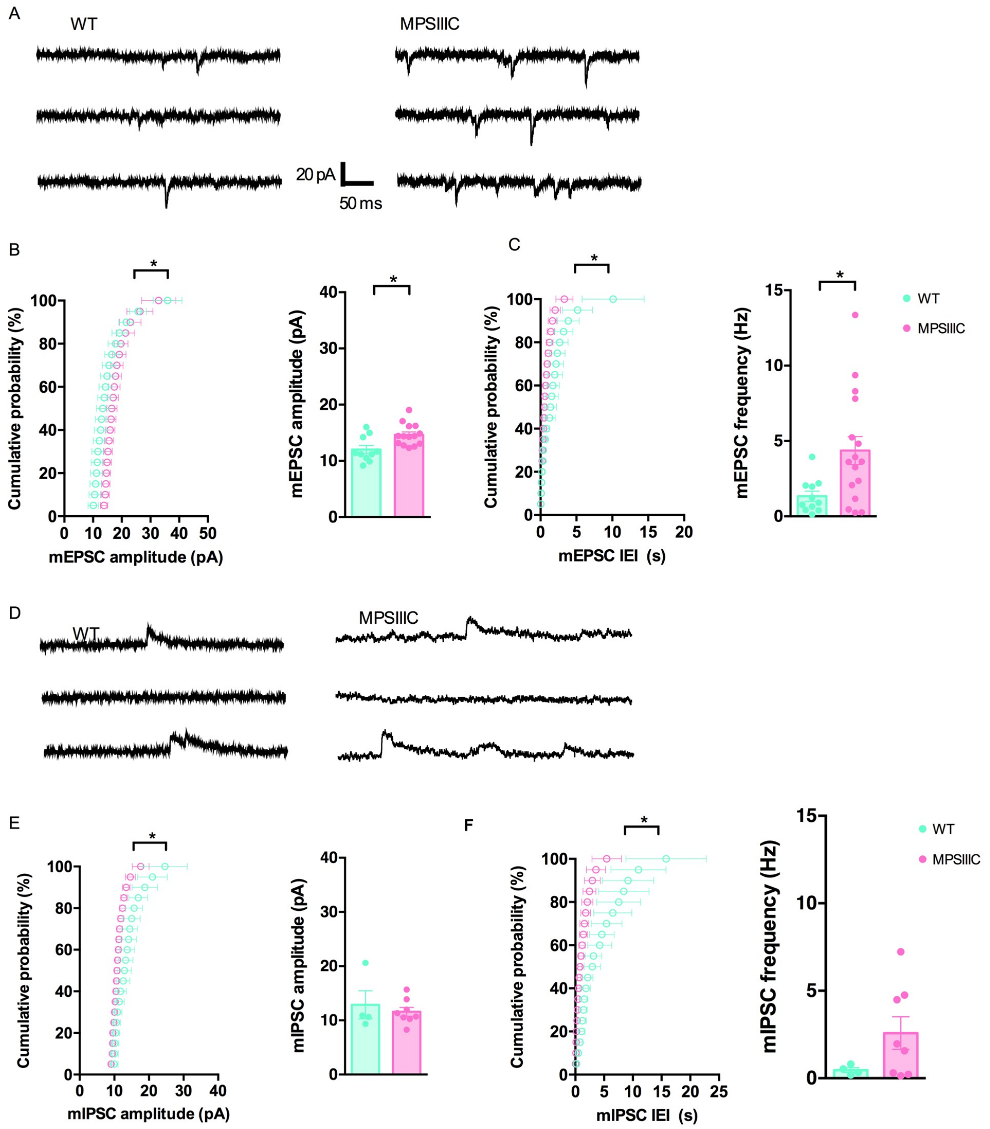

Figure S4: (A) Representative traces of mEPSCs in WT and MPSIIIC cultured hippocampal neurons at DIV19-22. Significant increase in the amplitude **(B)** and frequency **(C)** of mEPSCs in MPSIIIC neurons, as well as significant difference in distribution of amplitude and inter-event interval was observed as compared to WT controls **(D)** Representative traces, of mIPSCs in WT and MPSIIIC cultured hippocampal neurons. No significant change in mIPSC amplitude **(E)** and frequency **(F)** of mIPSCs was observed in MPSIIIC neurons as compared to WT controls. However, a significant difference in distribution of mIPSC amplitude and inter-event interval was observed between WT and MPSIIIC neurons. To detect significance of the difference between the means for all normally distributed data (mEPSC frequency) Student's t-test (* $p<0.05$) was used. For data not normally distributed (mEPSC amplitude, mIPSC frequency and amplitude) Mann-Whitney test was used (* $p<0.05$). Kolmogorov-Smirnov test was performed to detect significance of the difference in the distribution of not normally distributed events (* $p<0.05$).

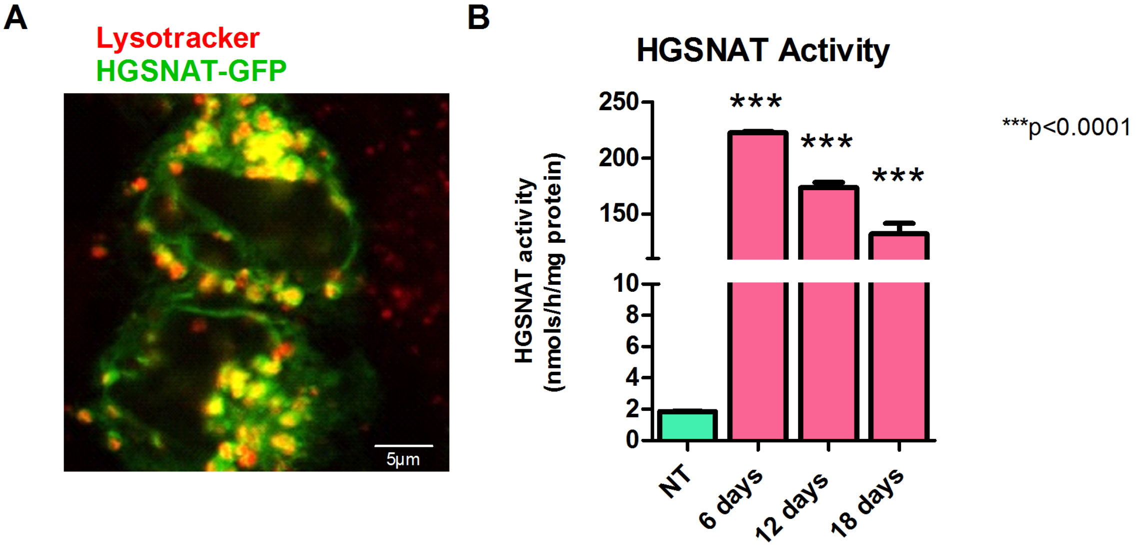

Figure S5: Transduction of HEK 293 cells with LV-HGSNAT-GFP results in expression of enzymatically active and correctly targeted HGSNAT-GFP fusion protein.

(A) Representative immunocytochemistry images of HEK 293 cells transduced with LV-HGSNAT-GFP. Colocalization of GFP puncta and Lysotracker red staining demonstrates that HGSNAT-GFP fusion protein is correctly targeted to the lysosomes. **(B)** HGSNAT activity measured in homogenates of HEK293 cells, transduced with LV-HGSNAT-GFP, is >100-fold increased as compared with the cells transduced with LV-GFP. Data show mean results and SD of 3 experiments. P-values were calculated by one-way ANOVA with Bonferroni post-test.

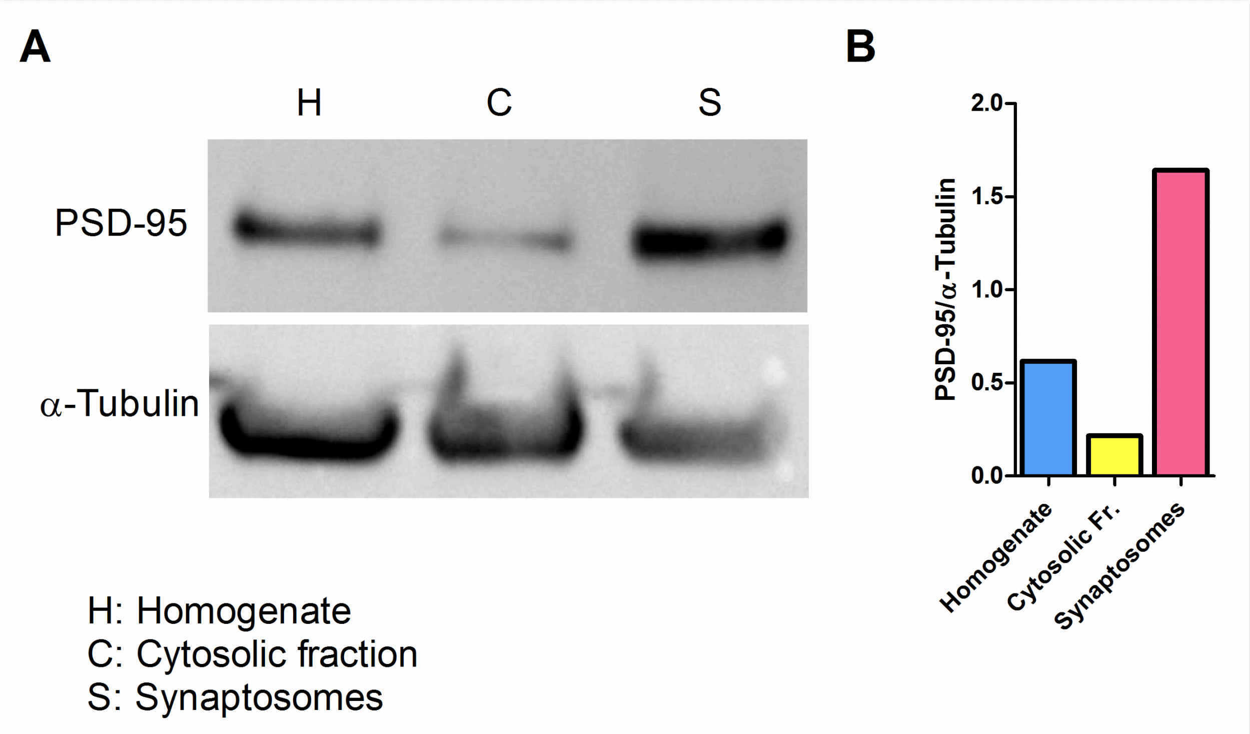

Figure S6: Synaptic protein marker PSD95 is ~3-fold enriched in the synaptosomal fraction as compared with a total brain homogenate.
(A) Western blots for PSD-95 in total brain homogenates, cytosolic fraction and synaptosomes showing PSD-95 enrichment in synaptosomes. **(B)** Quantification of the PSD-95 band size from **(A)**. Representative result for one of 6 purifications are shown.

Supplementary table S1: MPS patients and control subjects used in the study.

UMBN	GUID	Disorder	Cause of death	Age: years, days	Sex	Race	Clinical information	Neuropathologic findings
561	NDAR_INV YV182KRN	MPSI, Hurler Syndrome	Complications of disorder	6, 265	Female	Caucasian	Was oxygen dependent; had hydrocephalus, cardiomyopathy, chronic sinusitis, ear infections, blindness, hearing impairment, numerous pneumonias and hernias.	Neocortex with distended, "ballooned" neurons with mucin- and Alcian-blue positive material, also present throughout the central nervous system; occasional perivascular macrophages with similar material in the white matter; EM shows typical "zebra bodies" seen in mucopolysaccharidoses.
902	NDAR_INV TG497HU7	MPSII, Hunter Syndrome	Complications of disorder	42, 134	Male	Caucasian	Had multiple complications related to Hunter's syndrome, including tracheobronchial malacia, recurrent bronchitis and pneumonia, had multiple repairs of anterior abdominal wall hernia, was blind and somewhat deaf, had bilateral carpal tunnel release, history of mitral and aortic insufficiency, congestive heart failure.	Gliosis with axonal degeneration, optic nerves bilaterally, with neuronal loss and gliosis, lateral geniculate nucleus, old hemorrhagic cystic infarct, right occipital cortex and white matter, periventricular benign epidermal cyst, right occipital.
3617	NDAR_INVF P950EUM	MPSIIIA, Sanfilippo A Syndrome	Complications of disorder	12, 38	Female	Caucasian	N/A	Neurons throughout the brain have enlarged cell bodies with foamy cytoplasm, mild gliosis and status spongiosis in adjacent parenchyma,

								these neuronal changes are particularly severe in cerebral cortex, Purkinje cell layer of the cerebellum and substantia nigra. The choroid plexus epithelial cells are similarly affected with slightly enlarged and vacuolated cytoplasm. The centrum semi-ovale is mildly gliotic, its perivascular spaces dilated, fibrotic and contain glitter cells and occasional lymphocytes.
563	NDAR_INV RR063YHC	MPSIIIA, Sanfilippo A Syndrome	Complications of disorder	11, 101	Female	Caucasian	Two years before the death was attending school although having trouble walking and eating, by the time of death was non-verbal with deteriorating psychomotor skills, self-injurious behavior, had problems with sleep and agitation, suffered from mitral valve prolapse with myxomatous changes and mild regurgitation.	N/A
6194		MPSIIIC, Sanfilippo C Syndrome	Acute pneumonia as a consequence of disorder	20, 95	Male	African-American	Had a history of developmental delays, Nissen fundoplication and G-tube, asthma, seizures, sleep problems, agitation, used hearing aids.	The brain showed cerebral atrophy, mild hydrocephalus, neuronal enlargement with positive cytoplasmic PAS, Alcian blue and LFB, perivascular cuffing of foamy macrophages, white matter vacuolation.

5411	NDAR_INV AB442TCG	MPSIIID, Sanfilippo D Syndrome	Complications of disorder	24, 280	Female	Caucasian	One of two siblings suffering from Sanfilippo D, had a progressive neurologic decline with loss of vision, verbal expression, continence; had rare seizures in spite of anticonvulsive treatment and was wheelchair bound for the last year of life.	Generalized cerebral atrophy and neuronal storage disorder.
5424	NDAR_INV UC095YP2	MPSIIID, Sanfilippo D Syndrome	Complications of disorder	23, 149	Female	Caucasian	One of two siblings suffering from Sanfilippo D, had a progressive decline in hearing, verbal and visual abilities, had no specific cardiopulmonary symptomatology, no seizures, wheelchair bound for the last two years of life.	Generalized cerebral atrophy and neuronal storage disorder.
5967	NDAR_INV LZ650HFK	MPSIIIB, Sanfilippo B Syndrome	Complications of disorder	23, 58	Female	Caucasian	Had a history of progressive cognitive decline, hearing loss, recurrent otitis media status post PE tube insertions, concurrent epilepsy, dystonic spasms, oral dyskinesia, decline of motor skills, concurrent leukopenia, neutropenia and thrombocytopenia.	N/A
1878	NDAR_INV GM113DMK	MPSIIIA, Sanfilippo A Syndrome	Complications of disorder	10, 191	Female	Caucasian	Had a history of developmental delays, hearing loss, adenectomy, had no seizures since the one at birth.	Intraneuronal ganglioside storage of moderate severity; cerebral cortical atrophy with moderate neuronal loss, leptomeningeal fibrosis with mild communicating hydrocephalus.

662	NDAR_INV CK582GNX	Unaffected Control	Accident, multiple injuries	12, 356	Female	Caucasian		N/A
754	NDAR_INVJ V820CBR	Unaffected Control	Asthma	11, 201	Female	Native Hawaiian /Pacific Islander		N/A
1266	NDAR_INV CX672EJ2	Unaffected Control	Arteriosclerotic cardiovascular disease	42, 0	Male	Caucasian		N/A
4641	NDAR_INV NG087HR2	Unaffected Control	Acute asthma	24, 288	Female	African-American		N/A
5287	NDAR_INV UB832RTY	Unaffected Control	Multiple injuries	23, 195	Female	Caucasian		N/A
5813	NDAR_INV WA136XNT	Unaffected Control	Atherosclerotic cardiovascular disease	20, 362	Male	African-American		N/A
5977	NDAR_INV AX199AGW	Unaffected Control	Smoke inhalation	6, 248	Female	Caucasian		N/A

Supplementary table S2: Passive membrane properties of CA1 neurons in WT and MPSIIIC mice.

Characteristics	WT (P14-20)	MPSIIIC (P14-20)	WT (P45-60)	MPSIIIC (P45-60)
Resting membrane potential (mV)	-63.21±0.8	-60.10±0.32	-64.5±0.6	-62.2±0.7
Input resistance	142± 3.56	150±4.78	148±6.23	152±5.01
Action potential half width (ms) (in response to a 100 pA current injection of 500 ms duration)	0.90±0.01	0.89±0.07	0.94±0.05	0.96±0.08
Action potential threshold (mV)	-47.61±0.56	-48.22±0.37	-48.92±0.76	-47.65±0.32
Action potential firing frequency (Hz) (in response to a 100 pA current injection of 500 ms duration)	11.21±0.47	10.99±1.30	10.89±1.88	11.31±0.95

Supplementary table S3: List of reduced proteins in MPSIIIC synaptosomes of 3 and 6 month-old mice identified by LC-MS/MS

List of reduced proteins in synaptosomes from brains of 3 month-old mice												
#	Identified Proteins	Alternate ID	Molecular Weight	T-Test (p-value): (p < 0.05)	Quantitative Profile	KO S1	KO S2	KO S3	WT S1	WT S2	WT S3
5	Sodium/potassium-transporting ATPase subunit alpha OS=Mus musculus GN=Atp1a3 PE=1 SV=1	Atp1a3	113 kDa	0.013	KO Synap low, WT Synap high	27	30	37	46	50	50
6	Clathrin heavy chain 1 OS=Mus musculus GN=Cltc PE=1 SV=3	Cltc	192 kDa	0.0066	KO Synap low, WT Synap high	19	24	28	33	37	34
7	Excitatory amino acid transporter 2 OS=Mus musculus GN=Slc1a2 PE=1 SV=1	Slc1a2	62 kDa	0.024	KO Synap low, WT Synap high	9	9	13	13	13	14
8	Syntaxin-binding protein 1 OS=Mus musculus GN=Stxbp1 PE=1 SV=2	Stxbp1	68 kDa	0.0046	KO Synap low, WT Synap high	15	22	25	33	37	39
9	Sodium/potassium-transporting ATPase subunit alpha-2 OS=Mus musculus GN=Atp1a2 PE=1 SV=1	Atp1a2	112 kDa	0.049	KO Synap low, WT Synap high	12	15	18	23	27	22
10	Fructose-bisphosphate aldolase C OS=Mus musculus GN=Aldoc PE=1 SV=4	Aldoc	39 kDa	0.01	KO Synap low, WT Synap high	11	10	8	13	15	11
11	Fructose-bisphosphate aldolase A OS=Mus musculus GN=Aldoa PE=1 SV=2	Aldoa	39 kDa	0.035	KO Synap low, WT Synap high	16	9	16	18	24	18
12	Malate dehydrogenase, mitochondrial OS=Mus musculus GN=Mdh2 PE=1 SV=3	Mdh2	36 kDa	0.035	KO Synap low, WT Synap high	4	19	16	24	31	27
13	Dynamin-1 OS=Mus musculus GN=Dnm1 PE=1 SV=1	Dnm1	97 kDa	0.0012	KO Synap low, WT Synap high	16	16	16	28	24	27
14	Aconitate hydratase, mitochondrial OS=Mus musculus GN=Aco2 PE=1 SV=1	Aco2	85 kDa	0.023	KO Synap low, WT Synap high	0	17	17	24	28	27
15	Vesicle-fusing ATPase OS=Mus musculus GN=Nsf PE=1 SV=2	Nsf	83 kDa	0.04	KO Synap low, WT Synap high	12	12	13	16	22	17
16	Spectrin alpha chain, non-erythrocytic 1 OS=Mus musculus GN=Sptan1 PE=1 SV=1	Sptan1	285 kDa	0.047	KO Synap low, WT Synap high	1	10	17	17	31	27
17	Calcium/calmodulin-dependent protein kinase type II subunit alpha OS=Mus musculus GN=Camk2a PE=1 SV=2	Camk2a	54 kDa	0.0067	KO Synap low, WT Synap high	13	11	14	15	17	17
18	Neural cell adhesion molecule 1 OS=Mus musculus GN=Ncam1 PE=1 SV=3	Ncam1	119 kDa	0.0063	KO Synap low, WT Synap high	9	9	12	14	13	14
19	Spectrin beta chain, non-erythrocytic 1 OS=Mus musculus GN=Sptbn1 PE=1 SV=2	Sptbn1	274 kDa	0.0046	KO Synap low, WT Synap high	11	8	12	17	21	22
20	Hexokinase 1, isoform CRA_f OS=Mus musculus GN=Hk1 PE=1 SV=1	Hk1	102 kDa	0.024	KO Synap low, WT Synap high	2	11	12	14	15	18
21	60 kDa heat shock protein, mitochondrial OS=Mus musculus GN=Hspd1 PE=1 SV=1	Hspd1	61 kDa	0.0065	KO Synap low, WT Synap high	0	10	13	18	17	20
22	Serine/threonine-protein phosphatase 2A 65 kDa regulatory subunit A alpha isoform OS=Mus musculus GN=Ppp2r1a PE=1 SV=3	Ppp2r1a	65 kDa	0.008	KO Synap low, WT Synap high	8	3	8	9	9	9
23	Calcium-dependent secretion activator 1 OS=Mus musculus GN=Cadps PE=1 SV=3	Cadps	153 kDa	0.025	KO Synap low, WT Synap high	7	6	6	8	9	8
24	Stress-70 protein, mitochondrial OS=Mus musculus GN=Hspa9 PE=1 SV=3	Hspa9	73 kDa	0.021	KO Synap low, WT Synap high	0	7	7	13	16	17
25	Ankyrin-2 OS=Mus musculus GN=Ank2 PE=1 SV=2	Ank2	426 kDa	0.0054	KO Synap low, WT Synap high	7	8	9	14	20	16

26	Contactin-1 OS=Mus musculus GN=Cntn1 PE=1 SV=1	Cntn1	113 kDa	0.01	KO Synap low, WT Synap high	4	4	7	9	11	9
27	Synaptosomal-associated protein 25 OS=Mus musculus GN=Snap25 PE=1 SV=1	Snap25	23 kDa	0.023	KO Synap low, WT Synap high	3	6	8	8	10	10
28	Cytochrome c, somatic OS=Mus musculus GN=Cycs PE=1 SV=2	Cycs	12 kDa	0.0096	KO Synap low, WT Synap high	0	4	5	7	8	7
29	Calcium-binding mitochondrial carrier protein Aralar1 OS=Mus musculus GN=Slc25a12 PE=1 SV=1	Slc25a12	75 kDa	0.025	KO Synap low, WT Synap high	0	5	6	11	9	12
30	Cytochrome b-c1 complex subunit 1, mitochondrial OS=Mus musculus GN=Uqcrc1 PE=1 SV=2	Uqcrc1	53 kDa	0.02	KO Synap low, WT Synap high	0	5	7	8	7	8
31	Pyruvate dehydrogenase E1 component subunit beta, mitochondrial OS=Mus musculus GN=Pdhb PE=1 SV=1	Pdhb	39 kDa	0.045	KO Synap low, WT Synap high	0	6	9	9	10	7
32	AP-2 complex subunit alpha-2 OS=Mus musculus GN=Ap2a2 PE=1 SV=2	Ap2a2	104 kDa	0.0064	KO Synap low, WT Synap high	3	2	3	8	5	9
33	Beta-soluble NSF attachment protein OS=Mus musculus GN=Napb PE=1 SV=2	Napb	34 kDa	0.0011	KO Synap low, WT Synap high	2	3	4	5	6	5
34	Dynamin-like 120 kDa protein, mitochondrial OS=Mus musculus GN=Opa1 PE=1 SV=1	Opa1	111 kDa	0.02	KO Synap low, WT Synap high	0	7	8	11	13	11
35	2',3'-cyclic-nucleotide 3'-phosphodiesterase OS=Mus musculus GN=Cnp PE=1 SV=3	Cnp	47 kDa	0.017	KO Synap low, WT Synap high	1	3	4	6	7	10
36	Isocitrate dehydrogenase [NAD] subunit gamma 1, mitochondrial OS=Mus musculus GN=Idh3g PE=1 SV=1	Idh3g	43 kDa	0.026	KO Synap low, WT Synap high	0	2	1	5	6	6
37	Neurofilament 3, medium OS=Mus musculus GN=Nefm PE=1 SV=1	Nefm	96 kDa	0.027	KO Synap low, WT Synap high	4	1	4	4	8	8
38	ATP synthase subunit O, mitochondrial OS=Mus musculus GN=Atp5o PE=1 SV=1	Atp5o	23 kDa	0.031	KO Synap low, WT Synap high	0	6	5	8	8	9
39	ProSAAS OS=Mus musculus GN=Pcsk1n PE=1 SV=2	Pcsk1n	27 kDa	0.018	KO Synap low, WT Synap high	2	2	3	5	3	5
40	Dihydrolipoyllysine-residue acetyltransferase component of pyruvate dehydrogenase complex, mitochondrial OS=Mus musculus GN=Dlat PE=1 SV=2	Dlat	68 kDa	0.024	KO Synap low, WT Synap high	0	4	5	8	7	8
41	NADH dehydrogenase [ubiquinone] 1 alpha subcomplex subunit 9, mitochondrial OS=Mus musculus GN=Ndufa9 PE=1 SV=1	Ndufa9	42 kDa	0.0052	KO Synap low, WT Synap high	0	3	4	7	8	9
42	AP-2 complex subunit beta OS=Mus musculus GN=Ap2b1 PE=1 SV=1	Ap2b1	105 kDa	0.045	KO Synap low, WT Synap high	3	0	6	10	8	7
43	Fumarate hydratase, mitochondrial OS=Mus musculus GN=Fh PE=1 SV=3	Fh	54 kDa	0.027	KO Synap low, WT Synap high	1	3	5	7	8	9
44	Septin-7 OS=Mus musculus GN=Sept7 PE=1 SV=2	7-Sep	51 kDa	0.024	KO Synap low, WT Synap high	4	4	6	8	8	7
45	Cytochrome b-c1 complex subunit 2, mitochondrial OS=Mus musculus GN=Uqcrc2 PE=1 SV=1	Uqcrc2	48 kDa	0.0052	KO Synap low, WT Synap high	0	3	4	7	8	6
46	N(G),N(G)-dimethylarginine dimethylaminohydrolase 1 OS=Mus musculus GN=Ddah1 PE=1 SV=3	Ddah1	31 kDa	0.024	KO Synap low, WT Synap high	2	2	2	3	3	3
47	Peroxiredoxin-5, mitochondrial OS=Mus musculus GN=Prdx5 PE=1 SV=2	Prdx5	22 kDa	0.029	KO Synap low, WT Synap high	0	4	1	4	5	6
48	Succinate--CoA ligase [ADP-forming] subunit beta, mitochondrial OS=Mus musculus GN=Sucla2 PE=1 SV=2	Sucla2	50 kDa	0.021	KO Synap low, WT Synap high	0	2	6	8	9	12
49	Septin-11 OS=Mus musculus GN=Sept11 PE=1 SV=1	11-Sep	49 kDa	0.019	KO Synap low, WT Synap high	2	4	4	6	8	7
50	Reticulon-4 OS=Mus musculus GN=Rtn4 PE=1 SV=2	Rtn4	127 kDa	0.008	KO Synap low, WT Synap high	2	2	2	5	5	5
51	AP-2 complex subunit mu OS=Mus musculus GN=Ap2m1 PE=1 SV=1	Ap2m1	50 kDa	0.0062	KO Synap low, WT Synap high	3	0	2	4	4	4
52	Inositol 1,4,5-trisphosphate receptor type 1 OS=Mus musculus GN=Itpr1 PE=1 SV=2	Itpr1	313 kDa	0.019	KO Synap low, WT Synap high	2	3	4	7	11	10
53	Isocitrate dehydrogenase [NAD] subunit, mitochondrial OS=Mus musculus GN=Idh3b PE=1 SV=1	Idh3b	42 kDa	0.0094	KO Synap low, WT Synap high	0	4	3	9	8	11

54	Prohibitin OS=Mus musculus GN=Phb PE=1 SV=1	Phb	30 kDa	0.041	KO Synap low, WT Synap high	0	2	7	8	9	8
55	NADH dehydrogenase [ubiquinone] flavoprotein 1, mitochondrial OS=Mus musculus GN=Ndufv1 PE=1 SV=1	Ndufv1	50 kDa	0.024	KO Synap low, WT Synap high	0	3	4	6	10	9
56	Glutamate decarboxylase 2 OS=Mus musculus GN=Gad2 PE=1 SV=1	Gad2	65 kDa	0.00039	KO Synap low, WT Synap high	3	1	2	5	6	4
57	AP-2 complex subunit alpha-1 OS=Mus musculus GN=Ap2a1 PE=1 SV=1	Ap2a1	108 kDa	0.039	KO Synap low, WT Synap high	5	2	3	2	4	1
58	NADH dehydrogenase [ubiquinone] 1 alpha subcomplex subunit 10, mitochondrial OS=Mus musculus GN=Ndufa10 PE=1 SV=1	Ndufa10	41 kDa	0.02	KO Synap low, WT Synap high	0	3	5	6	7	9
59	Acyl-CoA-binding protein OS=Mus musculus GN=Dbi PE=1 SV=2	Dbi	10 kDa	0.011	KO Synap low, WT Synap high	0	0	2	2	2	2
60	V-type proton ATPase subunit E 1 OS=Mus musculus GN=Atp6v1e1 PE=1 SV=2	Atp6v1e 1	26 kDa	0.041	KO Synap low, WT Synap high	3	0	2	2	4	5
61	Tropomyosin 1, alpha, isoform CRA_i OS=Mus musculus GN=Tpm1 PE=1 SV=1	Tpm1	28 kDa	0.039	KO Synap low, WT Synap high	0	1	1	4	3	5
62	Plasma membrane calcium-transporting ATPase 1 OS=Mus musculus GN=Atp2b1 PE=1 SV=1	Atp2b1	135 kDa	0.013	KO Synap low, WT Synap high	2	2	2	6	7	4
63	Alpha actinin 1a OS=Mus musculus GN=Actn1 PE=1 SV=1	Actn1	103 kDa	0.0079	KO Synap low, WT Synap high	2	1	2	4	5	5
64	Cytochrome c oxidase subunit 5A, mitochondrial OS=Mus musculus GN=Cox5a PE=1 SV=2	Cox5a	16 kDa	0.023	KO Synap low, WT Synap high	1	2	1	3	4	5
65	Mitochondrial import receptor subunit TOM70 OS=Mus musculus GN=Tomm70 PE=1 SV=2	Tomm7 0	68 kDa	0.028	KO Synap low, WT Synap high	0	2	3	7	7	2
66	Dihydrolipoyllysine-residue succinyltransferase component of 2-oxoglutarate dehydrogenase complex, mitochondrial OS=Mus musculus GN=Dlst PE=1 SV=1	Dlst	49 kDa	0.01	KO Synap low, WT Synap high	0	2	2	3	5	5
67	Calretinin OS=Mus musculus GN=Calb2 PE=1 SV=3	Calb2	31 kDa	0.019	KO Synap low, WT Synap high	0	0	0	3	3	2
68	Aldehyde dehydrogenase, mitochondrial OS=Mus musculus GN=Aldh2 PE=1 SV=1	Aldh2	51 kDa	0.028	KO Synap low, WT Synap high	0	3	4	7	7	6
69	Catenin alpha-2 OS=Mus musculus GN=Ctnna2 PE=1 SV=3	Ctnna2	105 kDa	0.047	KO Synap low, WT Synap high	0	3	4	6	9	4
70	Synaptophysin OS=Mus musculus GN=Syp PE=1 SV=2	Syp	34 kDa	0.022	KO Synap low, WT Synap high	0	1	1	2	3	3
71	LETM1 and EF-hand domain-containing protein 1, mitochondrial OS=Mus musculus GN=Letm1 PE=1 SV=1	Letm1	83 kDa	0.031	KO Synap low, WT Synap high	0	3	1	4	7	7
72	Anion exchange protein OS=Mus musculus GN=Slc4a4 PE=1 SV=1	Slc4a4	123 kDa	0.0065	KO Synap low, WT Synap high	0	0	0	4	6	3
73	Ras-related protein Rab-7a OS=Mus musculus GN=Rab7a PE=1 SV=2	Rab7a	23 kDa	0.018	KO Synap low, WT Synap high	0	2	1	2	3	3
74	Alpha-internexin OS=Mus musculus GN=Ina PE=1 SV=3	Ina	55 kDa	0.0092	KO Synap low, WT Synap high	0	0	1	4	6	6
75	Septin-5 OS=Mus musculus GN=Sept5 PE=1 SV=2	5-Sep	43 kDa	0.044	KO Synap low, WT Synap high	0	0	3	3	2	3
76	AP-1 complex subunit beta-1 OS=Mus musculus GN=Ap1b1 PE=1 SV=2	Ap1b1	104 kDa	0.044	KO Synap low, WT Synap high	2	2	2	2	3	2
77	Disks large homolog 4 OS=Mus musculus GN=Dlg4 PE=1 SV=1	Dlg4	80 kDa	0.021	KO Synap low, WT Synap high	0	1	2	4	5	7
78	Elongation factor 1-beta OS=Mus musculus GN=Eef1b PE=1 SV=5	Eef1b	25 kDa	0.016	KO Synap low, WT Synap high	1	0	1	1	1	1
79	NADH dehydrogenase [ubiquinone] iron-sulfur protein 7, mitochondrial OS=Mus musculus GN=Ndufs7 PE=1 SV=1	Ndufs7	25 kDa	0.024	KO Synap low, WT Synap high	0	2	1	3	2	3
80	Cytochrome b-c1 complex subunit 6, mitochondrial OS=Mus musculus GN=Uqcrh PE=1 SV=2	Uqcrh	10 kDa	0.018	KO Synap low, WT Synap high	0	1	1	2	2	2
81	Serine/threonine-protein phosphatase 2A catalytic subunit alpha isoform OS=Mus musculus GN=Ppp2ca PE=1 SV=1	Ppp2ca	36 kDa	0.016	KO Synap low, WT Synap high	1	0	1	2	2	2

82	NADH dehydrogenase [ubiquinone] 1 alpha subcomplex subunit 8 OS=Mus musculus GN=Ndufa8 PE=1 SV=3	Ndufa8	20 kDa	0.016	KO Synap low, WT Synap high	0	0	1	3	4	2
83	Protein disulfide-isomerase A6 OS=Mus musculus GN=Pdia6 PE=1 SV=1	Pdia6	49 kDa	0.0075	KO Synap low, WT Synap high	1	1	1	1	1	1
84	Actin-related protein 3 OS=Mus musculus GN=Actr3 PE=1 SV=3	Actr3	47 kDa	0.0021	KO Synap low, WT Synap high	0	1	0	4	3	2
85	Neuroligin-2 OS=Mus musculus GN=Nlgn2 PE=1 SV=2	Nlgn2	91 kDa	0.018	KO Synap low, WT Synap high	0	1	1	3	4	5
86	Dynamin-3 OS=Mus musculus GN=Dnm3 PE=1 SV=1	Dnm3	97 kDa	0.018	KO Synap low, WT Synap high	0	1	0	4	6	4
87	Rho-related GTP-binding protein RhoB OS=Mus musculus GN=Rhob PE=1 SV=1	Rhob	22 kDa	0.035	KO Synap low, WT Synap high	0	1	2	3	3	2
88	Importin subunit beta-1 OS=Mus musculus GN=Kpnb1 PE=1 SV=2	Kpnb1	97 kDa	0.00056	KO Synap low, WT Synap high	0	0	0	2	4	3
89	Methylmalonate-semialdehyde dehydrogenase [acylating], mitochondrial OS=Mus musculus GN=Aldh6a1 PE=1 SV=1	Aldh6a1	58 kDa	0.023	KO Synap low, WT Synap high	0	1	1	5	2	5
90	Voltage-dependent anion-selective channel protein 1 OS=Mus musculus GN=Vdac1 PE=1 SV=3	Vdac1	32 kDa	0.04	KO Synap low, WT Synap high	0	0	0	1	3	4
91	NADH dehydrogenase [ubiquinone] iron-sulfur protein 5 OS=Mus musculus GN=Ndufs5 PE=1 SV=3	Ndufs5	13 kDa	0.0048	KO Synap low, WT Synap high	0	0	1	2	3	3
92	Serine/threonine-protein phosphatase PP1-gamma catalytic subunit OS=Mus musculus GN=Ppp1cc PE=1 SV=1	Ppp1cc	37 kDa	0.047	KO Synap low, WT Synap high	1	1	0	2	3	4
93	Anion exchange protein OS=Mus musculus GN=Slc4a10 PE=1 SV=1	Slc4a10	125 kDa	0.018	KO Synap low, WT Synap high	0	1	0	6	3	4
94	Cytochrome b-c1 complex subunit 10 OS=Mus musculus GN=Uqcr11 PE=3 SV=1	Uqcr11	7 kDa	0.0061	KO Synap low, WT Synap high	0	2	0	2	2	2
95	Cytosolic non-specific dipeptidase OS=Mus musculus GN=Cndp2 PE=1 SV=1	Cndp2	53 kDa	0.013	KO Synap low, WT Synap high	1	1	0	1	2	3
96	V-type proton ATPase subunit G 2 OS=Mus musculus GN=Atp6v1g2 PE=1 SV=1	Atp6v1g2	14 kDa	0.013	KO Synap low, WT Synap high	0	1	1	2	1	2
97	F-actin-capping protein subunit alpha-2 OS=Mus musculus GN=Capza2 PE=1 SV=3	Capza2	33 kDa	0.0078	KO Synap low, WT Synap high	0	0	1	1	2	1
98	Superoxide dismutase [Mn], mitochondrial OS=Mus musculus GN=Sod2 PE=1 SV=3	Sod2	25 kDa	0.029	KO Synap low, WT Synap high	0	0	2	3	2	4
99	Electron transfer flavoprotein subunit beta OS=Mus musculus GN=Etfb PE=1 SV=3	Etfb	28 kDa	0.016	KO Synap low, WT Synap high	0	1	2	3	2	3
100	Casein kinase II subunit alpha OS=Mus musculus GN=Csnk2a1 PE=1 SV=2	Csnk2a1	45 kDa	0.016	KO Synap low, WT Synap high	0	0	0	2	3	1
101	MAGUK p55 subfamily member 2 OS=Mus musculus GN=Mpp2 PE=1 SV=1	Mpp2	62 kDa	0.016	KO Synap low, WT Synap high	0	0	2	3	2	3
102	Glycerol-3-phosphate dehydrogenase, mitochondrial OS=Mus musculus GN=Gpd2 PE=1 SV=2	Gpd2	81 kDa	0.033	KO Synap low, WT Synap high	0	0	1	3	4	2
103	Type I inositol 3,4-bisphosphate 4-phosphatase OS=Mus musculus GN=Inpp4a PE=1 SV=2	Inpp4a	105 kDa	0.0075	KO Synap low, WT Synap high	1	1	1	3	2	2
104	Glia maturation factor beta OS=Mus musculus GN=Gmfb PE=1 SV=3	Gmfb	17 kDa	0.025	KO Synap low, WT Synap high	0	0	0	2	1	2
105	Homer protein homolog 1 OS=Mus musculus GN=Homer1 PE=1 SV=1	Homer1	22 kDa	0.0065	KO Synap low, WT Synap high	0	0	0	1	2	2
106	NADH dehydrogenase [ubiquinone] 1 beta subcomplex subunit 10 (Fragment) OS=Mus musculus GN=Ndufb10 PE=1 SV=1	Ndufb10	18 kDa	0.0022	KO Synap low, WT Synap high	0	1	1	1	2	2
107	NADH dehydrogenase [ubiquinone] iron-sulfur protein 2, mitochondrial OS=Mus musculus GN=Ndufs2 PE=1 SV=1	Ndufs2	53 kDa	0.0078	KO Synap low, WT Synap high	0	0	1	2	2	2
108	Glutamate receptor 2 OS=Mus musculus GN=Gria2 PE=1 SV=1	Gria2	87 kDa	0.024	KO Synap low, WT Synap high	0	1	1	2	3	2
109	Metaxin-2 OS=Mus musculus GN=Mtx2 PE=1 SV=1	Mtx2	30 kDa	0.0031	KO Synap low, WT Synap high	0	1	0	3	3	3

110	Complement component 1 Q subcomponent-binding protein, mitochondrial OS=Mus musculus GN=C1qbp PE=1 SV=1	C1qbp	31 kDa	0.0065	KO Synap low, WT Synap high	0	0	0	2	2	2
111	Mitochondrial import receptor subunit TOM22 homolog OS=Mus musculus GN=Tomm22 PE=1 SV=3	Tomm22	16 kDa	0.0023	KO Synap low, WT Synap high	0	0	0	1	2	1
112	Basigin OS=Mus musculus GN=Bsg PE=1 SV=2	Bsg	42 kDa	0.0075	KO Synap low, WT Synap high	0	1	0	2	2	2
113	Arf-GAP with GTPase, ANK repeat and PH domain-containing protein 2 OS=Mus musculus GN=Agap2 PE=1 SV=1	Agap2	125 kDa	0.026	KO Synap low, WT Synap high	0	0	0	1	2	2
114	Dynamin-1 OS=Mus musculus GN=Dnm1 PE=1 SV=2	Dnm1	98 kDa	0.016	KO Synap low, WT Synap high	0	0	1	1	1	1
115	MCG10748, isoform CRA_b OS=Mus musculus GN=Rap1a PE=1 SV=1	Rap1a	13 kDa	0.024	KO Synap low, WT Synap high	0	1	1	2	3	2
116	Microtubule-associated protein RP/EB family member 3 OS=Mus musculus GN=Mapre3 PE=1 SV=1	Mapre3	30 kDa	0.0022	KO Synap low, WT Synap high	0	0	0	2	1	2
117	Serine/threonine-protein phosphatase 2A 56 kDa regulatory subunit epsilon isoform OS=Mus musculus GN=Ppp2r5e PE=1 SV=3	Ppp2r5e	55 kDa	0.0075	KO Synap low, WT Synap high	0	0	1	1	1	1
118	V-type proton ATPase subunit D OS=Mus musculus GN=Atp6v1d PE=1 SV=1	Atp6v1d	28 kDa	0.0022	KO Synap low, WT Synap high	0	0	0	2	1	1
119	Ras-related protein Rab-3C OS=Mus musculus GN=Rab3c PE=1 SV=1	Rab3c	26 kDa	0.034	KO Synap low, WT Synap high	0	1	0	1	2	2
120	Protein tweety homolog OS=Mus musculus GN=Ttyh1 PE=1 SV=1	Ttyh1	39 kDa	0.016	KO Synap low, WT Synap high	0	1	1	1	1	1
121	ADP-ribosylation factor-like protein 3 OS=Mus musculus GN=Arl3 PE=1 SV=1	Arl3	20 kDa	0.0075	KO Synap low, WT Synap high	0	0	0	1	1	1
122	Disks large homolog 2 OS=Mus musculus GN=Dlg2 PE=1 SV=2	Dlg2	95 kDa	0.0065	KO Synap low, WT Synap high	0	0	0	3	1	2
123	Voltage-dependent calcium channel subunit alpha-2/delta-1 OS=Mus musculus GN=Cacna2d1 PE=1 SV=1	Cacna2d1	125 kDa	0.013	KO Synap low, WT Synap high	0	0	1	2	3	2
124	Histone H1.0 OS=Mus musculus GN=H1f0 PE=2 SV=4	H1f0	21 kDa	0.0013	KO Synap low, WT Synap high	0	0	0	2	1	2
125	ERC protein 2 OS=Mus musculus GN=Erc2 PE=1 SV=2	Erc2	111 kDa	0.016	KO Synap low, WT Synap high	0	0	0	1	2	1
126	CB1 cannabinoid receptor-interacting protein 1 OS=Mus musculus GN=Cnrip1 PE=1 SV=1	Cnrip1	19 kDa	0.0022	KO Synap low, WT Synap high	0	0	0	1	2	2
127	Disks large-associated protein 2 OS=Mus musculus GN=Dlgap2 PE=1 SV=2	Dlgap2	119 kDa	0.016	KO Synap low, WT Synap high	0	0	0	1	2	1
128	Zinc transporter 9 OS=Mus musculus GN=Slc30a9 PE=1 SV=2	Slc30a9	63 kDa	0.016	KO Synap low, WT Synap high	0	0	0	1	2	1
129	Neuronal pentraxin-1 OS=Mus musculus GN=Nptx1 PE=1 SV=1	Nptx1	47 kDa	0.016	KO Synap low, WT Synap high	0	0	0	1	1	1
130	Syntaxin-binding protein 5 OS=Mus musculus GN=Stxbp5 PE=1 SV=1	Stxbp5	124 kDa	0.0075	KO Synap low, WT Synap high	0	0	0	1	2	2
131	3-ketoacyl-CoA thiolase, mitochondrial OS=Mus musculus GN=Acaa2 PE=1 SV=3	Acaa2	42 kDa	0.0075	KO Synap low, WT Synap high	0	0	0	2	2	1
132	Atlastin-2 OS=Mus musculus GN=Atl2 PE=1 SV=1	Atl2	66 kDa	0.0075	KO Synap low, WT Synap high	0	0	0	1	1	1
133	WD repeat-containing protein 7 OS=Mus musculus GN=Wdr7 PE=1 SV=3	Wdr7	163 kDa	0.016	KO Synap low, WT Synap high	0	0	0	1	2	1
134	Tetratricopeptide repeat protein 7B OS=Mus musculus GN=Ttc7b PE=1 SV=1	Ttc7b	94 kDa	0.016	KO Synap low, WT Synap high	0	0	0	2	1	1
135	Glutamate receptor 4 OS=Mus musculus GN=Gria4 PE=1 SV=1	Gria4	101 kDa	0.016	KO Synap low, WT Synap high	0	0	0	1	1	1
136	NADP-dependent malic enzyme, mitochondrial OS=Mus musculus GN=Me3 PE=1 SV=2	Me3	67 kDa	0.016	KO Synap low, WT Synap high	0	0	0	1	1	1
137	Microsomal glutathione S-transferase 3 OS=Mus musculus GN=Mgst3 PE=1 SV=1	Mgst3	17 kDa	0.016	KO Synap low, WT Synap high	0	0	0	1	1	1

List of reduced proteins in MPSIIIC synaptosomes of 6 month-old mice												
#	Identified Proteins	Alternate ID	Molecular Weight	T-Test (p-value): (p < 0.05)	Quantitative Profile	KO S4	KO S5	KO S6	WT S4	WT S5	WT S6
435	Sodium/potassium-transporting ATPase subunit alpha OS=Mus musculus GN=Atp1a3 PE=1 SV=1	Atp1a3	113 kDa	0.0015	KO Synap low, WT Synap high	46	52	57	54	59	62
436	Synapsin-1 OS=Mus musculus GN=Syn1 PE=1 SV=2	Syn1	74 kDa	< 0.00010	KO Synap low, WT Synap high	24	24	21	39	39	40
437	Aconitate hydratase, mitochondrial OS=Mus musculus GN=Aco2 PE=1 SV=1	Aco2	85 kDa	< 0.00010	KO Synap low, WT Synap high	13	9	13	64	59	61
438	Syntaxin-binding protein 1 OS=Mus musculus GN=Stxbp1 PE=1 SV=2	Stxbp1	68 kDa	0.0001	KO Synap low, WT Synap high	23	20	24	41	47	46
439	Dynamin-1 OS=Mus musculus GN=Dnm1 PE=1 SV=1	Dnm1	97 kDa	0.0046	KO Synap low, WT Synap high	43	41	33	53	53	54
440	Clathrin heavy chain 1 OS=Mus musculus GN=Cltc PE=1 SV=3	Cltc	192 kDa	0.0013	KO Synap low, WT Synap high	27	31	21	41	50	50
441	Fructose-bisphosphate aldolase A OS=Mus musculus GN=Aldoa PE=1 SV=2	Aldoa	39 kDa	0.00075	KO Synap low, WT Synap high	23	20	24	25	31	31
442	Brain acid soluble protein 1 OS=Mus musculus GN=Basp1 PE=1 SV=3	Basp1	22 kDa	< 0.00010	KO Synap low, WT Synap high	13	12	14	28	28	35
443	ATP synthase subunit beta, mitochondrial OS=Mus musculus GN=Atp5b PE=1 SV=2	Atp5b	56 kDa	< 0.00010	KO Synap low, WT Synap high	3	3	4	20	22	25
444	Microtubule-associated protein 6 OS=Mus musculus GN=Map6 PE=1 SV=2	Map6	96 kDa	0.00026	KO Synap low, WT Synap high	24	19	19	27	24	34
445	Vesicle-fusing ATPase OS=Mus musculus GN=Nsf PE=1 SV=2	Nsf	83 kDa	0.00048	KO Synap low, WT Synap high	28	26	27	29	30	31
446	Myelin basic protein (Fragment) OS=Mus musculus GN=Mbp PE=1 SV=1	Mbp	21 kDa	0.05	KO Synap low, WT Synap high	20	0	0	18	21	23
447	Glutamate dehydrogenase 1, mitochondrial OS=Mus musculus GN=Glud1 PE=1 SV=1	Glud1	61 kDa	< 0.00010	KO Synap low, WT Synap high	3	3	2	29	33	35
448	Excitatory amino acid transporter 2 OS=Mus musculus GN=Slc1a2 PE=1 SV=1	Slc1a2	62 kDa	0.0024	KO Synap low, WT Synap high	12	10	14	17	19	20
449	Hexokinase 1, isoform CRA_f OS=Mus musculus GN=Hk1 PE=1 SV=1	Hk1	102 kDa	< 0.00010	KO Synap low, WT Synap high	9	9	12	33	33	34
450	Spectrin alpha chain, non-erythrocytic 1 OS=Mus musculus GN=Sptan1 PE=1 SV=1	Sptan1	285 kDa	0.0059	KO Synap low, WT Synap high	1	28	24	45	42	47
451	Guanine nucleotide-binding protein G(o) subunit alpha OS=Mus musculus GN=Gnao1 PE=1 SV=3	Gnao1	40 kDa	0.0005	KO Synap low, WT Synap high	16	20	20	27	24	23
452	Sodium/potassium-transporting ATPase subunit alpha-2 OS=Mus musculus GN=Atp1a2 PE=1 SV=1	Atp1a2	112 kDa	0.00038	KO Synap low, WT Synap high	19	20	23	34	42	35
453	ATP synthase subunit alpha, mitochondrial OS=Mus musculus GN=Atp5a1 PE=1 SV=1	Atp5a1	60 kDa	< 0.00010	KO Synap low, WT Synap high	1	1	2	28	31	36
454	Synapsin-2 OS=Mus musculus GN=Syn2 PE=1 SV=2	Syn2	63 kDa	< 0.00010	KO Synap low, WT Synap high	11	10	9	20	20	22
455	Neuromodulin OS=Mus musculus GN=Gap43 PE=1 SV=1	Gap43	24 kDa	0.00071	KO Synap low, WT Synap high	17	12	14	21	17	20
456	Calcium/calmodulin-dependent protein kinase type II subunit alpha OS=Mus musculus GN=Camk2a PE=1 SV=2	Camk2a	54 kDa	0.018	KO Synap low, WT Synap high	20	15	13	19	16	17
457	14-3-3 protein zeta/delta OS=Mus musculus GN=Ywhaz PE=1 SV=1	Ywhaz	28 kDa	0.00029	KO Synap low, WT Synap high	14	14	13	16	15	16
458	Malate dehydrogenase, mitochondrial OS=Mus musculus GN=Mdh2 PE=1 SV=3	Mdh2	36 kDa	< 0.00010	KO Synap low, WT Synap high	0	0	0	27	29	31

459	60 kDa heat shock protein, mitochondrial OS=Mus musculus GN=Hspd1 PE=1 SV=1	Hspd1	61 kDa	< 0.00010	KO Synap low, WT Synap high	4	3	3	28	23	32
460	Citrate synthase, mitochondrial OS=Mus musculus GN=Cs PE=1 SV=1	Cs	52 kDa	< 0.00010	KO Synap low, WT Synap high	3	3	3	31	28	30
461	Ras-related protein Rab-3A OS=Mus musculus GN=Rab3a PE=1 SV=1	Rab3a	25 kDa	0.0087	KO Synap low, WT Synap high	14	16	17	15	14	15
462	Syntaxin-1B OS=Mus musculus GN=Stx1b PE=1 SV=1	Stx1b	33 kDa	< 0.00010	KO Synap low, WT Synap high	10	8	11	20	22	21
463	Microtubule-associated protein OS=Mus musculus GN=Mapt PE=1 SV=1	Mapt	76 kDa	0.034	KO Synap low, WT Synap high	20	20	15	19	17	20
464	Synaptosomal-associated protein 25 OS=Mus musculus GN=Snap25 PE=1 SV=1	Snap25	23 kDa	< 0.00010	KO Synap low, WT Synap high	14	14	12	17	17	16
465	Protein kinase C and casein kinase substrate in neurons protein 1 OS=Mus musculus GN=Pacsin1 PE=1 SV=1	Pacsin1	51 kDa	0.023	KO Synap low, WT Synap high	25	25	21	23	18	23
466	Cell cycle exit and neuronal differentiation protein 1 OS=Mus musculus GN=Cend1 PE=1 SV=1	Cend1	15 kDa	0.00022	KO Synap low, WT Synap high	10	8	7	19	20	21
467	V-type proton ATPase catalytic subunit A OS=Mus musculus GN=Atp6v1a PE=1 SV=2	Atp6v1a	68 kDa	0.039	KO Synap low, WT Synap high	13	14	13	15	14	18
468	Creatine kinase U-type, mitochondrial OS=Mus musculus GN=Ckmt1 PE=1 SV=1	Ckmt1	47 kDa	0.0014	KO Synap low, WT Synap high	3	3	3	27	19	23
469	NADH-ubiquinone oxidoreductase 75 kDa subunit, mitochondrial OS=Mus musculus GN=Ndufs1 PE=1 SV=2	Ndufs1	80 kDa	< 0.00010	KO Synap low, WT Synap high	0	0	1	28	28	31
470	Protein bassoon OS=Mus musculus GN=Bsn PE=1 SV=4	Bsn	419 kDa	< 0.00010	KO Synap low, WT Synap high	1	0	1	46	45	48
471	Pyruvate dehydrogenase E1 component subunit beta, mitochondrial OS=Mus musculus GN=Pdhb PE=1 SV=1	Pdhb	39 kDa	< 0.00010	KO Synap low, WT Synap high	1	0	0	17	18	17
472	Endophilin-A1 OS=Mus musculus GN=Sh3gl2 PE=1 SV=1	Sh3gl2	48 kDa	0.007	KO Synap low, WT Synap high	11	16	11	12	12	14
473	Acetyl-CoA acetyltransferase, mitochondrial OS=Mus musculus GN=Acat1 PE=1 SV=1	Acat1	45 kDa	< 0.00010	KO Synap low, WT Synap high	0	0	0	19	19	22
474	Vesicle-associated membrane protein 2 OS=Mus musculus GN=Vamp2 PE=1 SV=1	Vamp2	18 kDa	0.0026	KO Synap low, WT Synap high	11	9	11	10	11	11
475	Calcium-binding mitochondrial carrier protein Aralar1 OS=Mus musculus GN=Slc25a12 PE=1 SV=1	Slc25a12	75 kDa	0.00045	KO Synap low, WT Synap high	0	0	0	23	22	27
476	2-oxoglutarate dehydrogenase, mitochondrial OS=Mus musculus GN=Ogdh PE=1 SV=3	Ogdh	116 kDa	0.00015	KO Synap low, WT Synap high	1	1	1	23	26	30
477	Sodium/potassium-transporting ATPase subunit alpha-1 OS=Mus musculus GN=Atp1a1 PE=1 SV=1	Atp1a1	113 kDa	0.0005	KO Synap low, WT Synap high	12	12	12	14	18	16
478	MICOS complex subunit Mic60 OS=Mus musculus GN=Immt PE=1 SV=1	Immt	84 kDa	< 0.00010	KO Synap low, WT Synap high	0	0	0	28	31	34
479	Septin-7 OS=Mus musculus GN=Sept7 PE=1 SV=2	7-Sep	51 kDa	< 0.00010	KO Synap low, WT Synap high	9	8	7	20	23	18
480	Calcium/calmodulin-dependent protein kinase type II subunit beta OS=Mus musculus GN=Camk2b PE=1 SV=2	Camk2b	60 kDa	0.029	KO Synap low, WT Synap high	13	14	9	16	14	16
481	Stress-70 protein, mitochondrial OS=Mus musculus GN=Hspa9 PE=1 SV=3	Hspa9	73 kDa	< 0.00010	KO Synap low, WT Synap high	0	0	0	24	24	27
482	4-aminobutyrate aminotransferase, mitochondrial OS=Mus musculus GN=Abat PE=1 SV=1	Abat	56 kDa	< 0.00010	KO Synap low, WT Synap high	0	0	0	17	22	21
483	Pyruvate dehydrogenase E1 component subunit alpha, somatic form, mitochondrial OS=Mus musculus GN=Pdha1 PE=1 SV=1	Pdha1	43 kDa	< 0.00010	KO Synap low, WT Synap high	0	0	0	20	15	19
484	Neural cell adhesion molecule 1 OS=Mus musculus GN=Ncam1 PE=1 SV=3	Ncam1	119 kDa	< 0.00010	KO Synap low, WT Synap high	0	0	0	20	22	21
485	ADP/ATP translocase 1 OS=Mus musculus GN=Slc25a4 PE=1 SV=4	Slc25a4	33 kDa	< 0.00010	KO Synap low, WT Synap high	0	0	0	22	20	22
486	Peroxiredoxin-5, mitochondrial OS=Mus musculus GN=Prdx5 PE=1 SV=2	Prdx5	22 kDa	0.00032	KO Synap low, WT Synap high	8	5	5	12	13	15

487	Beta-soluble NSF attachment protein OS=Mus musculus GN=Napb PE=1 SV=2	Napb	34 kDa	0.00049	KO Synap low, WT Synap high	11	7	9	14	15	15
488	Ankyrin-2 OS=Mus musculus GN=Ank2 PE=1 SV=2	Ank2	426 kDa	0.00016	KO Synap low, WT Synap high	6	3	1	31	37	31
489	Isocitrate dehydrogenase [NAD] subunit gamma 1, mitochondrial OS=Mus musculus GN=Idh3g PE=1 SV=1	Idh3g	43 kDa	0.00097	KO Synap low, WT Synap high	0	0	0	10	14	13
490	Isocitrate dehydrogenase [NAD] subunit, mitochondrial OS=Mus musculus GN=Idh3b PE=1 SV=1	Idh3b	42 kDa	0.00012	KO Synap low, WT Synap high	0	0	0	13	17	22
491	Myc box-dependent-interacting protein 1 OS=Mus musculus GN=Bin1 PE=1 SV=1	Bin1	64 kDa	0.0013	KO Synap low, WT Synap high	9	8	5	12	13	15
492	ATP-dependent 6-phosphofructokinase, muscle type OS=Mus musculus GN=Pfkm PE=1 SV=3	Pfkm	85 kDa	0.014	KO Synap low, WT Synap high	9	5	9	15	14	21
493	Glutaminase kidney isoform, mitochondrial OS=Mus musculus GN=Gls PE=1 SV=1	Gls	74 kDa	< 0.00010	KO Synap low, WT Synap high	0	0	0	13	14	17
494	Isocitrate dehydrogenase [NAD] subunit alpha, mitochondrial OS=Mus musculus GN=Idh3a PE=1 SV=1	Idh3a	40 kDa	< 0.00010	KO Synap low, WT Synap high	0	0	0	17	15	20
495	Pyruvate carboxylase OS=Mus musculus GN=Pcx PE=1 SV=1	Pcx	130 kDa	< 0.00010	KO Synap low, WT Synap high	0	0	0	18	19	20
496	Excitatory amino acid transporter 1 OS=Mus musculus GN=Slc1a3 PE=1 SV=2	Slc1a3	60 kDa	0.0019	KO Synap low, WT Synap high	3	0	5	9	9	8
497	Elongation factor Tu, mitochondrial OS=Mus musculus GN=Tufm PE=1 SV=1	Tufm	50 kDa	< 0.00010	KO Synap low, WT Synap high	0	0	0	16	19	19
498	ATPase inhibitor, mitochondrial OS=Mus musculus GN=Atpif1 PE=1 SV=2	Atpif1	12 kDa	0.0019	KO Synap low, WT Synap high	2	2	0	8	10	10
499	Succinate--CoA ligase [ADP-forming] subunit beta, mitochondrial OS=Mus musculus GN=Sucla2 PE=1 SV=2	Sucla2	50 kDa	< 0.00010	KO Synap low, WT Synap high	0	0	0	14	13	14
500	Succinyl-CoA:3-ketoacid coenzyme A transferase 1, mitochondrial OS=Mus musculus GN=Oxct1 PE=1 SV=1	Oxct1	56 kDa	0.00099	KO Synap low, WT Synap high	1	0	0	17	18	18
501	V-type proton ATPase subunit H OS=Mus musculus GN=Atp6v1h PE=1 SV=1	Atp6v1h	54 kDa	0.042	KO Synap low, WT Synap high	10	9	9	9	8	9
502	Glutathione S-transferase Mu 1 OS=Mus musculus GN=Gstm1 PE=1 SV=2	Gstm1	29 kDa	0.03	KO Synap low, WT Synap high	6	5	9	11	8	11
503	Sarcoplasmic/endoplasmic reticulum calcium ATPase 2 OS=Mus musculus GN=Atp2a2 PE=1 SV=2	Atp2a2	115 kDa	0.0016	KO Synap low, WT Synap high	11	11	12	16	16	16
504	NADH dehydrogenase [ubiquinone] flavoprotein 1, mitochondrial OS=Mus musculus GN=Ndufv1 PE=1 SV=1	Ndufv1	50 kDa	< 0.00010	KO Synap low, WT Synap high	0	0	0	12	13	14
505	Alpha-synuclein OS=Mus musculus GN=Snca PE=1 SV=2	Snca	14 kDa	0.0064	KO Synap low, WT Synap high	4	6	9	7	6	5
506	Mitochondrial import receptor subunit TOM70 OS=Mus musculus GN=Tomm70 PE=1 SV=2	Tomm70	68 kDa	< 0.00010	KO Synap low, WT Synap high	0	0	0	15	14	16
507	Protein piccolo OS=Mus musculus GN=Pclo PE=1 SV=4	Pclo	551 kDa	0.0014	KO Synap low, WT Synap high	1	1	0	23	30	22
508	14-3-3 protein eta OS=Mus musculus GN=Ywhah PE=1 SV=2	Ywhah	28 kDa	0.0065	KO Synap low, WT Synap high	9	8	10	9	9	11
509	Rabphilin-3A OS=Mus musculus GN=Rph3a PE=1 SV=2	Rph3a	75 kDa	< 0.00010	KO Synap low, WT Synap high	6	6	6	14	10	12
510	Dynamin-like 120 kDa protein, mitochondrial OS=Mus musculus GN=Opa1 PE=1 SV=1	Opa1	111 kDa	0.00014	KO Synap low, WT Synap high	0	0	0	16	15	19
511	Cytochrome c oxidase subunit 5B, mitochondrial OS=Mus musculus GN=Cox5b PE=1 SV=1	Cox5b	14 kDa	0.00015	KO Synap low, WT Synap high	2	0	2	8	9	9
512	DmX-like protein 2 OS=Mus musculus GN=Dmxl2 PE=1 SV=3	Dmxl2	338 kDa	0.0015	KO Synap low, WT Synap high	8	8	7	15	18	16
513	AP-2 complex subunit mu OS=Mus musculus GN=Ap2m1 PE=1 SV=1	Ap2m1	50 kDa	0.0004	KO Synap low, WT Synap high	5	5	5	8	7	9
514	Amphiphysin OS=Mus musculus GN=Amph PE=1 SV=1	Amph	75 kDa	0.0051	KO Synap low, WT Synap high	7	8	3	12	11	13

515	Guanine nucleotide-binding protein G(I)/G(S)/G(T) subunit beta-1 OS=Mus musculus GN=Gnb1 PE=1 SV=3	Gnb1	37 kDa	< 0.00010	KO Synap low, WT Synap high	8	6	8	11	10	11
516	Succinate dehydrogenase [ubiquinone] flavoprotein subunit, mitochondrial OS=Mus musculus GN=Sdha PE=1 SV=1	Sdha	73 kDa	0.00011	KO Synap low, WT Synap high	0	0	1	17	16	17
517	ProSAAS OS=Mus musculus GN=Pcsk1n PE=1 SV=2	Pcsk1n	27 kDa	0.0057	KO Synap low, WT Synap high	5	5	5	8	6	10
518	Cathepsin D OS=Mus musculus GN=Ctsd PE=1 SV=1	Ctsd	45 kDa	0.0008	KO Synap low, WT Synap high	1	2	2	9	8	7
519	Plasma membrane calcium-transporting ATPase 1 OS=Mus musculus GN=Atp2b1 PE=1 SV=1	Atp2b1	135 kDa	0.00013	KO Synap low, WT Synap high	5	5	6	14	13	10
520	ATP synthase subunit O, mitochondrial OS=Mus musculus GN=Atp5o PE=1 SV=1	Atp5o	23 kDa	0.00013	KO Synap low, WT Synap high	0	0	0	10	11	12
521	Protein Ogdhl OS=Mus musculus GN=Ogdhl PE=1 SV=1	Ogdhl	117 kDa	0.00088	KO Synap low, WT Synap high	0	0	0	11	14	15
522	NADH dehydrogenase [ubiquinone] 1 alpha subcomplex subunit 10, mitochondrial OS=Mus musculus GN=Ndufa10 PE=1 SV=1	Ndufa10	41 kDa	0.00091	KO Synap low, WT Synap high	0	0	0	9	11	11
523	Aldehyde dehydrogenase, mitochondrial OS=Mus musculus GN=Aldh2 PE=1 SV=1	Aldh2	57 kDa	0.00064	KO Synap low, WT Synap high	0	0	0	16	15	12
524	Proline-rich transmembrane protein 2 OS=Mus musculus GN=Prrt2 PE=1 SV=1	Prrt2	36 kDa	0.016	KO Synap low, WT Synap high	2	5	6	7	7	9
525	Trifunctional enzyme subunit alpha, mitochondrial OS=Mus musculus GN=Hadha PE=1 SV=1	Hadha	83 kDa	< 0.00010	KO Synap low, WT Synap high	0	0	0	11	13	9
526	Sodium/potassium-transporting ATPase subunit beta-1 OS=Mus musculus GN=Atp1b1 PE=1 SV=1	Atp1b1	35 kDa	0.009	KO Synap low, WT Synap high	6	7	7	8	6	7
527	Immunoglobulin superfamily member 8 OS=Mus musculus GN=Igsf8 PE=1 SV=1	Igsf8	65 kDa	0.00061	KO Synap low, WT Synap high	5	6	7	9	8	10
528	Septin-11 OS=Mus musculus GN=Sept11 PE=1 SV=4	11-Sep	50 kDa	0.0024	KO Synap low, WT Synap high	4	3	4	11	12	7
529	Tubulin polymerization-promoting protein OS=Mus musculus GN=Tppp PE=1 SV=1	Tppp	24 kDa	0.003	KO Synap low, WT Synap high	5	6	1	12	9	9
530	Sodium- and chloride-dependent GABA transporter 3 OS=Mus musculus GN=Slc6a11 PE=1 SV=2	Slc6a11	70 kDa	0.004	KO Synap low, WT Synap high	5	4	5	8	7	6
531	Cytochrome b-c1 complex subunit 2, mitochondrial OS=Mus musculus GN=Uqcrc2 PE=1 SV=1	Uqcrc2	48 kDa	0.001	KO Synap low, WT Synap high	0	1	0	9	12	11
532	Neurofascin OS=Mus musculus GN=Nfasc PE=1 SV=1	Nfasc	138 kDa	< 0.00010	KO Synap low, WT Synap high	3	2	2	13	14	15
533	SRC kinase-signaling inhibitor 1 OS=Mus musculus GN=Srcin1 PE=1 SV=1	Srcin1	131 kDa	0.00024	KO Synap low, WT Synap high	1	2	0	19	15	18
534	Fumarate hydratase, mitochondrial OS=Mus musculus GN=Fh PE=1 SV=3	Fh	54 kDa	0.0005	KO Synap low, WT Synap high	0	0	1	8	9	8
535	Complexin-2 OS=Mus musculus GN=Cplx2 PE=1 SV=1	Cplx2	15 kDa	0.014	KO Synap low, WT Synap high	6	6	3	4	5	5
536	G protein-regulated inducer of neurite outgrowth 1 OS=Mus musculus GN=Gprin1 PE=1 SV=2	Gprin1	95 kDa	0.0011	KO Synap low, WT Synap high	7	7	10	12	14	14
537	Protein Rab1a OS=Mus musculus GN=Rab1a PE=1 SV=1	Rab1a	22 kDa	0.045	KO Synap low, WT Synap high	7	5	5	5	5	4
538	10 kDa heat shock protein, mitochondrial OS=Mus musculus GN=Hspe1 PE=1 SV=2	Hspe1	11 kDa	0.00024	KO Synap low, WT Synap high	1	0	0	8	7	9
539	NADH dehydrogenase [ubiquinone] 1 alpha subcomplex subunit 9, mitochondrial OS=Mus musculus GN=Ndufa9 PE=1 SV=1	Ndufa9	42 kDa	0.00012	KO Synap low, WT Synap high	0	0	0	11	11	11
540	Cytochrome c, somatic OS=Mus musculus GN=Cycs PE=1 SV=2	Cycs	12 kDa	< 0.00010	KO Synap low, WT Synap high	0	0	0	7	7	6
541	NADH dehydrogenase [ubiquinone] iron-sulfur protein 2, mitochondrial OS=Mus musculus GN=Ndufs2 PE=1 SV=1	Ndufs2	53 kDa	0.0051	KO Synap low, WT Synap high	0	0	0	4	6	9
542	Putative adenosylhomocysteinase 2 OS=Mus musculus GN=Ahcyl1 PE=1 SV=1	Ahcyl1	59 kDa	0.0061	KO Synap low, WT Synap high	8	2	8	9	11	10

543	Ras/Rap GTPase-activating protein SynGAP (Fragment) OS=Mus musculus GN=Syngap1 PE=1 SV=2	Syngap1	142 kDa	0.00035	KO Synap low, WT Synap high	3	4	0	9	12	10
544	Septin-5 OS=Mus musculus GN=Sept5 PE=1 SV=2	5-Sep	43 kDa	0.0022	KO Synap low, WT Synap high	2	1	2	8	7	7
545	Guanine nucleotide-binding protein G(I)/G(S)/G(T) subunit beta-2 OS=Mus musculus GN=Gnb2 PE=1 SV=1	Gnb2	41 kDa	0.00033	KO Synap low, WT Synap high	5	5	5	7	7	7
546	Cytochrome b-c1 complex subunit 1, mitochondrial OS=Mus musculus GN=Uqcrc1 PE=1 SV=2	Uqcrc1	53 kDa	0.00038	KO Synap low, WT Synap high	0	2	2	5	8	9
547	Septin-6 OS=Mus musculus GN=Sept6 PE=1 SV=4	6-Sep	50 kDa	0.00021	KO Synap low, WT Synap high	3	3	3	5	7	6
548	NADH dehydrogenase [ubiquinone] 1 alpha subcomplex subunit 12 OS=Mus musculus GN=Ndufa12 PE=1 SV=1	Ndufa12	18 kDa	< 0.00010	KO Synap low, WT Synap high	0	0	0	8	8	10
549	Actin-related protein 2 OS=Mus musculus GN=Actr2 PE=1 SV=1	Actr2	45 kDa	0.048	KO Synap low, WT Synap high	6	7	6	7	5	8
550	Clathrin coat assembly protein AP180 OS=Mus musculus GN=Snap91 PE=1 SV=1	Snap91	92 kDa	0.03	KO Synap low, WT Synap high	5	3	5	6	6	9
551	NADH dehydrogenase [ubiquinone] flavoprotein 2, mitochondrial OS=Mus musculus GN=Ndufv2 PE=1 SV=2	Ndufv2	27 kDa	< 0.00010	KO Synap low, WT Synap high	0	0	0	8	7	5
552	NADH dehydrogenase [ubiquinone] iron-sulfur protein 3, mitochondrial OS=Mus musculus GN=Ndufs3 PE=1 SV=2	Ndufs3	30 kDa	< 0.00010	KO Synap low, WT Synap high	0	0	0	9	11	11
553	Dihydrolipoyllysine-residue acetyltransferase component of pyruvate dehydrogenase complex, mitochondrial OS=Mus musculus GN=Dlat PE=1 SV=2	Dlat	68 kDa	0.00045	KO Synap low, WT Synap high	0	0	0	8	8	12
554	Dihydrolipoyl dehydrogenase, mitochondrial OS=Mus musculus GN=Dld PE=1 SV=2	Dld	54 kDa	< 0.00010	KO Synap low, WT Synap high	0	0	0	10	12	11
555	Cathepsin B OS=Mus musculus GN=Ctsb PE=1 SV=2	Ctsb	37 kDa	0.014	KO Synap low, WT Synap high	2	3	3	9	6	9
556	Succinate--CoA ligase [ADP/GDP-forming] subunit alpha, mitochondrial OS=Mus musculus GN=Suclg1 PE=1 SV=4	Suclg1	36 kDa	0.00028	KO Synap low, WT Synap high	2	2	0	6	5	6
557	Methylmalonate-semialdehyde dehydrogenase [acylating], mitochondrial OS=Mus musculus GN=Aldh6a1 PE=1 SV=1	Aldh6a1	58 kDa	< 0.00010	KO Synap low, WT Synap high	0	0	0	8	7	9
558	Calcium-transporting ATPase OS=Mus musculus GN=Atp2b3 PE=1 SV=1	Atp2b3	129 kDa	< 0.00010	KO Synap low, WT Synap high	3	5	6	12	12	14
559	Isocitrate dehydrogenase [NADP], mitochondrial OS=Mus musculus GN=Idh2 PE=1 SV=3	Idh2	51 kDa	< 0.00010	KO Synap low, WT Synap high	0	0	0	8	7	9
560	Capping protein (Actin filament) muscle Z-line, beta, isoform CRA_a OS=Mus musculus GN=Capzb PE=1 SV=1	Capzb	29 kDa	0.014	KO Synap low, WT Synap high	6	5	6	4	6	5
561	MICOS complex subunit Mic19 OS=Mus musculus GN=Chchd3 PE=1 SV=1	Chchd3	26 kDa	0.00085	KO Synap low, WT Synap high	0	0	0	13	10	12
562	Electron transfer flavoprotein subunit alpha, mitochondrial OS=Mus musculus GN=Etfa PE=1 SV=2	Etfa	35 kDa	0.00047	KO Synap low, WT Synap high	0	0	0	7	6	11
563	Transforming protein RhoA OS=Mus musculus GN=Rhoa PE=1 SV=1	Rhoa	22 kDa	0.00084	KO Synap low, WT Synap high	4	3	3	6	6	9
564	Solute carrier family 12 member 5 OS=Mus musculus GN=Slc12a5 PE=1 SV=2	Slc12a5	126 kDa	< 0.00010	KO Synap low, WT Synap high	2	3	3	7	9	9
565	Inositol 1,4,5-trisphosphate receptor type 1 OS=Mus musculus GN=Itpr1 PE=1 SV=2	Itpr1	313 kDa	0.00031	KO Synap low, WT Synap high	6	3	4	11	13	12
566	AP-2 complex subunit alpha-1 OS=Mus musculus GN=Ap2a1 PE=1 SV=1	Ap2a1	108 kDa	0.031	KO Synap low, WT Synap high	4	7	3	6	11	9
567	Synaptic vesicle glycoprotein 2A OS=Mus musculus GN=Sv2a PE=1 SV=1	Sv2a	83 kDa	0.0065	KO Synap low, WT Synap high	7	5	6	9	9	9
568	LETM1 and EF-hand domain-containing protein 1, mitochondrial OS=Mus musculus GN=Letm1 PE=1 SV=1	Letm1	83 kDa	0.0002	KO Synap low, WT Synap high	0	0	0	9	11	9
569	Sodium- and chloride-dependent GABA transporter 1 OS=Mus musculus GN=Slc6a1 PE=1 SV=2	Slc6a1	67 kDa	0.00017	KO Synap low, WT Synap high	3	2	1	3	3	3
570	Electron transfer flavoprotein subunit beta OS=Mus musculus GN=Etfb PE=1 SV=3	Etfb	28 kDa	0.00047	KO Synap low, WT Synap high	0	0	0	7	8	8

571	NADH dehydrogenase [ubiquinone] 1 alpha subcomplex subunit 8 OS=Mus musculus GN=Ndufa8 PE=1 SV=3	Ndufa8	20 kDa	0.00041	KO Synap low, WT Synap high	0	0	0	5	5	5
572	Phosphate carrier protein, mitochondrial OS=Mus musculus GN=Slc25a3 PE=1 SV=1	Slc25a3	40 kDa	< 0.00010	KO Synap low, WT Synap high	0	0	0	7	6	7
573	Limbic system-associated membrane protein OS=Mus musculus GN=Lsamp PE=1 SV=1	Lsamp	39 kDa	0.0006	KO Synap low, WT Synap high	0	2	2	5	5	8
574	ATP synthase subunit d, mitochondrial OS=Mus musculus GN=Atp5h PE=1 SV=3	Atp5h	19 kDa	0.002	KO Synap low, WT Synap high	0	0	0	6	10	10
575	Transgelin-3 OS=Mus musculus GN=Tagln3 PE=1 SV=1	Tagln3	22 kDa	0.0006	KO Synap low, WT Synap high	4	5	4	6	8	7
576	Cytochrome c oxidase subunit NDUFA4 OS=Mus musculus GN=Ndufa4 PE=1 SV=2	Ndufa4	9 kDa	0.0026	KO Synap low, WT Synap high	0	0	0	10	9	9
577	AP-2 complex subunit alpha-2 OS=Mus musculus GN=Ap2a2 PE=1 SV=2	Ap2a2	104 kDa	0.01	KO Synap low, WT Synap high	5	4	4	6	7	9
578	Complement component 1 Q subcomponent-binding protein, mitochondrial OS=Mus musculus GN=C1qbp PE=1 SV=1	C1qbp	31 kDa	0.0007	KO Synap low, WT Synap high	0	0	0	5	4	5
579	NADH dehydrogenase [ubiquinone] iron-sulfur protein 6, mitochondrial OS=Mus musculus GN=Ndufs6 PE=1 SV=2	Ndufs6	13 kDa	< 0.00010	KO Synap low, WT Synap high	0	0	0	8	8	9
580	RAS-related C3 botulinum substrate 1, isoform CRA_a OS=Mus musculus GN=Rac1 PE=1 SV=1	Rac1	23 kDa	< 0.00010	KO Synap low, WT Synap high	3	3	3	5	6	6
581	Cytochrome b-c1 complex subunit 6, mitochondrial OS=Mus musculus GN=Uqcrh PE=1 SV=2	Uqcrh	10 kDa	0.0001	KO Synap low, WT Synap high	0	0	0	4	3	3
582	Myelin proteolipid protein OS=Mus musculus GN=Plp1 PE=1 SV=2	Plp1	30 kDa	0.00052	KO Synap low, WT Synap high	2	3	2	2	3	3
583	Contactin-1 OS=Mus musculus GN=Cntn1 PE=1 SV=1	Cntn1	113 kDa	0.00016	KO Synap low, WT Synap high	2	1	1	7	8	11
584	Neuronal-specific septin-3 OS=Mus musculus GN=Sept3 PE=1 SV=2	3-Sep	40 kDa	0.00096	KO Synap low, WT Synap high	2	3	2	6	9	8
585	ATP synthase F(0) complex subunit B1, mitochondrial OS=Mus musculus GN=Atp5f1 PE=1 SV=1	Atp5f1	29 kDa	0.00022	KO Synap low, WT Synap high	0	1	1	7	7	6
586	Caskin-1 OS=Mus musculus GN=Caskin1 PE=1 SV=2	Caskin1	150 kDa	0.0025	KO Synap low, WT Synap high	2	3	2	12	11	10
587	3-hydroxyacyl-CoA dehydrogenase type-2 OS=Mus musculus GN=Hsd17b10 PE=1 SV=1	Hsd17b10	28 kDa	0.00025	KO Synap low, WT Synap high	0	0	0	5	7	6
588	Mitochondrial glutamate carrier 1 OS=Mus musculus GN=Slc25a22 PE=1 SV=1	Slc25a22	35 kDa	< 0.00010	KO Synap low, WT Synap high	0	0	0	7	7	6
589	Syntaxin-1A OS=Mus musculus GN=Stx1a PE=1 SV=1	Stx1a	29 kDa	< 0.00010	KO Synap low, WT Synap high	2	2	3	6	7	7
590	Septin-8 OS=Mus musculus GN=Sept8 PE=1 SV=1	8-Sep	56 kDa	0.0014	KO Synap low, WT Synap high	0	0	0	7	9	8
591	14-3-3 protein gamma OS=Mus musculus GN=Ywhag PE=1 SV=2	Ywhag	28 kDa	0.037	KO Synap low, WT Synap high	3	3	4	4	7	6
592	NADH dehydrogenase [ubiquinone] 1 alpha subcomplex subunit 7 OS=Mus musculus GN=Ndufa7 PE=1 SV=3	Ndufa7	13 kDa	0.0043	KO Synap low, WT Synap high	0	0	0	9	9	10
593	ATP synthase subunit gamma OS=Mus musculus GN=Atp5c1 PE=1 SV=1	Atp5c1	33 kDa	0.0031	KO Synap low, WT Synap high	0	0	0	7	6	8
594	D-beta-hydroxybutyrate dehydrogenase, mitochondrial OS=Mus musculus GN=Bdh1 PE=1 SV=2	Bdh1	38 kDa	0.00051	KO Synap low, WT Synap high	0	0	0	7	7	6
595	Plectin OS=Mus musculus GN=Plec PE=1 SV=3	Plec	534 kDa	0.012	KO Synap low, WT Synap high	6	1	1	12	9	10
596	Disks large homolog 4 OS=Mus musculus GN=Dlg4 PE=1 SV=1	Dlg4	80 kDa	< 0.00010	KO Synap low, WT Synap high	0	1	0	8	8	9
597	Sideroflexin-3 OS=Mus musculus GN=Sfxn3 PE=1 SV=1	Sfxn3	35 kDa	< 0.00010	KO Synap low, WT Synap high	0	1	0	5	7	5
598	NADH dehydrogenase [ubiquinone] 1 beta subcomplex subunit 4 OS=Mus musculus GN=Ndufb4 PE=1 SV=3	Ndufb4	15 kDa	0.00049	KO Synap low, WT Synap high	0	0	0	7	6	5

599	Protein NipSnap homolog 2 OS=Mus musculus GN=Gbas PE=1 SV=1	Gbas	33 kDa	< 0.00010	KO Synap low, WT Synap high	0	0	0	5	5	6
600	Succinate-semialdehyde dehydrogenase, mitochondrial OS=Mus musculus GN=Aldh5a1 PE=1 SV=1	Aldh5a1	56 kDa	< 0.00010	KO Synap low, WT Synap high	0	0	0	5	6	6
601	Nucleoside diphosphate kinase A OS=Mus musculus GN=Nme1 PE=1 SV=1	Nme1	17 kDa	0.011	KO Synap low, WT Synap high	0	2	3	3	5	3
602	NADH dehydrogenase [ubiquinone] 1 alpha subcomplex subunit 5 OS=Mus musculus GN=Ndufa5 PE=1 SV=3	Ndufa5	13 kDa	0.00046	KO Synap low, WT Synap high	0	0	0	4	4	6
603	Cytochrome b-c1 complex subunit Rieske, mitochondrial OS=Mus musculus GN=Uqcrfs1 PE=1 SV=1	Uqcrfs1	29 kDa	< 0.00010	KO Synap low, WT Synap high	0	0	0	4	4	4
604	NADH dehydrogenase [ubiquinone] iron-sulfur protein 8, mitochondrial OS=Mus musculus GN=Ndufs8 PE=1 SV=1	Ndufs8	24 kDa	0.0033	KO Synap low, WT Synap high	0	0	0	9	7	9
605	NADH dehydrogenase [ubiquinone] 1 alpha subcomplex subunit 13 OS=Mus musculus GN=Ndufa13 PE=1 SV=3	Ndufa13	17 kDa	0.0032	KO Synap low, WT Synap high	0	0	0	5	4	3
606	Thiosulfate sulfurtransferase OS=Mus musculus GN=Tst PE=1 SV=3	Tst	33 kDa	< 0.00010	KO Synap low, WT Synap high	0	0	0	7	5	7
607	Vesicle-associated membrane protein-associated protein A OS=Mus musculus GN=Vapa PE=1 SV=2	Vapa	28 kDa	0.00091	KO Synap low, WT Synap high	2	2	2	4	3	4
608	3-ketoacyl-CoA thiolase, mitochondrial OS=Mus musculus GN=Acaa2 PE=1 SV=3	Acaa2	42 kDa	< 0.00010	KO Synap low, WT Synap high	0	0	0	5	9	9
609	ADP/ATP translocase 2 OS=Mus musculus GN=Slc25a5 PE=1 SV=3	Slc25a5	33 kDa	< 0.00010	KO Synap low, WT Synap high	0	0	0	5	7	8
610	Neurofilament light polypeptide OS=Mus musculus GN=Nefl PE=1 SV=5	Nefl	62 kDa	0.046	KO Synap low, WT Synap high	1	2	1	6	3	4
611	NADH dehydrogenase [ubiquinone] 1 alpha subcomplex subunit 6 OS=Mus musculus GN=Ndufa6 PE=1 SV=1	Ndufa6	15 kDa	0.0014	KO Synap low, WT Synap high	1	0	0	3	3	4
612	Cytochrome c oxidase subunit 6B1 OS=Mus musculus GN=Cox6b1 PE=1 SV=2	Cox6b1	10 kDa	0.0018	KO Synap low, WT Synap high	0	0	0	3	5	5
613	NADH dehydrogenase [ubiquinone] iron-sulfur protein 7, mitochondrial OS=Mus musculus GN=Ndufs7 PE=1 SV=1	Ndufs7	25 kDa	< 0.00010	KO Synap low, WT Synap high	0	0	0	6	5	5
614	Trifunctional enzyme subunit beta, mitochondrial OS=Mus musculus GN=Hadhb PE=1 SV=1	Hadhb	51 kDa	< 0.00010	KO Synap low, WT Synap high	0	0	0	4	4	5
615	Serine/threonine-protein phosphatase OS=Mus musculus GN=Ppp3cb PE=1 SV=1	Ppp3cb	59 kDa	0.036	KO Synap low, WT Synap high	5	4	3	3	5	4
616	Thioredoxin-dependent peroxide reductase, mitochondrial OS=Mus musculus GN=Prdx3 PE=1 SV=1	Prdx3	28 kDa	< 0.00010	KO Synap low, WT Synap high	0	0	0	6	6	6
617	Ornithine aminotransferase, mitochondrial OS=Mus musculus GN=Oat PE=1 SV=1	Oat	48 kDa	0.0087	KO Synap low, WT Synap high	0	0	0	8	3	5
618	Lon protease homolog, mitochondrial OS=Mus musculus GN=Lonp1 PE=1 SV=2	Lonp1	106 kDa	0.0007	KO Synap low, WT Synap high	0	0	0	5	7	5
619	LanC-like protein 2 (Fragment) OS=Mus musculus GN=Lancl2 PE=1 SV=1	Lancl2	50 kDa	0.034	KO Synap low, WT Synap high	4	2	2	5	6	5
620	Alpha-internexin OS=Mus musculus GN=Ina PE=1 SV=3	Ina	55 kDa	0.0013	KO Synap low, WT Synap high	1	0	1	6	8	6
621	Guanine nucleotide-binding protein G(i) subunit alpha-2 OS=Mus musculus GN=Gnai2 PE=1 SV=5	Gnai2	40 kDa	0.0012	KO Synap low, WT Synap high	2	0	2	5	3	6
622	ATP synthase subunit e, mitochondrial OS=Mus musculus GN=Atp5i PE=1 SV=2	Atp5i	8 kDa	0.00047	KO Synap low, WT Synap high	0	0	0	4	3	3
623	GTPase HRas OS=Mus musculus GN=Hras PE=1 SV=2	Hras	21 kDa	0.0019	KO Synap low, WT Synap high	0	2	2	5	5	5
624	Neurotrimin OS=Mus musculus GN=Ntm PE=1 SV=1	Ntm	35 kDa	0.0076	KO Synap low, WT Synap high	3	2	1	7	4	3
625	Leucine-rich PPR motif-containing protein, mitochondrial OS=Mus musculus GN=Lrpprc PE=1 SV=2	Lrpprc	157 kDa	0.0017	KO Synap low, WT Synap high	0	0	0	8	6	11
626	Basigin (Fragment) OS=Mus musculus GN=Bsg PE=1 SV=1	Bsg	22 kDa	0.014	KO Synap low, WT Synap high	2	3	3	4	3	4

627	Cytoplasmic FMR1-interacting protein 2 OS=Mus musculus GN=Cyfip2 PE=1 SV=2	Cyfip2	146 kDa	0.0076	KO Synap low, WT Synap high	3	3	3	5	3	5
628	Brain-specific angiogenesis inhibitor 1-associated protein 2 OS=Mus musculus GN=Baiap2 PE=1 SV=1	Baiap2	58 kDa	< 0.00010	KO Synap low, WT Synap high	1	0	1	8	8	7
629	Propionyl-CoA carboxylase beta chain, mitochondrial OS=Mus musculus GN=Pccb PE=1 SV=2	Pccb	58 kDa	0.002	KO Synap low, WT Synap high	0	0	0	6	6	5
630	Oligodendrocyte myelin glycoprotein OS=Mus musculus GN=Omg PE=1 SV=1	Omg	50 kDa	0.0044	KO Synap low, WT Synap high	2	2	1	3	4	3
631	Long chain acyl-CoA synthetase 6 isoform 3 OS=Mus musculus GN=Acsl6 PE=1 SV=1	Acsl6	78 kDa	0.0017	KO Synap low, WT Synap high	2	1	2	5	4	6
632	Visinin-like protein 1 OS=Mus musculus GN=Vsnl1 PE=1 SV=2	Vsnl1	22 kDa	0.0015	KO Synap low, WT Synap high	0	1	0	4	6	5
633	AFG3-like protein 2 OS=Mus musculus GN=Afg3l2 PE=1 SV=1	Afg3l2	90 kDa	0.00019	KO Synap low, WT Synap high	0	0	0	6	7	5
634	Neuronal membrane glycoprotein M6-a OS=Mus musculus GN=Gpm6a PE=1 SV=1	Gpm6a	31 kDa	0.0021	KO Synap low, WT Synap high	2	2	2	4	5	3
635	Actin-related protein 2/3 complex subunit 2 OS=Mus musculus GN=Arpc2 PE=1 SV=3	Arpc2	34 kDa	0.039	KO Synap low, WT Synap high	2	0	3	2	3	3
636	ATP synthase-coupling factor 6, mitochondrial OS=Mus musculus GN=Atp5j PE=1 SV=1	Atp5j	12 kDa	0.0048	KO Synap low, WT Synap high	0	0	0	4	3	5
637	Enoyl-CoA hydratase, mitochondrial OS=Mus musculus GN=Echs1 PE=1 SV=1	Echs1	31 kDa	0.0027	KO Synap low, WT Synap high	0	0	0	4	5	7
638	Cytochrome c1, heme protein, mitochondrial OS=Mus musculus GN=Cyc1 PE=1 SV=1	Cyc1	35 kDa	0.016	KO Synap low, WT Synap high	0	0	0	2	4	3
639	Guanine nucleotide-binding protein G(I)/G(S)/G(O) subunit gamma-3 OS=Mus musculus GN=Gng3 PE=1 SV=1	Gng3	8 kDa	0.021	KO Synap low, WT Synap high	1	1	1	2	2	2
640	Pyruvate dehydrogenase protein X component, mitochondrial OS=Mus musculus GN=Pdhx PE=1 SV=1	Pdhx	54 kDa	< 0.00010	KO Synap low, WT Synap high	0	0	0	4	5	5
641	ES1 protein homolog, mitochondrial OS=Mus musculus GN=D10Jhu81e PE=1 SV=1	D10Jhu81e	28 kDa	0.0011	KO Synap low, WT Synap high	0	0	0	4	5	4
642	Sideroflexin-1 OS=Mus musculus GN=Sfxn1 PE=1 SV=3	Sfxn1	36 kDa	0.0016	KO Synap low, WT Synap high	0	0	0	5	6	6
643	Mitochondrial 2-oxoglutarate/malate carrier protein OS=Mus musculus GN=Slc25a11 PE=1 SV=3	Slc25a11	34 kDa	0.0044	KO Synap low, WT Synap high	0	0	0	4	5	4
644	Fumarylacetoacetate hydrolase domain-containing protein 2A OS=Mus musculus GN=Fahd2a PE=1 SV=1	Fahd2a	35 kDa	< 0.00010	KO Synap low, WT Synap high	0	0	0	3	5	2
645	Ganglioside-induced differentiation-associated protein 1-like 1 OS=Mus musculus GN=Gdap1l1 PE=1 SV=1	Gdap1l1	42 kDa	0.043	KO Synap low, WT Synap high	0	1	1	2	3	4
646	Methylglutaconyl-CoA hydratase, mitochondrial OS=Mus musculus GN=Auh PE=1 SV=1	Auh	33 kDa	< 0.00010	KO Synap low, WT Synap high	0	0	0	5	7	6
647	ATP synthase subunit delta, mitochondrial OS=Mus musculus GN=Atp5d PE=1 SV=1	Atp5d	18 kDa	0.0076	KO Synap low, WT Synap high	0	0	0	3	2	3
648	Secretogranin-2 OS=Mus musculus GN=Scg2 PE=1 SV=1	Scg2	71 kDa	0.0035	KO Synap low, WT Synap high	3	2	1	6	6	5
649	WD repeat-containing protein 7 OS=Mus musculus GN=Wdr7 PE=1 SV=3	Wdr7	163 kDa	0.00044	KO Synap low, WT Synap high	0	0	0	5	8	3
650	Very long-chain specific acyl-CoA dehydrogenase, mitochondrial OS=Mus musculus GN=Acadvl PE=1 SV=3	Acadvl	71 kDa	0.0013	KO Synap low, WT Synap high	0	0	0	6	6	8
651	Propionyl-CoA carboxylase alpha chain, mitochondrial OS=Mus musculus GN=Pcca PE=1 SV=2	Pcca	80 kDa	< 0.00010	KO Synap low, WT Synap high	0	0	0	4	4	4
652	Cytochrome c oxidase subunit 7A2, mitochondrial OS=Mus musculus GN=Cox7a2 PE=1 SV=2	Cox7a2	9 kDa	0.00046	KO Synap low, WT Synap high	0	0	0	3	3	3
653	NADH dehydrogenase [ubiquinone] 1 subunit C2 OS=Mus musculus GN=Ndufc2 PE=1 SV=1	Ndufc2	14 kDa	0.0044	KO Synap low, WT Synap high	0	0	0	4	4	2
654	Calcium-transporting ATPase OS=Mus musculus GN=Atp2b4 PE=1 SV=1	Atp2b4	129 kDa	0.00036	KO Synap low, WT Synap high	2	2	2	4	4	5

655	CDGSH iron-sulfur domain-containing protein 1 OS=Mus musculus GN=Cisd1 PE=1 SV=1	Cisd1	12 kDa	0.0028	KO Synap low, WT Synap high	0	1	1	3	3	3
656	Phospholipase D3 OS=Mus musculus GN=Pld3 PE=1 SV=1	Pld3	54 kDa	0.00014	KO Synap low, WT Synap high	0	0	0	3	2	3
657	Leucine-rich glioma-inactivated protein 1 OS=Mus musculus GN=Lgi1 PE=1 SV=1	Lgi1	64 kDa	0.0031	KO Synap low, WT Synap high	0	0	0	6	6	4
658	Paralemmin-1 OS=Mus musculus GN=Palm PE=1 SV=1	Palm	42 kDa	0.024	KO Synap low, WT Synap high	1	2	1	4	3	2
659	Catenin alpha-2 OS=Mus musculus GN=Ctnna2 PE=1 SV=3	Ctnna2	105 kDa	0.019	KO Synap low, WT Synap high	1	1	0	2	3	5
660	Glycerol-3-phosphate dehydrogenase, mitochondrial OS=Mus musculus GN=Gpd2 PE=1 SV=2	Gpd2	81 kDa	0.00049	KO Synap low, WT Synap high	0	0	0	4	4	3
661	Cysteine and glycine-rich protein 1 OS=Mus musculus GN=Csrp1 PE=1 SV=3	Csrp1	21 kDa	0.023	KO Synap low, WT Synap high	0	0	2	4	3	3
662	NADH dehydrogenase [ubiquinone] 1 alpha subcomplex subunit 2 OS=Mus musculus GN=Ndufa2 PE=1 SV=3	Ndufa2	11 kDa	0.0092	KO Synap low, WT Synap high	1	1	1	3	2	3
663	Disintegrin and metalloproteinase domain-containing protein 22 OS=Mus musculus GN=Adam22 PE=1 SV=2	Adam22	100 kDa	< 0.00010	KO Synap low, WT Synap high	0	0	0	5	6	4
664	Uncharacterized protein KIAA0513 OS=Mus musculus GN=Kiaa0513 PE=1 SV=1	Kiaa0513	46 kDa	0.0044	KO Synap low, WT Synap high	0	0	2	7	4	5
665	ATP synthase subunit g, mitochondrial OS=Mus musculus GN=Atp5l PE=1 SV=1	Atp5l	11 kDa	0.0027	KO Synap low, WT Synap high	0	0	0	2	2	2
666	Protein NipSnap homolog 1 OS=Mus musculus GN=Nipsnap1 PE=1 SV=1	Nipsnap1	33 kDa	0.0058	KO Synap low, WT Synap high	0	0	0	4	2	4
667	Catenin delta-2 OS=Mus musculus GN=Ctnnd2 PE=1 SV=1	Ctnnd2	135 kDa	0.0013	KO Synap low, WT Synap high	1	1	1	5	4	6
668	Hepatocyte cell adhesion molecule OS=Mus musculus GN=Hepacam PE=1 SV=2	Hepacam	46 kDa	0.043	KO Synap low, WT Synap high	2	0	2	2	2	2
669	Neural cell adhesion molecule L1 OS=Mus musculus GN=L1cam PE=1 SV=1	L1cam	140 kDa	0.0013	KO Synap low, WT Synap high	0	0	0	3	4	4
670	Mitochondrial import receptor subunit TOM22 homolog OS=Mus musculus GN=Tomm22 PE=1 SV=3	Tomm22	16 kDa	0.00044	KO Synap low, WT Synap high	0	0	0	3	3	4
671	Metaxin-2 OS=Mus musculus GN=Mtx2 PE=1 SV=1	Mtx2	30 kDa	0.0032	KO Synap low, WT Synap high	0	0	0	2	4	3
672	Ankyrin-2 (Fragment) OS=Mus musculus GN=Ank2 PE=1 SV=2	Ank2	131 kDa	0.0034	KO Synap low, WT Synap high	1	0	0	3	3	2
673	Homer protein homolog 1 OS=Mus musculus GN=Homer1 PE=1 SV=2	Homer1	41 kDa	0.007	KO Synap low, WT Synap high	1	0	0	3	6	3
674	Syntaxin-12 OS=Mus musculus GN=Stx12 PE=1 SV=1	Stx12	31 kDa	< 0.00010	KO Synap low, WT Synap high	0	0	0	3	4	4
675	Mitochondrial import inner membrane translocase subunit Tim9 OS=Mus musculus GN=Timm9 PE=1 SV=1	Timm9	10 kDa	0.0086	KO Synap low, WT Synap high	0	0	0	4	3	4
676	EH domain-containing protein 3 OS=Mus musculus GN=Ehd3 PE=1 SV=2	Ehd3	61 kDa	0.031	KO Synap low, WT Synap high	2	1	0	3	5	2
677	Rho-related GTP-binding protein RhoB OS=Mus musculus GN=Rhob PE=1 SV=1	Rhob	22 kDa	0.017	KO Synap low, WT Synap high	1	1	1	4	3	4
678	Alpha-actinin-1 OS=Mus musculus GN=Actn1 PE=1 SV=1	Actn1	103 kDa	0.013	KO Synap low, WT Synap high	1	1	0	3	3	3
679	Voltage-dependent anion-selective channel protein 1 OS=Mus musculus GN=Vdac1 PE=1 SV=3	Vdac1	32 kDa	0.0029	KO Synap low, WT Synap high	0	1	0	2	3	3
680	Succinate dehydrogenase [ubiquinone] iron-sulfur subunit, mitochondrial OS=Mus musculus GN=Sdhb PE=1 SV=1	Sdhb	32 kDa	0.0028	KO Synap low, WT Synap high	0	0	0	3	6	4
681	Talin-2 OS=Mus musculus GN=Tln2 PE=1 SV=1	Tln2	272 kDa	0.036	KO Synap low, WT Synap high	3	1	1	3	5	4
682	Breast carcinoma-amplified sequence 1 homolog OS=Mus musculus GN=Bcas1 PE=1 SV=3	Bcas1	67 kDa	0.0039	KO Synap low, WT Synap high	1	1	0	5	3	4

683	MICOS complex subunit Mic25 OS=Mus musculus GN=Chchd6 PE=1 SV=2	Chchd6	30 kDa	0.011	KO Synap low, WT Synap high	0	0	0	8	6	3
684	C2 domain-containing protein 2-like OS=Mus musculus GN=C2cd2l PE=1 SV=3	C2cd2l	76 kDa	0.0047	KO Synap low, WT Synap high	0	2	0	3	5	4
685	Mitochondrial amidoxime reducing component 2 OS=Mus musculus GN=Marc2 PE=1 SV=1	2-Mar	38 kDa	0.0024	KO Synap low, WT Synap high	0	0	0	4	5	4
686	NADH dehydrogenase [ubiquinone] flavoprotein 3, mitochondrial OS=Mus musculus GN=Ndufv3 PE=1 SV=1	Ndufv3	12 kDa	0.00028	KO Synap low, WT Synap high	0	0	0	1	1	1
687	Kinesin-like protein OS=Mus musculus GN=Kif2a PE=1 SV=1	Kif2a	84 kDa	0.0052	KO Synap low, WT Synap high	2	0	0	3	4	4
688	Acyl-CoA dehydrogenase family member 9, mitochondrial OS=Mus musculus GN=Acad9 PE=1 SV=2	Acad9	69 kDa	0.005	KO Synap low, WT Synap high	0	0	0	5	6	3
689	Synaptic vesicle glycoprotein 2B OS=Mus musculus GN=Sv2b PE=1 SV=1	Sv2b	77 kDa	0.031	KO Synap low, WT Synap high	0	0	0	2	3	7
690	Inorganic pyrophosphatase 2, mitochondrial OS=Mus musculus GN=Ppa2 PE=1 SV=1	Ppa2	38 kDa	0.0026	KO Synap low, WT Synap high	0	0	1	3	3	4
691	Prohibitin-2 OS=Mus musculus GN=Phb2 PE=1 SV=1	Phb2	33 kDa	0.0013	KO Synap low, WT Synap high	0	0	0	3	5	5
692	Protein Pcdh1 OS=Mus musculus GN=Pcdh1 PE=1 SV=1	Pcdh1	112 kDa	0.0059	KO Synap low, WT Synap high	0	0	0	5	4	3
693	Succinate--CoA ligase [GDP-forming] subunit beta, mitochondrial OS=Mus musculus GN=Suclg2 PE=1 SV=3	Suclg2	47 kDa	0.0059	KO Synap low, WT Synap high	0	0	0	5	3	3
694	Disintegrin and metalloproteinase domain-containing protein 23 OS=Mus musculus GN=Adam23 PE=1 SV=1	Adam23	92 kDa	0.00051	KO Synap low, WT Synap high	0	0	0	4	4	4
695	Vesicular inhibitory amino acid transporter OS=Mus musculus GN=Slc32a1 PE=1 SV=3	Slc32a1	57 kDa	0.015	KO Synap low, WT Synap high	0	0	0	3	2	2
696	Neurofilament heavy polypeptide OS=Mus musculus GN=Nefh PE=1 SV=3	Nefh	117 kDa	0.0059	KO Synap low, WT Synap high	1	0	0	4	3	2
697	Ganglioside-induced differentiation-associated protein 1 OS=Mus musculus GN=Gdap1 PE=1 SV=1	Gdap1	41 kDa	0.00044	KO Synap low, WT Synap high	0	0	0	4	6	6
698	Mitochondrial glutamate carrier 2 OS=Mus musculus GN=Slc25a18 PE=1 SV=4	Slc25a18	34 kDa	0.00085	KO Synap low, WT Synap high	0	0	0	3	3	4
699	Casein kinase II subunit alpha OS=Mus musculus GN=Csnk2a1 PE=1 SV=2	Csnk2a1	45 kDa	0.03	KO Synap low, WT Synap high	0	0	0	1	2	4
700	3-hydroxyisobutyryl-CoA hydrolase, mitochondrial OS=Mus musculus GN=Hibch PE=1 SV=1	Hibch	43 kDa	0.019	KO Synap low, WT Synap high	0	0	0	2	1	3
701	Acyl-coenzyme A thioesterase 13 OS=Mus musculus GN=Acot13 PE=1 SV=1	Acot13	15 kDa	0.0085	KO Synap low, WT Synap high	0	0	0	1	1	3
702	Homer protein homolog 3 OS=Mus musculus GN=Homer3 PE=1 SV=2	Homer3	40 kDa	0.0073	KO Synap low, WT Synap high	0	1	1	3	2	4
703	Phospholemman OS=Mus musculus GN=Fxyd1 PE=1 SV=1	Fxyd1	8 kDa	0.014	KO Synap low, WT Synap high	1	1	1	1	1	1
704	Adenylate kinase 4, mitochondrial OS=Mus musculus GN=Ak4 PE=1 SV=1	Ak4	25 kDa	0.0043	KO Synap low, WT Synap high	0	0	0	3	2	3
705	NADH dehydrogenase [ubiquinone] flavoprotein 3, mitochondrial OS=Mus musculus GN=Ndufv3 PE=1 SV=1	Ndufv3	50 kDa	< 0.00010	KO Synap low, WT Synap high	0	0	0	3	2	3
706	Alpha-soluble NSF attachment protein OS=Mus musculus GN=Napa PE=1 SV=1	Napa	33 kDa	0.023	KO Synap low, WT Synap high	2	0	1	2	3	4
707	Guanine nucleotide-binding protein G(i) subunit alpha-1 OS=Mus musculus GN=Gnai1 PE=1 SV=1	Gnai1	40 kDa	0.0012	KO Synap low, WT Synap high	1	1	1	1	2	2
708	Intercellular adhesion molecule 5 OS=Mus musculus GN=Icam5 PE=1 SV=2	Icam5	97 kDa	0.00024	KO Synap low, WT Synap high	1	0	0	4	4	4
709	Heat shock protein 75 kDa, mitochondrial OS=Mus musculus GN=Trap1 PE=1 SV=1	Trap1	80 kDa	< 0.00010	KO Synap low, WT Synap high	0	0	0	3	3	4
710	Delta-1-pyrroline-5-carboxylate dehydrogenase, mitochondrial OS=Mus musculus GN=Aldh4a1 PE=1 SV=3	Aldh4a1	62 kDa	0.012	KO Synap low, WT Synap high	0	0	0	4	2	2

711	Thioredoxin, mitochondrial (Fragment) OS=Mus musculus GN=Txn2 PE=1 SV=1	Txn2	14 kDa	0.006	KO Synap low, WT Synap high	0	0	0	2	2	2
712	Neurocalcin-delta OS=Mus musculus GN=Ncald PE=1 SV=4	Ncald	22 kDa	0.011	KO Synap low, WT Synap high	0	0	0	1	2	3
713	Synapsin-3 OS=Mus musculus GN=Syn3 PE=1 SV=2	Syn3	63 kDa	0.016	KO Synap low, WT Synap high	0	2	0	4	2	2
714	Peptidyl-prolyl cis-trans isomerase D OS=Mus musculus GN=Ppid PE=1 SV=3	Ppid	41 kDa	0.0015	KO Synap low, WT Synap high	0	0	1	1	1	2
715	Voltage-dependent calcium channel subunit alpha-2/delta-1 OS=Mus musculus GN=Cacna2d1 PE=1 SV=1	Cacna2d1	125 kDa	0.0043	KO Synap low, WT Synap high	0	0	0	3	6	5
716	Ankyrin repeat and sterile alpha motif domain-containing protein 1B OS=Mus musculus GN=Anks1b PE=1 SV=1	Anks1b	48 kDa	0.00068	KO Synap low, WT Synap high	0	0	0	3	2	3
717	Glutamate decarboxylase 2 OS=Mus musculus GN=Gad2 PE=1 SV=1	Gad2	65 kDa	0.0012	KO Synap low, WT Synap high	0	0	0	3	2	3
718	Neuroligin-2 OS=Mus musculus GN=Nlgn2 PE=1 SV=2	Nlgn2	91 kDa	0.00023	KO Synap low, WT Synap high	1	1	1	1	3	3
719	SH3 and multiple ankyrin repeat domains protein 1 OS=Mus musculus GN=Shank1 PE=1 SV=1	Shank1	225 kDa	0.016	KO Synap low, WT Synap high	1	1	0	1	2	1
720	Histidine triad nucleotide-binding protein 2, mitochondrial OS=Mus musculus GN=Hint2 PE=1 SV=1	Hint2	17 kDa	0.024	KO Synap low, WT Synap high	0	0	0	1	3	1
721	Ras-related protein Rab-35 OS=Mus musculus GN=Rab35 PE=1 SV=1	Rab35	23 kDa	0.036	KO Synap low, WT Synap high	0	1	0	3	3	3
722	Ras-related protein Ral-A OS=Mus musculus GN=Rala PE=1 SV=1	Rala	24 kDa	0.0076	KO Synap low, WT Synap high	0	1	0	3	2	3
723	Sodium channel protein OS=Mus musculus GN=Scn2a PE=1 SV=1	Scn2a	228 kDa	0.00046	KO Synap low, WT Synap high	1	0	0	3	3	3
724	Glutaredoxin-related protein 5, mitochondrial OS=Mus musculus GN=Glrx5 PE=1 SV=2	Glrx5	16 kDa	0.00044	KO Synap low, WT Synap high	0	0	0	2	3	3
725	OCIA domain-containing protein 1 (Fragment) OS=Mus musculus GN=Ociad1 PE=1 SV=1	Ociad1	20 kDa	0.03	KO Synap low, WT Synap high	0	0	0	3	1	2
726	Lymphocyte antigen 6H OS=Mus musculus GN=Ly6h PE=1 SV=2	Ly6h	15 kDa	< 0.00010	KO Synap low, WT Synap high	0	0	0	2	1	2
727	Contactin-associated protein-like 2 OS=Mus musculus GN=Cntnap2 PE=1 SV=2	Cntnap2	148 kDa	< 0.00010	KO Synap low, WT Synap high	0	0	0	2	3	3
728	Guanine nucleotide-binding protein G(z) subunit alpha OS=Mus musculus GN=Gnaz PE=1 SV=4	Gnaz	41 kDa	0.019	KO Synap low, WT Synap high	0	2	1	2	2	2
729	Trans-2-enoyl-CoA reductase, mitochondrial OS=Mus musculus GN=Mecr PE=1 SV=2	Mecr	40 kDa	0.00063	KO Synap low, WT Synap high	0	0	0	2	2	3
730	MCG9827 OS=Mus musculus GN=Opcml PE=1 SV=1	Opcml	38 kDa	0.022	KO Synap low, WT Synap high	0	0	0	2	2	2
731	Voltage-dependent anion-selective channel protein 2 (Fragment) OS=Mus musculus GN=Vdac2 PE=1 SV=1	Vdac2	30 kDa	0.0045	KO Synap low, WT Synap high	0	0	1	2	3	3
732	Neuroligin-3 OS=Mus musculus GN=Nlgn3 PE=1 SV=1	Nlgn3	93 kDa	0.0059	KO Synap low, WT Synap high	0	0	0	3	2	2
733	Anion exchange protein OS=Mus musculus GN=Slc4a10 PE=1 SV=1	Slc4a10	125 kDa	0.0024	KO Synap low, WT Synap high	0	0	0	2	3	2
734	ATP-dependent Clp protease proteolytic subunit, mitochondrial OS=Mus musculus GN=Clpp PE=1 SV=1	Clpp	30 kDa	0.0007	KO Synap low, WT Synap high	0	0	0	2	3	2
735	Probable G-protein coupled receptor 158 OS=Mus musculus GN=Gpr158 PE=1 SV=2	Gpr158	134 kDa	< 0.00010	KO Synap low, WT Synap high	0	0	0	3	4	3
736	Spectrin beta 1 OS=Mus musculus GN=Sptb PE=1 SV=1	Sptb	268 kDa	0.042	KO Synap low, WT Synap high	1	1	1	3	1	4
737	Synaptotagmin-1 OS=Mus musculus GN=Syt1 PE=1 SV=1	Syt1	47 kDa	0.0015	KO Synap low, WT Synap high	0	0	1	3	3	2
738	2,4-dienoyl-CoA reductase, mitochondrial OS=Mus musculus GN=Decr1 PE=1 SV=1	Decr1	36 kDa	< 0.00010	KO Synap low, WT Synap high	0	0	0	2	2	2

739	Neutral amino acid transporter A OS=Mus musculus GN=Slc1a4 PE=1 SV=1	Slc1a4	56 kDa	0.0003	KO Synap low, WT Synap high	0	0	0	1	1	1
740	Toll-interacting protein OS=Mus musculus GN=Tollip PE=1 SV=1	Tollip	25 kDa	0.036	KO Synap low, WT Synap high	1	1	1	2	2	2
741	Programmed cell death protein 5 OS=Mus musculus GN=Pdcd5 PE=1 SV=3	Pdcd5	14 kDa	0.013	KO Synap low, WT Synap high	1	0	1	2	2	2
742	Guanine nucleotide-binding protein G(s) subunit alpha isoforms XLas OS=Mus musculus GN=Gnas PE=1 SV=1	Gnas	122 kDa	0.0071	KO Synap low, WT Synap high	1	0	1	1	1	1
743	Contactin-2 OS=Mus musculus GN=Cntn2 PE=1 SV=2	Cntn2	113 kDa	0.017	KO Synap low, WT Synap high	0	1	1	2	2	2
744	Adenosylhomocysteinase OS=Mus musculus GN=Ahcyl2 PE=1 SV=1	Ahcyl2	67 kDa	0.0009	KO Synap low, WT Synap high	1	1	2	2	2	3
745	Gamma-aminobutyric acid type B receptor subunit 2 OS=Mus musculus GN=Gabbr2 PE=1 SV=2	Gabbr2	106 kDa	0.0085	KO Synap low, WT Synap high	0	0	0	2	2	4
746	OX-2 membrane glycoprotein OS=Mus musculus GN=Cd200 PE=1 SV=1	Cd200	30 kDa	0.00063	KO Synap low, WT Synap high	0	0	0	2	2	3
747	ATP synthase subunit f, mitochondrial OS=Mus musculus GN=Atp5j2 PE=1 SV=3	Atp5j2	10 kDa	0.019	KO Synap low, WT Synap high	0	0	0	1	2	2
748	Cytochrome c oxidase subunit 5A, mitochondrial OS=Mus musculus GN=Cox5a PE=1 SV=2	Cox5a	16 kDa	< 0.00010	KO Synap low, WT Synap high	0	0	0	1	1	1
749	Mitochondrial import inner membrane translocase subunit TIM50 OS=Mus musculus GN=Timm50 PE=1 SV=1	Timm50	40 kDa	0.00058	KO Synap low, WT Synap high	0	0	0	3	3	3
750	Microtubule-actin cross-linking factor 1 OS=Mus musculus GN=Macf1 PE=1 SV=1	Macf1	608 kDa	0.0043	KO Synap low, WT Synap high	1	1	1	2	2	3
751	Contactin-associated protein 1 OS=Mus musculus GN=Cntnap1 PE=1 SV=2	Cntnap1	156 kDa	0.0027	KO Synap low, WT Synap high	0	0	0	2	3	4
752	Mitochondrial fission 1 protein OS=Mus musculus GN=Fis1 PE=1 SV=1	Fis1	17 kDa	0.048	KO Synap low, WT Synap high	0	1	0	2	2	1
753	Electron transfer flavoprotein-ubiquinone oxidoreductase, mitochondrial OS=Mus musculus GN=Etfdh PE=1 SV=1	Etfdh	68 kDa	0.011	KO Synap low, WT Synap high	0	0	0	4	2	3
754	Regulating synaptic membrane exocytosis protein 1 OS=Mus musculus GN=Rims1 PE=1 SV=2	Rims1	140 kDa	0.027	KO Synap low, WT Synap high	0	0	0	3	2	1
755	Protein Tmed7 OS=Mus musculus GN=Tmed7 PE=1 SV=1	Tmed7	25 kDa	0.0044	KO Synap low, WT Synap high	0	0	0	2	3	3
756	ATPase family AAA domain-containing protein 3 OS=Mus musculus GN=Atad3 PE=1 SV=1	Atad3	67 kDa	0.027	KO Synap low, WT Synap high	0	0	0	2	1	1
757	Hydroxyacyl-coenzyme A dehydrogenase, mitochondrial OS=Mus musculus GN=Hadh PE=1 SV=2	Hadh	34 kDa	0.0024	KO Synap low, WT Synap high	0	0	0	1	2	1
758	Metabotropic glutamate receptor 3 OS=Mus musculus GN=Grm3 PE=1 SV=1	Grm3	99 kDa	0.0021	KO Synap low, WT Synap high	0	0	0	3	2	4
759	Palmitoyl-protein thioesterase 1 OS=Mus musculus GN=Ppt1 PE=1 SV=2	Ppt1	34 kDa	0.00044	KO Synap low, WT Synap high	0	0	0	2	3	3
760	Citrate lyase subunit beta-like protein, mitochondrial OS=Mus musculus GN=Clybl PE=1 SV=2	Clybl	38 kDa	0.00063	KO Synap low, WT Synap high	0	0	0	2	2	2
761	Ribosome-recycling factor, mitochondrial OS=Mus musculus GN=Mrrf PE=1 SV=1	Mrrf	29 kDa	0.00063	KO Synap low, WT Synap high	0	0	0	2	2	3
762	Cytochrome b-c1 complex subunit 9 OS=Mus musculus GN=Uqcr10 PE=1 SV=1	Uqcr10	7 kDa	0.024	KO Synap low, WT Synap high	0	0	0	1	1	1
763	Dolichyl-diphosphooligosaccharide--protein glycosyltransferase subunit 1 OS=Mus musculus GN=Rpn1 PE=1 SV=1	Rpn1	69 kDa	0.02	KO Synap low, WT Synap high	0	0	1	2	2	2
764	Glutamate receptor 3 OS=Mus musculus GN=Gria3 PE=1 SV=1	Gria3	100 kDa	0.0024	KO Synap low, WT Synap high	0	0	0	2	2	2
765	Mitochondrial import inner membrane translocase subunit Tim13 OS=Mus musculus GN=Timm13 PE=1 SV=1	Timm13	10 kDa	0.0044	KO Synap low, WT Synap high	0	0	0	1	1	2
766	L-2-hydroxyglutarate dehydrogenase, mitochondrial OS=Mus musculus GN=L2hgdh PE=1 SV=1	L2hgdh	51 kDa	0.0019	KO Synap low, WT Synap high	0	0	0	2	2	3

767	Prohibitin OS=Mus musculus GN=Phb PE=1 SV=1	Phb	30 kDa	< 0.00010	KO Synap low, WT Synap high	0	0	0	1	1	1
768	NADH dehydrogenase [ubiquinone] iron-sulfur protein 4, mitochondrial OS=Mus musculus GN=Ndufs4 PE=1 SV=1	Ndufs4	20 kDa	0.0085	KO Synap low, WT Synap high	0	0	0	2	1	2
769	Large neutral amino acids transporter small subunit 1 OS=Mus musculus GN=Slc7a5 PE=1 SV=2	Slc7a5	56 kDa	0.00063	KO Synap low, WT Synap high	0	0	0	1	1	1
770	Neuroligin 4-like OS=Mus musculus GN=Nlgn4l PE=1 SV=1	Nlgn4l	97 kDa	0.0003	KO Synap low, WT Synap high	1	1	1	1	1	1
771	Serine/threonine-protein kinase BRSK1 OS=Mus musculus GN=Brsk1 PE=1 SV=1	Brsk1	85 kDa	0.016	KO Synap low, WT Synap high	0	0	0	1	2	3
772	Actin-related protein 2/3 complex subunit 3 OS=Mus musculus GN=Arpc3 PE=1 SV=3	Arpc3	21 kDa	0.0085	KO Synap low, WT Synap high	0	0	0	1	1	2
773	Mitofusin-2 OS=Mus musculus GN=Mfn2 PE=1 SV=3	Mfn2	86 kDa	0.016	KO Synap low, WT Synap high	0	0	0	1	2	3
774	Cysteine desulfurase, mitochondrial OS=Mus musculus GN=Nfs1 PE=1 SV=3	Nfs1	51 kDa	0.049	KO Synap low, WT Synap high	0	0	0	1	1	2
775	ARF6 guanine nucleotide exchange factor IQArfGEF OS=Mus musculus GN=Iqsec2 PE=1 SV=1	Iqsec2	162 kDa	0.027	KO Synap low, WT Synap high	0	0	0	3	2	2
776	MICOS complex subunit MIC60 OS=Mus musculus GN=Immt PE=1 SV=1	Immt	54 kDa	0.00019	KO Synap low, WT Synap high	0	0	0	1	1	1
777	Glutamate receptor 2 OS=Mus musculus GN=Gria2 PE=1 SV=1	Gria2	99 kDa	0.0023	KO Synap low, WT Synap high	0	0	0	1	1	1
778	NADH dehydrogenase [ubiquinone] iron-sulfur protein 5 OS=Mus musculus GN=Ndufs5 PE=1 SV=3	Ndufs5	13 kDa	0.0027	KO Synap low, WT Synap high	0	0	0	2	3	3
779	Endophilin-A3 OS=Mus musculus GN=Sh3gl3 PE=1 SV=1	Sh3gl3	39 kDa	0.027	KO Synap low, WT Synap high	0	0	0	2	1	1
780	Potassium voltage-gated channel subfamily A member 1 OS=Mus musculus GN=Kcna1 PE=1 SV=1	Kcna1	56 kDa	0.012	KO Synap low, WT Synap high	1	0	1	1	2	2
781	Pyridoxal phosphate phosphatase OS=Mus musculus GN=Pdxp PE=1 SV=1	Pdxp	32 kDa	0.0044	KO Synap low, WT Synap high	0	0	0	1	2	2
782	Up-regulated during skeletal muscle growth protein 5 OS=Mus musculus GN=Usmg5 PE=1 SV=1	Usmg5	6 kDa	0.0024	KO Synap low, WT Synap high	0	0	0	2	2	2
783	Monoacylglycerol lipase ABHD12 OS=Mus musculus GN=Abhd12 PE=1 SV=2	Abhd12	45 kDa	0.0074	KO Synap low, WT Synap high	0	0	1	1	2	2
784	Catechol O-methyltransferase domain-containing protein 1 OS=Mus musculus GN=Comtd1 PE=1 SV=1	Comtd1	29 kDa	0.049	KO Synap low, WT Synap high	0	0	0	1	1	3
785	Arfaptin-2 OS=Mus musculus GN=Arfip2 PE=1 SV=2	Arfip2	38 kDa	0.032	KO Synap low, WT Synap high	0	1	0	1	2	1
786	Serine/threonine-protein kinase MARK2 (Fragment) OS=Mus musculus GN=Mark2 PE=1 SV=1	Mark2	87 kDa	0.019	KO Synap low, WT Synap high	0	0	0	2	1	3
787	MICOS complex subunit (Fragment) OS=Mus musculus GN=Chchd3 PE=1 SV=1	Chchd3	22 kDa	0.00038	KO Synap low, WT Synap high	0	1	0	1	1	1
788	Leucine-rich repeat-containing protein 7 OS=Mus musculus GN=Lrrc7 PE=1 SV=1	Lrrc7	168 kDa	0.0087	KO Synap low, WT Synap high	0	0	0	1	1	2
789	Long-chain-fatty-acid--CoA ligase 1 OS=Mus musculus GN=Acsl1 PE=1 SV=2	Acsl1	78 kDa	0.0085	KO Synap low, WT Synap high	0	0	0	1	1	2
790	GTPase NRas (Fragment) OS=Mus musculus GN=Nras PE=1 SV=1	Nras	21 kDa	0.00022	KO Synap low, WT Synap high	0	0	0	1	1	1
791	Thioredoxin-related transmembrane protein 2 OS=Mus musculus GN=Tmx2 PE=1 SV=1	Tmx2	30 kDa	0.024	KO Synap low, WT Synap high	0	0	0	1	2	2
792	ATPase family AAA domain-containing protein 1 OS=Mus musculus GN=Atad1 PE=1 SV=1	Atad1	41 kDa	0.027	KO Synap low, WT Synap high	0	0	0	2	1	1
793	Calcium-transporting ATPase OS=Mus musculus GN=Atp2b2 PE=1 SV=1	Atp2b2	127 kDa	< 0.00010	KO Synap low, WT Synap high	1	1	1	1	1	1
794	WD repeat-containing protein 37 OS=Mus musculus GN=Wdr37 PE=1 SV=1	Wdr37	55 kDa	0.016	KO Synap low, WT Synap high	0	0	0	1	1	1

795	Cytochrome b5 OS=Mus musculus GN=Cyb5a PE=1 SV=2	Cyb5a	15 kDa	0.0085	KO Synap low, WT Synap high	0	0	0	1	1	2
796	Regulating synaptic membrane exocytosis protein 1 (Fragment) OS=Mus musculus GN=Rims1 PE=1 SV=3	Rims1	89 kDa	0.0014	KO Synap low, WT Synap high	0	0	0	1	1	1
797	Peptidyl-prolyl cis-trans isomerase FKBP8 OS=Mus musculus GN=Fkbp8 PE=1 SV=2	Fkbp8	44 kDa	0.027	KO Synap low, WT Synap high	0	0	0	2	1	1
798	Radixin OS=Mus musculus GN=Rdx PE=1 SV=3	Rdx	69 kDa	0.0085	KO Synap low, WT Synap high	0	0	0	1	1	1
799	Leucine-rich repeat and immunoglobulin-like domain-containing nogo receptor-interacting protein 1 OS=Mus musculus GN=Lingo1 PE=1 SV=1	Lingo1	70 kDa	0.016	KO Synap low, WT Synap high	0	0	0	1	1	1
800	Metabotropic glutamate receptor 2 OS=Mus musculus GN=Grm2 PE=1 SV=2	Grm2	96 kDa	< 0.00010	KO Synap low, WT Synap high	0	0	0	2	2	2
801	Aspartyl/asparaginyl beta-hydroxylase OS=Mus musculus GN=Asph PE=1 SV=1	Asph	26 kDa	0.0085	KO Synap low, WT Synap high	0	0	0	1	1	2
802	Dehydrogenase/reductase SDR family member 1 OS=Mus musculus GN=Dhrs1 PE=1 SV=1	Dhrs1	34 kDa	0.028	KO Synap low, WT Synap high	0	0	0	2	3	1
803	Ras-related protein Rab-3B OS=Mus musculus GN=Rab3b PE=1 SV=1	Rab3b	25 kDa	0.0058	KO Synap low, WT Synap high	0	0	0	1	1	1
804	Phosphatidylinositol 4-kinase alpha OS=Mus musculus GN=Pi4ka PE=1 SV=2	Pi4ka	237 kDa	0.028	KO Synap low, WT Synap high	0	0	0	2	3	1
805	Apoptosis-inducing factor 1, mitochondrial OS=Mus musculus GN=Aifm1 PE=1 SV=1	Aifm1	67 kDa	< 0.00010	KO Synap low, WT Synap high	0	0	0	1	1	1
806	Synaptogyrin-1 OS=Mus musculus GN=Syngr1 PE=1 SV=2	Syngr1	26 kDa	< 0.00010	KO Synap low, WT Synap high	0	0	0	1	1	1
807	Bcl-2-like protein 13 OS=Mus musculus GN=Bcl2l13 PE=1 SV=2	Bcl2l13	47 kDa	< 0.00010	KO Synap low, WT Synap high	0	0	0	1	1	1
808	Versican core protein OS=Mus musculus GN=Vcan PE=1 SV=2	Vcan	367 kDa	0.027	KO Synap low, WT Synap high	0	0	0	2	1	1
809	Tricarboxylate transport protein, mitochondrial OS=Mus musculus GN=Slc25a1 PE=1 SV=1	Slc25a1	34 kDa	< 0.00010	KO Synap low, WT Synap high	0	0	0	2	2	2
810	28S ribosomal protein S28, mitochondrial OS=Mus musculus GN=Mrps28 PE=1 SV=1	Mrps28	21 kDa	0.037	KO Synap low, WT Synap high	0	0	0	2	2	1
811	Non-specific lipid-transfer protein OS=Mus musculus GN=Scp2 PE=1 SV=3	Scp2	59 kDa	< 0.00010	KO Synap low, WT Synap high	0	0	0	1	1	1
812	Ras-related protein Rab-33B OS=Mus musculus GN=Rab33b PE=1 SV=1	Rab33b	26 kDa	0.00081	KO Synap low, WT Synap high	0	0	0	1	1	1
813	Receptor-type tyrosine-protein phosphatase alpha OS=Mus musculus GN=Ptpra PE=1 SV=3	Ptpra	94 kDa	< 0.00010	KO Synap low, WT Synap high	0	0	0	1	1	1
814	Acyl-coenzyme A thioesterase THEM4 OS=Mus musculus GN=Them4 PE=1 SV=1	Them4	26 kDa	< 0.00010	KO Synap low, WT Synap high	0	0	0	1	1	1
815	Serine/threonine-protein phosphatase 2A catalytic subunit alpha isoform OS=Mus musculus GN=Ppp2ca PE=1 SV=1	Ppp2ca	36 kDa	0.0085	KO Synap low, WT Synap high	0	0	0	1	1	2
816	Voltage-dependent calcium channel subunit alpha-2/delta-2 OS=Mus musculus GN=Cacna2d2 PE=1 SV=2	Cacna2d2	131 kDa	0.027	KO Synap low, WT Synap high	0	0	0	2	1	1
817	UPF0598 protein C8orf82 homolog OS=Mus musculus PE=1 SV=1		24 kDa	< 0.00010	KO Synap low, WT Synap high	0	0	0	1	1	1
818	Tumor protein D52 (Fragment) OS=Mus musculus GN=Tpd52 PE=1 SV=1	Tpd52	19 kDa	< 0.00010	KO Synap low, WT Synap high	0	0	0	1	1	1
819	Neuronal proto-oncogene tyrosine-protein kinase Src OS=Mus musculus GN=Src PE=1 SV=1	Src	60 kDa	< 0.00010	KO Synap low, WT Synap high	0	0	0	1	1	1
820	Hydroxymethylglutaryl-CoA lyase, mitochondrial OS=Mus musculus GN=Hmgcl PE=1 SV=2	Hmgcl	34 kDa	< 0.00010	KO Synap low, WT Synap high	0	0	0	1	1	1
821	Vacuolar protein sorting-associated protein 51 homolog OS=Mus musculus GN=Vps51 PE=1 SV=2	Vps51	86 kDa	< 0.00010	KO Synap low, WT Synap high	0	0	0	1	1	1
822	Thioredoxin OS=Mus musculus GN=Txn PE=1 SV=3	Txn	12 kDa	0.0085	KO Synap low, WT Synap high	0	0	0	1	1	2

823	Cathepsin L1 OS=Mus musculus GN=Ctsl PE=1 SV=2	Ctsl	38 kDa	0.0087	KO Synap low, WT Synap high	0	0	0	1	1	1
824	Protein phosphatase 1 regulatory subunit 21 OS=Mus musculus GN=Ppp1r21 PE=1 SV=2	Ppp1r21	88 kDa	< 0.00010	KO Synap low, WT Synap high	0	0	0	1	1	1
825	CD166 antigen (Fragment) OS=Mus musculus GN=Alcam PE=1 SV=1	Alcam	38 kDa	< 0.00010	KO Synap low, WT Synap high	0	0	0	1	1	1
826	Actin-related protein 3B OS=Mus musculus GN=Actr3b PE=1 SV=1	Actr3b	48 kDa	0.024	KO Synap low, WT Synap high	0	0	0	1	1	1
827	Ras-related protein Rab-8A OS=Mus musculus GN=Rab8a PE=1 SV=2	Rab8a	24 kDa	0.00057	KO Synap low, WT Synap high	0	0	0	1	1	1
828	SH2 domain-containing adapter protein F (Fragment) OS=Mus musculus GN=Shf PE=1 SV=1	Shf	47 kDa	< 0.00010	KO Synap low, WT Synap high	0	0	0	1	1	1
829	Methionine--tRNA ligase, mitochondrial OS=Mus musculus GN=Mars2 PE=1 SV=2	Mars2	66 kDa	< 0.00010	KO Synap low, WT Synap high	0	0	0	1	1	1

Supplementary table S4: Non-altered proteins in synaptosomes from WT and MPSIIIC mice.

			Exclusive Unique Peptide Count					
			KO Synap	KO Synap	KO Synap	WT Synap	WT Synap	WT Synap
Identified Proteins (2313)	Alternate ID	ANOVA Test (p-value): (p < 0.05)	KO S4	KO S5	KO S6	WT S4	WT S5	WT S6
Dynamin-1 OS=Mus musculus GN=Dnm1 PE=1 SV=1	Dnm1	0.31	43	41	33	53	53	54
Glutamine synthetase OS=Mus musculus GN=Glul PE=1 SV=6	Glul	0.3	35	33	41	32	33	29
Phosphoglycerate kinase 1 OS=Mus musculus GN=Pgk1 PE=1 SV=4	Pgk1	0.61	27	29	29	21	21	21
Myelin basic protein (Fragment) OS=Mus musculus GN=Mbp PE=1 SV=1	Mbp	0.06	20	0	0	18	21	23
Triosephosphate isomerase OS=Mus musculus GN=Tpi1 PE=1 SV=4	Tpi1	0.22	21	23	28	17	18	19
Cytoplasmic dynein 1 heavy chain 1 OS=Mus musculus GN=Dync1h1 PE=1 SV=2	Dync1h1	0.36	42	41	29	25	35	41
Spectrin beta chain, non-erythrocytic 1 OS=Mus musculus GN=Sptbn1 PE=1 SV=2	Sptbn1	0.64	26	22	22	3	43	49
Ubiquitin carboxyl-terminal hydrolase isozyme L1 OS=Mus musculus GN=Uchl1 PE=1 SV=1	Uchl1	0.087	15	13	16	12	13	12
V-type proton ATPase subunit B, brain isoform OS=Mus musculus GN=Atp6v1b2 PE=1 SV=1	Atp6v1b2	0.067	19	14	13	13	14	15
Microtubule-associated protein OS=Mus musculus GN=Mapt PE=1 SV=1	Mapt	0.086	20	20	15	19	17	20
Glycogen phosphorylase, brain form OS=Mus musculus GN=Pygb PE=1 SV=3	Pygb	0.33	21	19	29	17	16	15
Plasma membrane calcium-transporting ATPase 2 OS=Mus musculus GN=Atp2b2 PE=1 SV=2	Atp2b2	0.069	0	19	17	30	0	34
Protein Sptbn2 OS=Mus musculus GN=Sptbn2 PE=1 SV=1	Sptbn2	0.29	35	32	20	17	19	20
Peroxiredoxin 6 OS=Mus musculus GN=Prdx6 PE=1 SV=1	Prdx6	0.14	17	13	17	12	9	11
AP2-associated protein kinase 1 OS=Mus musculus GN=Aak1 PE=1 SV=2	Aak1	0.056	13	13	15	12	14	15
Serine/threonine-protein phosphatase 2B catalytic subunit alpha isoform OS=Mus musculus GN=Ppp3ca PE=1 SV=1	Ppp3ca	0.5	13	8	11	8	7	12
Beta-synuclein OS=Mus musculus GN=Sncb PE=1 SV=1	Sncb	0.63	10	12	10	14	7	6
Protein NDRG2 OS=Mus musculus GN=Ndrg2 PE=1 SV=1	Ndrg2	0.79	13	12	17	9	10	10

14-3-3 protein theta OS=Mus musculus GN=Ywhaq PE=1 SV=1	Ywhaq	0.23	9	12	12	8	10	12
Gamma-enolase OS=Mus musculus GN=Eno2 PE=1 SV=2	Eno2	0.12	16	12	17	8	8	9
Calcium-dependent secretion activator 1 OS=Mus musculus GN=Cadps PE=1 SV=3	Cadps	0.061	13	15	11	13	13	16
Cytosolic acyl coenzyme A thioester hydrolase OS=Mus musculus GN=Acot7 PE=1 SV=2	Acot7	0.19	12	10	15	8	8	7
Synaptojanin-1 OS=Mus musculus GN=Synj1 PE=1 SV=1	Synj1	0.23	24	19	17	10	14	0
Reticulon-4 OS=Mus musculus GN=Rtn4 PE=1 SV=2	Rtn4	0.4	13	13	14	13	8	11
78 kDa glucose-regulated protein OS=Mus musculus GN=Hspa5 PE=1 SV=3	Hspa5	0.12	17	13	17	13	13	12
V-type proton ATPase subunit H OS=Mus musculus GN=Atp6v1h PE=1 SV=1	Atp6v1h	0.31	10	9	9	9	8	9
Oxidation resistance protein 1 OS=Mus musculus GN=Oxr1 PE=1 SV=3	Oxr1	0.45	15	15	10	15	14	12
Complexin-1 OS=Mus musculus GN=Cplx1 PE=1 SV=1	Cplx1	0.46	5	6	3	4	4	4
14-3-3 protein eta OS=Mus musculus GN=Ywhah PE=1 SV=2	Ywhah	0.053	9	8	10	9	9	11
ADP-ribosylation factor 3 OS=Mus musculus GN=Arf3 PE=2 SV=2	Arf3	0.12	9	8	9	5	5	9
ATP-dependent 6-phosphofructokinase, platelet type OS=Mus musculus GN=Pfkp PE=1 SV=1	Pfkp	0.071	6	9	10	10	10	10
F-actin-capping protein subunit alpha-2 OS=Mus musculus GN=Capza2 PE=1 SV=3	Capza2	0.1	8	7	8	5	5	5
T-complex protein 1 subunit theta OS=Mus musculus GN=Cct8 PE=1 SV=3	Cct8	0.096	12	12	10	7	7	10
Microtubule-associated protein 4 OS=Mus musculus GN=Map4 PE=1 SV=3	Map4	0.21	13	11	8	12	11	9
Fascin OS=Mus musculus GN=Fscn1 PE=1 SV=4	Fscn1	0.055	9	9	10	6	7	9
T-complex protein 1 subunit alpha OS=Mus musculus GN=Tcp1 PE=1 SV=3	Tcp1	0.97	10	11	11	12	10	7
T-complex protein 1 subunit gamma OS=Mus musculus GN=Cct3 PE=1 SV=1	Cct3	0.79	12	12	13	8	12	10
Myristoylated alanine-rich C-kinase substrate OS=Mus musculus GN=Marcks PE=1 SV=2	Marcks	0.72	5	5	5	5	4	9
T-complex protein 1 subunit beta OS=Mus musculus GN=Cct2 PE=1 SV=4	Cct2	0.21	14	11	9	10	8	6
Ras-related protein Rab-14 OS=Mus musculus GN=Rab14 PE=1 SV=3	Rab14	0.15	6	6	5	7	5	7
Kinesin heavy chain isoform 5C OS=Mus musculus GN=Kif5c PE=1 SV=3	Kif5c	0.96	15	9	6	8	9	9
Coronin-1A OS=Mus musculus GN=Coro1a PE=1 SV=5	Coro1a	0.34	6	8	9	9	11	9
Reticulon-3 OS=Mus musculus GN=Rtn3 PE=1 SV=2	Rtn3	0.98	8	10	9	7	7	4
Reticulon-1 OS=Mus musculus GN=Rtn1 PE=1 SV=1	Rtn1	0.069	6	8	7	6	8	6
Calreticulin OS=Mus musculus GN=Calr PE=1 SV=1	Calr	0.29	9	8	14	7	6	6
Rho GDP-dissociation inhibitor 1 OS=Mus musculus GN=Arhgdia PE=1 SV=3	Arhgdia	0.066	4	4	5	2	2	2
Phosphatidylethanolamine-binding protein 1 OS=Mus musculus GN=Pebp1 PE=1 SV=3	Pebp1	0.28	5	7	9	4	4	6
MCG124046 OS=Mus musculus GN=Prss1 PE=1 SV=1	Prss1	0.11	2	2	4	1	1	1

Dynactin subunit 1 OS=Mus musculus GN=Dctn1 PE=1 SV=3	Dctn1	0.061	11	11	9	4	6	7
Endoplasmin OS=Mus musculus GN=Hsp90b1 PE=1 SV=2	Hsp90b1	0.45	6	9	10	9	3	6
Ras-related protein Rab-2A OS=Mus musculus GN=Rab2a PE=1 SV=1	Rab2a	0.79	8	6	6	6	5	6
Protein RUFY3 OS=Mus musculus GN=Rufy3 PE=1 SV=1	Rufy3	0.14	12	15	8	5	5	5
Gamma-soluble NSF attachment protein OS=Mus musculus GN=Napg PE=1 SV=1	Napg	0.36	9	7	5	7	10	8
Dihydropteridine reductase OS=Mus musculus GN=Qdpr PE=1 SV=1	Qdpr	0.13	8	0	6	2	3	6
Cullin-associated NEDD8-dissociated protein 1 OS=Mus musculus GN=Cand1 PE=1 SV=2	Cand1	0.11	9	9	8	4	9	6
Calmodulin OS=Mus musculus GN=Calm1 PE=1 SV=2	Calm1	0.22	7	6	6	3	5	2
Secretory carrier-associated membrane protein 1 OS=Mus musculus GN=Scamp1 PE=1 SV=1	Scamp1	0.16	5	7	4	7	4	4
T-complex protein 1 subunit zeta OS=Mus musculus GN=Cct6a PE=1 SV=3	Cct6a	0.053	7	8	7	3	3	6
CaM kinase-like vesicle-associated protein OS=Mus musculus GN=Camkv PE=1 SV=2	Camkv	0.35	5	6	12	4	6	8
V-type proton ATPase subunit C 1 OS=Mus musculus GN=Atp6v1c1 PE=1 SV=4	Atp6v1c1	0.14	5	7	4	3	3	4
Tenascin-R OS=Mus musculus GN=Tnr PE=1 SV=2	Tnr	0.062	5	5	5	5	5	6
Neurofilament 3, medium OS=Mus musculus GN=Nefm PE=1 SV=1	Nefm	0.15	6	2	3	8	6	6
Microtubule-associated protein RP/EB family member 3 OS=Mus musculus GN=Mapre3 PE=1 SV=1	Mapre3	0.8	3	6	3	4	4	4
PH and SEC7 domain-containing protein 3 OS=Mus musculus GN=Psd3 PE=1 SV=1	Psd3	0.57	6	7	3	9	6	5
Cortactin, isoform CRA_a OS=Mus musculus GN=Cttn PE=1 SV=1	Cttn	0.14	8	6	5	6	7	5
Phosphoglucomutase-1 OS=Mus musculus GN=Pgm1 PE=1 SV=4	Pgm1	0.97	5	4	8	0	5	8
Puromycin-sensitive aminopeptidase OS=Mus musculus GN=Npepps PE=1 SV=2	Npepps	0.65	4	6	10	1	5	7
Glycogen phosphorylase, muscle form OS=Mus musculus GN=Pygm PE=1 SV=3	Pygm	0.23	5	8	7	5	7	9
Kinesin-like protein KIF21A OS=Mus musculus GN=Kif21a PE=1 SV=1	Kif21a	0.06	6	6	5	4	4	4
Neurabin-2 OS=Mus musculus GN=Ppp1r9b PE=1 SV=1	Ppp1r9b	1	8	7	3	6	4	5
T-complex protein 1 subunit eta OS=Mus musculus GN=Cct7 PE=1 SV=1	Cct7	0.075	5	7	6	4	4	6
14-3-3 protein gamma OS=Mus musculus GN=Ywhag PE=1 SV=2	Ywhag	0.14	3	3	4	4	7	6
Na(+)/H(+) exchange regulatory cofactor NHE-RF1 OS=Mus musculus GN=Slc9a3r1 PE=1 SV=3	Slc9a3r1	0.076	5	3	4	4	6	4
Nucleoside diphosphate kinase A OS=Mus musculus GN=Nme1 PE=1 SV=1	Nme1	0.19	0	2	3	3	5	3
Neurogranin OS=Mus musculus GN=Nrgn PE=1 SV=1	Nrgn	0.29	4	3	3	3	2	2
CB1 cannabinoid receptor-interacting protein 1 OS=Mus musculus GN=Cnrip1 PE=1 SV=1	Cnrip1	0.069	4	4	3	3	4	0
Anion exchange protein OS=Mus musculus GN=Slc4a4 PE=1 SV=1	Slc4a4	0.53	3	3	4	4	6	0
Cell division control protein 42 homolog OS=Mus musculus GN=Cdc42 PE=1 SV=2	Cdc42	0.18	4	4	6	2	3	3

Gap junction alpha-1 protein OS=Mus musculus GN=Gja1 PE=1 SV=2	Gja1	0.13	4	4	3	7	5	4
Nck-associated protein 1 OS=Mus musculus GN=Nckap1 PE=1 SV=1	Nckap1	0.42	5	5	3	6	5	4
Ras-related protein Rab-7a OS=Mus musculus GN=Rab7a PE=1 SV=2	Rab7a	0.57	5	4	4	4	3	3
Importin subunit beta-1 OS=Mus musculus GN=Kpnb1 PE=1 SV=2	Kpnb1	0.11	7	7	5	2	3	3
Serine/threonine-protein phosphatase OS=Mus musculus GN=Ppp3cb PE=1 SV=1	Ppp3cb	0.14	5	4	3	3	5	4
Phosphatidylinositol 4-phosphate 5-kinase type-1 gamma OS=Mus musculus GN=Pip5k1c PE=1 SV=1	Pip5k1c	0.096	6	2	3	4	5	8
Ras-related protein Rab-6A OS=Mus musculus GN=Rab6a PE=1 SV=4	Rab6a	0.18	3	4	4	2	0	3
Serine/threonine-protein phosphatase 2A 55 kDa regulatory subunit B alpha isoform OS=Mus musculus GN=Ppp2r2a PE=1 SV=1	Ppp2r2a	0.12	7	6	6	2	3	4
Elongation factor 1-delta OS=Mus musculus GN=Eef1d PE=1 SV=1	Eef1d	0.93	4	3	3	3	5	4
ATP-dependent 6-phosphofructokinase, liver type OS=Mus musculus GN=Pfkl PE=1 SV=4	Pfkl	0.55	4	4	5	1	1	2
Actin-related protein 2/3 complex subunit 4 OS=Mus musculus GN=Arpc4 PE=1 SV=3	Arpc4	0.23	2	4	4	1	5	3
Serine racemase OS=Mus musculus GN=Srr PE=1 SV=1	Srr	0.48	3	4	4	3	4	3
Carbonyl reductase [NADPH] 1 OS=Mus musculus GN=Cbr1 PE=1 SV=3	Cbr1	0.058	2	3	4	2	3	2
Serine/threonine-protein kinase DCLK1 OS=Mus musculus GN=Dclk1 PE=1 SV=1	Dclk1	0.11	9	8	6	2	4	2
Guanine nucleotide-binding protein subunit beta-5 OS=Mus musculus GN=Gnb5 PE=1 SV=1	Gnb5	0.24	4	4	2	0	3	3
Liprin-alpha-3 OS=Mus musculus GN=Ppfia3 PE=1 SV=2	Ppfia3	0.19	5	4	0	4	3	4
Ras-related protein Rab-3C OS=Mus musculus GN=Rab3c PE=1 SV=1	Rab3c	0.067	1	1	2	2	3	5
Serine/threonine-protein phosphatase 5 OS=Mus musculus GN=Ppp5c PE=1 SV=3	Ppp5c	0.08	7	6	7	1	2	2
EF-hand domain-containing protein D2 OS=Mus musculus GN=Efhd2 PE=1 SV=1	Efhd2	0.41	3	5	3	3	3	4
Protein disulfide-isomerase A6 OS=Mus musculus GN=Pdia6 PE=1 SV=1	Pdia6	0.093	4	4	3	5	3	4
Dynamin-3 OS=Mus musculus GN=Dnm3 PE=1 SV=1	Dnm3	0.19	1	2	2	3	4	3
60S acidic ribosomal protein P2 OS=Mus musculus GN=Rplp2 PE=1 SV=3	Rplp2	0.57	2	6	0	5	4	3
Charged multivesicular body protein 4b OS=Mus musculus GN=Chmp4b PE=1 SV=2	Chmp4b	0.29	4	5	3	3	2	2
V-type proton ATPase subunit G 1 OS=Mus musculus GN=Atp6v1g1 PE=1 SV=3	Atp6v1g1	0.67	3	3	3	3	2	3
Cytoplasmic dynein 1 light intermediate chain 1 OS=Mus musculus GN=Dync1li1 PE=1 SV=1	Dync1li1	0.16	4	4	3	3	2	3
cAMP-dependent protein kinase type II-alpha regulatory subunit OS=Mus musculus GN=Prkar2a PE=1 SV=2	Prkar2a	0.15	3	5	7	4	3	2
Actin-related protein 2/3 complex subunit 1A OS=Mus musculus GN=Arpc1a PE=1 SV=1	Arpc1a	0.16	0	5	8	0	0	5
Ras-related protein Rab-5C OS=Mus musculus GN=Rab5c PE=1 SV=1	Rab5c	0.097	3	4	4	3	2	2
Glycerol-3-phosphate dehydrogenase 1-like protein OS=Mus musculus GN=Gpd1l PE=1 SV=2	Gpd1l	0.84	5	0	7	3	2	4
Tropomodulin-2 OS=Mus musculus GN=Tmod2 PE=1 SV=2	Tmod2	0.17	2	4	4	0	2	4

Protein phosphatase 1H OS=Mus musculus GN=Ppm1h PE=1 SV=1	Ppm1h	0.18	1	3	2	3	3	3
Transcriptional activator protein Pur-beta OS=Mus musculus GN=Purb PE=1 SV=3	Purb	0.2	3	5	4	3	3	3
3'(2'),5'-bisphosphate nucleotidase 1 OS=Mus musculus GN=Bpnt1 PE=1 SV=1	Bpnt1	0.75	5	1	4	3	5	0
C-1-tetrahydrofolate synthase, cytoplasmic OS=Mus musculus GN=Mthfd1 PE=1 SV=4	Mthfd1	0.077	5	4	6	4	3	3
Fatty acid-binding protein, brain OS=Mus musculus GN=Fabp7 PE=1 SV=1	Fabp7	0.36	3	3	3	2	2	1
CLIP-associating protein 2 OS=Mus musculus GN=Clasp2 PE=1 SV=1	Clasp2	0.25	6	5	1	2	3	2
Ras-related protein Rab-10 OS=Mus musculus GN=Rab10 PE=1 SV=1	Rab10	0.2	1	2	2	2	2	2
Ras-related protein Rab-11B OS=Mus musculus GN=Rab11b PE=1 SV=3	Rab11b	0.25	3	3	2	2	1	3
Cytoplasmic dynein 1 light intermediate chain 2 OS=Mus musculus GN=Dync1li2 PE=1 SV=2	Dync1li2	0.33	5	4	3	0	4	3
FERM, RhoGEF and pleckstrin domain-containing protein 1 OS=Mus musculus GN=Farp1 PE=1 SV=1	Farp1	0.15	3	2	3	4	4	2
Cysteine and glycine-rich protein 1 OS=Mus musculus GN=Csrp1 PE=1 SV=3	Csrp1	0.053	0	0	2	4	3	3
60S ribosomal protein L35 OS=Mus musculus GN=Rpl35 PE=1 SV=1	Rpl35	0.065	5	3	5	2	0	2
MCG18019 OS=Mus musculus GN=Vgf PE=1 SV=1	Vgf	0.11	2	3	4	5	4	3
Ras-related protein Rab-5A OS=Mus musculus GN=Rab5a PE=1 SV=1	Rab5a	0.063	4	3	1	2	2	1
Calnexin OS=Mus musculus GN=Canx PE=1 SV=1	Canx	0.8	4	3	0	4	0	0
Glutathione S-transferase (Fragment) OS=Mus musculus GN=Gstm5 PE=1 SV=1	Gstm5	0.1	2	3	2	1	1	1
Astrocytic phosphoprotein PEA-15 OS=Mus musculus GN=Pea15 PE=1 SV=1	Pea15	0.12	2	2	2	0	1	1
Hepatocyte cell adhesion molecule OS=Mus musculus GN=Hepacam PE=1 SV=2	Hepacam	0.16	2	0	2	2	2	2
Microtubule-associated protein OS=Mus musculus GN=Mapt PE=1 SV=1	Mapt	0.062	0	2	2	1	1	1
Calcium/calmodulin-dependent protein kinase type II subunit delta OS=Mus musculus GN=Camk2d PE=1 SV=1	Camk2d	0.28	3	3	0	2	2	3
F-box only protein 2 OS=Mus musculus GN=Fbxo2 PE=1 SV=1	Fbxo2	0.56	2	2	2	1	1	1
Programmed cell death 6-interacting protein OS=Mus musculus GN=Pdcd6ip PE=1 SV=3	Pdcd6ip	0.89	4	4	2	0	5	2
Glyoxalase domain-containing protein 4 OS=Mus musculus GN=Glod4 PE=1 SV=1	Glod4	0.092	2	3	3	0	0	4
Carboxypeptidase E OS=Mus musculus GN=Cpe PE=1 SV=2	Cpe	0.36	3	4	4	2	1	0
Prostaglandin E synthase 3 OS=Mus musculus GN=Ptges3 PE=1 SV=1	Ptges3	0.77	4	2	3	2	2	2
Sorting nexin-27 OS=Mus musculus GN=Snx27 PE=1 SV=2	Snx27	0.11	4	4	3	0	1	1
Cyclin-dependent-like kinase 5 OS=Mus musculus GN=Cdk5 PE=1 SV=1	Cdk5	0.5	6	5	0	2	0	2
Long-chain-fatty-acid--CoA ligase ACSBG1 OS=Mus musculus GN=Acsbg1 PE=1 SV=1	Acsbg1	0.097	2	4	1	0	5	3
Small glutamine-rich tetratricopeptide repeat-containing protein alpha OS=Mus musculus GN=Sgta PE=1 SV=2	Sgta	0.094	3	3	2	2	3	3
Ras-related protein Rab-6B OS=Mus musculus GN=Rab6b PE=1 SV=1	Rab6b	0.64	3	3	1	1	2	2

Serine/threonine-protein phosphatase 2A 56 kDa regulatory subunit epsilon isoform OS=Mus musculus GN=Ppp2r5e PE=1 SV=3	Ppp2r5e	0.77	2	3	3	0	4	3
SH3-containing GRB2-like protein 3-interacting protein 1 OS=Mus musculus GN=Sgip1 PE=1 SV=1	Sgip1	0.66	3	3	0	2	2	3
Mitogen-activated protein kinase OS=Mus musculus GN=Mapk3 PE=1 SV=1	Mapk3	0.057	0	4	4	0	0	0
Misshapen-like kinase 1 OS=Mus musculus GN=Mink1 PE=1 SV=1	Mink1	0.82	3	4	2	2	3	2
Kinesin-1 heavy chain OS=Mus musculus GN=Kif5b PE=1 SV=3	Kif5b	0.91	5	3	0	2	2	1
28 kDa heat- and acid-stable phosphoprotein OS=Mus musculus GN=Pdap1 PE=1 SV=1	Pdap1	0.2	3	1	1	2	6	3
IQ motif and SEC7 domain-containing protein 1 OS=Mus musculus GN=Iqsec1 PE=1 SV=1	Iqsec1	0.073	2	2	0	4	2	2
GTP-binding protein Di-Ras2 OS=Mus musculus GN=Diras2 PE=1 SV=1	Diras2	0.078	0	0	3	3	3	2
Protein transport protein Sec31A OS=Mus musculus GN=Sec31a PE=1 SV=2	Sec31a	0.48	2	2	3	0	2	1
Retinal dehydrogenase 1 OS=Mus musculus GN=Aldh1a1 PE=1 SV=5	Aldh1a1	0.052	2	4	0	0	1	0
Microtubule-associated protein OS=Mus musculus GN=Map4 PE=1 SV=1	Map4	0.22	2	2	0	2	2	4
Protein SGT1 homolog OS=Mus musculus GN=Sugt1 PE=1 SV=3	Sugt1	0.43	2	3	1	0	2	2
Wiskott-Aldrich syndrome protein family member 1 OS=Mus musculus GN=Wasf1 PE=1 SV=2	Wasf1	0.46	4	4	0	2	0	3
Probable ubiquitin carboxyl-terminal hydrolase FAF-X OS=Mus musculus GN=Usp9x PE=1 SV=1	Usp9x	0.098	4	4	1	0	0	2
Early endosome antigen 1 OS=Mus musculus GN=Eea1 PE=1 SV=2	Eea1	0.082	6	8	1	0	1	0
Lysosomal alpha-glucosidase OS=Mus musculus GN=Gaa PE=1 SV=2	Gaa	0.056	1	1	0	5	0	0
Caytaxin OS=Mus musculus GN=Atcay PE=1 SV=1	Atcay	0.071	3	3	2	1	3	3
Neurofilament heavy polypeptide OS=Mus musculus GN=Nefh PE=1 SV=3	Nefh	0.35	1	0	0	4	3	2
Mitogen-activated protein kinase 1 OS=Mus musculus GN=Mapk1 PE=1 SV=3	Mapk1	0.051	2	3	1	0	0	0
Syntaxin 7 OS=Mus musculus GN=Stx7 PE=1 SV=1	Stx7	0.66	2	2	2	1	3	3
Ras-related protein Rab-1B OS=Mus musculus GN=Rab1b PE=1 SV=1	Rab1b	0.82	3	3	2	1	1	1
Hippocalcin-like protein 4 (Fragment) OS=Mus musculus GN=Hpcal4 PE=1 SV=1	Hpcal4	0.83	2	1	1	1	1	1
Neuronal pentraxin-1 OS=Mus musculus GN=Nptx1 PE=1 SV=1	Nptx1	0.094	2	2	0	1	2	2
MAP kinase-activating death domain protein OS=Mus musculus GN=Madd PE=1 SV=1	Madd	0.48	3	3	0	2	4	2
Glutamate decarboxylase 1 OS=Mus musculus GN=Gad1 PE=1 SV=2	Gad1	0.27	1	1	2	1	2	2
Prefoldin subunit 2 OS=Mus musculus GN=Pfdn2 PE=1 SV=2	Pfdn2	0.052	3	3	0	1	1	1
Protein Rap1gds1 OS=Mus musculus GN=Rap1gds1 PE=1 SV=1	Rap1gds1	0.062	1	3	3	0	1	1
Phospholemman OS=Mus musculus GN=Fxyd1 PE=1 SV=1	Fxyd1	0.061	1	1	1	1	1	1
Protein phosphatase 1 regulatory subunit 1A OS=Mus musculus GN=Ppp1r1a PE=1 SV=1	Ppp1r1a	0.084	2	3	0	2	2	2
MCG9889 OS=Mus musculus GN=Gm10709 PE=4 SV=1	Gm10709	0.15	4	4	1	1	1	1

cAMP-regulated phosphoprotein 19 OS=Mus musculus GN=Arpp19 PE=1 SV=2	Arpp19	0.12	3	3	2	0	0	3
Guanylate kinase OS=Mus musculus GN=Guk1 PE=1 SV=1	Guk1	0.063	1	1	2	1	1	1
Isoaspartyl peptidase/L-asparaginase OS=Mus musculus GN=Asrgl1 PE=1 SV=1	Asrgl1	0.21	1	2	4	1	1	1
COP9 signalosome complex subunit 4 OS=Mus musculus GN=Cops4 PE=1 SV=1	Cops4	0.72	2	3	1	1	0	3
MAP7 domain-containing protein 2 OS=Mus musculus GN=Map7d2 PE=1 SV=1	Map7d2	0.42	4	1	0	2	2	2
Profilin OS=Mus musculus GN=Pfn2 PE=1 SV=1	Pfn2	0.87	1	1	1	1	1	1
Disks large homolog 1 OS=Mus musculus GN=Dlg1 PE=1 SV=1	Dlg1	0.11	2	1	0	2	5	2
Protein C10 OS=Mus musculus GN=Grcc10 PE=1 SV=1	Grcc10	0.059	1	2	2	1	2	0
Eukaryotic translation initiation factor 3 subunit H OS=Mus musculus GN=Eif3h PE=1 SV=1	Eif3h	0.058	3	3	2	0	1	1
ADP-ribosylation factor-like protein 3 OS=Mus musculus GN=Arl3 PE=1 SV=1	Arl3	0.084	1	1	2	1	1	0
Synaptosomal-associated protein 47 OS=Mus musculus GN=Snap47 PE=1 SV=1	Snap47	0.36	2	2	2	1	2	0
cAMP-dependent protein kinase inhibitor alpha OS=Mus musculus GN=Pkia PE=1 SV=2	Pkia	0.59	3	2	2	2	0	3
60S ribosomal protein L30 OS=Mus musculus GN=Rpl30 PE=1 SV=2	Rpl30	0.091	2	3	4	0	0	2
V-type proton ATPase subunit a OS=Mus musculus GN=Atp6v0a1 PE=1 SV=1	Atp6v0a1	0.076	2	4	2	1	1	0
Myelin basic protein (Fragment) OS=Mus musculus GN=Mbp PE=1 SV=1	Mbp	0.7	2	2	2	0	0	1
Quinone oxidoreductase OS=Mus musculus GN=Cryz PE=1 SV=1	Cryz	0.25	2	1	2	0	1	3
SUMO-activating enzyme subunit 2 OS=Mus musculus GN=Uba2 PE=1 SV=1	Uba2	0.058	4	3	2	0	0	0
Kinesin light chain 1 OS=Mus musculus GN=Klc1 PE=1 SV=1	Klc1	0.17	2	2	1	2	1	1
Protein SOGA3 OS=Mus musculus GN=Soga3 PE=1 SV=2	Soga3	0.23	2	1	1	5	2	1
Zyx protein OS=Mus musculus GN=Zyx PE=1 SV=1	Zyx	0.15	3	3	0	0	0	1
Dynamin-1 OS=Mus musculus GN=Dnm1 PE=1 SV=2	Dnm1	0.25	0	1	1	0	2	2
Synaptic vesicle membrane protein VAT-1 homolog OS=Mus musculus GN=Vat1 PE=1 SV=3	Vat1	0.2	1	0	1	2	0	3
Ras-related protein Rab-5B OS=Mus musculus GN=Rab5b PE=1 SV=1	Rab5b	0.31	3	0	1	2	1	1
Striatin-4 OS=Mus musculus GN=Strn4 PE=1 SV=2	Strn4	0.33	3	1	2	2	0	1
ARF GTPase-activating protein GIT1 OS=Mus musculus GN=Git1 PE=1 SV=1	Git1	0.44	3	2	0	0	1	2
Copper transport protein ATOX1 OS=Mus musculus GN=Atox1 PE=1 SV=1	Atox1	0.49	2	3	2	0	0	2
Synaptophysin OS=Mus musculus GN=Syp PE=1 SV=2	Syp	0.7	3	3	0	2	1	0
Bifunctional purine biosynthesis protein PURH OS=Mus musculus GN=Atic PE=1 SV=2	Atic	0.056	2	4	6	1	0	0
Dynein light chain 1, cytoplasmic OS=Mus musculus GN=Dynll1 PE=1 SV=1	Dynll1	0.27	0	5	2	0	1	0
Protein-L-isoaspartate O-methyltransferase OS=Mus musculus GN=Pcmt1 PE=1 SV=1	Pcmt1	0.44	0	3	2	0	1	0

Dual specificity mitogen-activated protein kinase kinase 4 OS=Mus musculus GN=Map2k4 PE=1 SV=2	Map2k4	0.33	2	2	2	1	1	1
Glucosidase 2 subunit beta OS=Mus musculus GN=Prkcsh PE=1 SV=1	Prkcsh	0.4	3	3	0	1	1	1
Epidermal growth factor receptor substrate 15-like 1 OS=Mus musculus GN=Eps15l1 PE=1 SV=3	Eps15l1	0.26	0	2	0	2	2	2
Beta-adducin OS=Mus musculus GN=Add2 PE=1 SV=4	Add2	0.73	2	2	1	2	2	2
Neural Wiskott-Aldrich syndrome protein OS=Mus musculus GN=Wasl PE=1 SV=1	Wasl	0.071	1	1	1	1	0	0
Abl interactor 2 OS=Mus musculus GN=Abi2 PE=1 SV=1	Abi2	0.72	0	3	2	2	0	4
Centrosomal protein of 170 kDa protein B OS=Mus musculus GN=Cep170b PE=1 SV=2	Cep170b	0.057	4	4	1	0	0	0
Probable G-protein coupled receptor 158 OS=Mus musculus GN=Gpr158 PE=1 SV=2	Gpr158	0.093	0	0	0	3	4	3
Arf-GAP with GTPase, ANK repeat and PH domain-containing protein 3 OS=Mus musculus GN=Agap3 PE=1 SV=1	Agap3	0.1	2	2	1	2	1	1
3-ketoacyl-CoA thiolase A, peroxisomal OS=Mus musculus GN=Acaa1a PE=1 SV=1	Acaa1a	0.79	2	1	0	1	3	1
Protein kinase C OS=Mus musculus GN=Prkca PE=1 SV=1	Prkca	0.37	1	2	2	1	0	1
Acyl-coenzyme A thioesterase 1 OS=Mus musculus GN=Acot1 PE=1 SV=1	Acot1	0.65	2	2	2	1	1	1
Solute carrier family 2, facilitated glucose transporter member 3 OS=Mus musculus GN=Slc2a3 PE=1 SV=1	Slc2a3	0.18	1	1	0	0	3	2
Density-regulated protein OS=Mus musculus GN=Denr PE=1 SV=1	Denr	0.39	2	0	2	1	0	0
Ras-related protein Rap-1b OS=Mus musculus GN=Rap1b PE=1 SV=2	Rap1b	0.11	2	1	1	0	1	2
Protein NDRG1 OS=Mus musculus GN=Ndrg1 PE=1 SV=1	Ndrg1	0.79	2	2	0	1	1	2
Intersectin-1 OS=Mus musculus GN=Itsn1 PE=1 SV=1	Itsn1	0.7	3	2	0	1	0	1
60S ribosomal protein L27a OS=Mus musculus GN=Rpl27a PE=1 SV=5	Rpl27a	0.094	1	1	2	2	0	1
Staphylococcal nuclease domain-containing protein 1 OS=Mus musculus GN=Snd1 PE=1 SV=1	Snd1	0.12	1	1	4	3	1	3
Rab11 family-interacting protein 5 OS=Mus musculus GN=Rab11fip5 PE=1 SV=1	Rab11fip5	0.16	0	3	0	2	2	1
Low molecular weight phosphotyrosine protein phosphatase OS=Mus musculus GN=Acp1 PE=1 SV=3	Acp1	0.28	3	2	2	0	2	0
Cell adhesion molecule 2 OS=Mus musculus GN=Cadm2 PE=1 SV=2	Cadm2	0.05	0	0	0	4	0	3
Protein TSSC1 OS=Mus musculus GN=Eipr1 PE=1 SV=2	Eipr1	0.2	1	2	3	0	1	1
LanC-like protein 1 (Fragment) OS=Mus musculus GN=Lancl1 PE=1 SV=1	Lancl1	0.7	1	1	1	1	1	0
Calumenin OS=Mus musculus GN=Calu PE=1 SV=1	Calu	0.13	1	0	3	1	0	0
Glycoprotein m6b, isoform CRA_g OS=Mus musculus GN=Gpm6b PE=1 SV=1	Gpm6b	0.14	1	0	0	1	1	3
Regulator of G-protein-signaling 7 OS=Mus musculus GN=Rgs7 PE=1 SV=1	Rgs7	0.14	2	0	0	0	2	2
Hydroxyacylglutathione hydrolase, mitochondrial (Fragment) OS=Mus musculus GN=Hagh PE=1 SV=1	Hagh	0.15	0	2	1	0	0	0
Atlastin-1 OS=Mus musculus GN=Atl1 PE=1 SV=1	Atl1	0.43	2	1	0	2	0	0
Protein NDRG3 OS=Mus musculus GN=Ndrg3 PE=1 SV=1	Ndrg3	0.22	3	3	1	1	0	0

Vacuolar protein sorting-associated protein 29 (Fragment) OS=Mus musculus GN=Vps29 PE=1 SV=1	Vps29	0.28	2	1	0	3	1	0
Probable ATP-dependent RNA helicase DDX6 OS=Mus musculus GN=Ddx6 PE=1 SV=1	Ddx6	0.17	2	4	0	0	0	0
Ras GTPase-activating protein-binding protein 2 OS=Mus musculus GN=G3bp2 PE=1 SV=2	G3bp2	0.15	3	0	3	0	0	0
60S ribosomal protein L17 OS=Mus musculus GN=Rpl17 PE=1 SV=1	Rpl17	0.055	1	2	3	0	0	0
Dystrobrevin alpha OS=Mus musculus GN=Dtna PE=1 SV=2	Dtna	0.13	2	1	1	0	1	1
Complement C1q subcomponent subunit B OS=Mus musculus GN=C1qb PE=1 SV=2	C1qb	0.24	1	1	1	1	0	1
Heterogeneous nuclear ribonucleoprotein Q OS=Mus musculus GN=Syncrip PE=1 SV=1	Syncrip	0.054	2	1	2	1	0	1
Peptidyl-prolyl cis-trans isomerase FKBP1A OS=Mus musculus GN=Fkbp1a PE=1 SV=2	Fkbp1a	0.24	1	2	2	0	0	1
Glycylpeptide N-tetradecanoyltransferase 1 OS=Mus musculus GN=Nmt1 PE=1 SV=1	Nmt1	0.16	2	1	1	0	1	2
Gamma-synuclein OS=Mus musculus GN=Sncg PE=1 SV=1	Sncg	0.57	1	1	0	2	2	0
Hypoxia up-regulated protein 1 OS=Mus musculus GN=Hyou1 PE=1 SV=1	Hyou1	0.074	4	2	2	1	1	1
AMP deaminase 2 OS=Mus musculus GN=Ampd2 PE=1 SV=1	Ampd2	0.1	2	2	2	0	1	0
Peptidyl-prolyl cis-trans isomerase NIMA-interacting 1 OS=Mus musculus GN=Pin1 PE=1 SV=1	Pin1	0.25	1	3	0	0	0	0
Protein phosphatase inhibitor 2 OS=Mus musculus GN=Ppp1r2 PE=1 SV=3	Ppp1r2	0.067	0	0	3	2	0	1
Eukaryotic translation initiation factor 3 subunit A OS=Mus musculus GN=Eif3a PE=1 SV=5	Eif3a	0.11	3	4	0	0	0	0
Nuclear ubiquitous casein and cyclin-dependent kinase substrate 1 OS=Mus musculus GN=Nucks1 PE=1 SV=1	Nucks1	0.1	3	3	0	0	0	0
40S ribosomal protein S5 (Fragment) OS=Mus musculus GN=Rps5 PE=1 SV=1	Rps5	0.29	3	0	3	0	0	0
Membrane-associated phosphatidylinositol transfer protein 1 OS=Mus musculus GN=Pitpnm1 PE=1 SV=1	Pitpnm1	0.72	2	0	0	1	1	1
Cadherin-13 OS=Mus musculus GN=Cdh13 PE=1 SV=2	Cdh13	0.19	1	1	1	2	2	1
DnaJ homolog subfamily A member 2 OS=Mus musculus GN=Dnaja2 PE=1 SV=1	Dnaja2	0.17	1	1	1	1	0	1
COP9 signalosome complex subunit 2 OS=Mus musculus GN=Cops2 PE=1 SV=1	Cops2	0.081	2	1	2	0	0	0
Protein phosphatase methylesterase 1 OS=Mus musculus GN=Ppme1 PE=1 SV=5	Ppme1	0.34	3	3	0	0	0	1
Phosphatidylinositide phosphatase SAC1 OS=Mus musculus GN=Sacm1l PE=1 SV=1	Sacm1l	0.35	2	2	0	0	1	0
26S proteasome non-ATPase regulatory subunit 12 OS=Mus musculus GN=Psmd12 PE=1 SV=4	Psmd12	0.27	2	3	0	0	0	0
SH3 and multiple ankyrin repeat domains protein 2 OS=Mus musculus GN=Shank2 PE=4 SV=1	Shank2	0.064	0	0	0	2	4	0
Nascent polypeptide-associated complex subunit alpha, muscle-specific form OS=Mus musculus GN=Naca PE=1 SV=2	Naca	0.92	1	0	2	1	1	1
Aldose reductase OS=Mus musculus GN=Akr1b1 PE=1 SV=3	Akr1b1	0.72	0	2	0	1	1	2
Chloride intracellular channel protein 1 OS=Mus musculus GN=Clic1 PE=1 SV=3	Clic1	0.11	1	2	3	0	1	0
Connector enhancer of kinase suppressor of ras 2 OS=Mus musculus GN=Cnksr2 PE=1 SV=1	Cnksr2	0.19	2	1	1	1	0	0
Phospholipid phosphatase 3 OS=Mus musculus GN=Plpp3 PE=1 SV=1	Plpp3	0.091	0	0	0	0	1	1

BolA-like protein 2 OS=Mus musculus GN=Bola2 PE=1 SV=1	Bola2	0.28	0	2	4	0	0	1
Protein lin-7 homolog A OS=Mus musculus GN=Lin7a PE=1 SV=2	Lin7a	0.17	0	0	1	2	2	0
MCG129810, isoform CRA_c OS=Mus musculus GN=Pdxdc1 PE=1 SV=1	Pdxdc1	0.052	3	3	0	0	0	0
Protein phosphatase 1G OS=Mus musculus GN=Ppm1g PE=1 SV=3	Ppm1g	0.24	2	2	2	0	0	0
Neuroendocrine protein 7B2 OS=Mus musculus GN=Scg5 PE=1 SV=1	Scg5	0.95	2	2	0	1	1	1
Phosphatidylinositol 5-phosphate 4-kinase type-2 gamma OS=Mus musculus GN=Pip4k2c PE=1 SV=1	Pip4k2c	0.9	1	2	0	2	0	1
Cortactin-binding protein 2 OS=Mus musculus GN=Cttnbp2 PE=1 SV=2	Cttnbp2	0.7	1	1	0	2	1	0
Abl interactor 1 OS=Mus musculus GN=Abi1 PE=1 SV=1	Abi1	0.94	2	0	1	2	0	1
Vigilin OS=Mus musculus GN=Hdlbp PE=1 SV=1	Hdlbp	0.069	1	3	2	0	0	0
Kinesin light chain 2 OS=Mus musculus GN=Klc2 PE=1 SV=1	Klc2	0.35	2	2	1	1	0	0
Eukaryotic translation initiation factor 4 gamma 1 OS=Mus musculus GN=Eif4g1 PE=1 SV=1	Eif4g1	0.077	3	2	1	0	0	0
Protein arginine N-methyltransferase 1 OS=Mus musculus GN=Prmt1 PE=1 SV=1	Prmt1	0.12	0	3	2	0	0	0
Guanine nucleotide-binding protein subunit alpha-13 OS=Mus musculus GN=Gna13 PE=1 SV=1	Gna13	0.57	0	0	0	0	2	0
MARCKS-related protein OS=Mus musculus GN=Marcksl1 PE=1 SV=2	Marcksl1	0.31	1	1	1	0	1	1
Protein A830010M20Rik OS=Mus musculus GN=A830010M20Rik PE=1 SV=1	A830010M20Rik	0.24	2	1	1	0	0	1
Ras-related protein Rab-8B OS=Mus musculus GN=Rab8b PE=1 SV=1	Rab8b	0.16	1	1	0	2	1	1
C-Jun-amino-terminal kinase-interacting protein 3 OS=Mus musculus GN=Mapk8ip3 PE=1 SV=1	Mapk8ip3	0.33	1	1	2	1	1	1
Vesicle-trafficking protein SEC22b OS=Mus musculus GN=Sec22b PE=1 SV=1	Sec22b	0.45	0	2	1	1	1	2
cAMP-dependent protein kinase type I-alpha regulatory subunit OS=Mus musculus GN=Prkar1a PE=1 SV=3	Prkar1a	0.8	1	2	0	1	1	0
Kinectin OS=Mus musculus GN=Ktn1 PE=1 SV=1	Ktn1	0.14	2	1	1	1	1	1
Moesin OS=Mus musculus GN=Msn PE=1 SV=3	Msn	0.11	0	0	0	0	1	2
Type I inositol 3,4-bisphosphate 4-phosphatase OS=Mus musculus GN=Inpp4a PE=1 SV=2	Inpp4a	0.76	0	2	1	0	0	2
Synergin gamma OS=Mus musculus GN=Synrg PE=1 SV=1	Synrg	0.11	2	1	1	0	0	1
Ras-related protein Rab-18 OS=Mus musculus GN=Rab18 PE=1 SV=2	Rab18	0.76	2	0	0	1	0	2
Ubiquitin-40S ribosomal protein S27a OS=Mus musculus GN=Rps27a PE=1 SV=2	Rps27a	0.39	2	0	2	0	0	1
Protein Rpl9-ps6 OS=Mus musculus GN=Rpl9-ps6 PE=4 SV=1	Rpl9-ps6	0.053	1	1	3	0	0	0
NAD(P) transhydrogenase, mitochondrial OS=Mus musculus GN=Nnt PE=1 SV=2	Nnt	0.25	0	0	0	0	0	0
Synaptobrevin homolog YKT6 OS=Mus musculus GN=Ykt6 PE=1 SV=1	Ykt6	0.48	1	1	1	1	1	1
Importin subunit alpha-3 OS=Mus musculus GN=Kpna4 PE=1 SV=1	Kpna4	0.49	1	1	1	0	1	1
Chloride intracellular channel protein 4 OS=Mus musculus GN=Clic4 PE=1 SV=3	Clic4	0.53	1	0	1	0	1	1

TSC22 domain family protein 1 OS=Mus musculus GN=Tsc22d1 PE=1 SV=1	Tsc22d1	0.94	2	1	0	1	1	0
Guanine nucleotide-binding protein subunit gamma OS=Mus musculus GN=Gng7 PE=1 SV=1	Gng7	0.58	1	1	1	0	1	1
Cysteine--tRNA ligase, cytoplasmic OS=Mus musculus GN=Cars PE=1 SV=2	Cars	0.12	1	1	2	0	0	1
Traf2 and NCK-interacting protein kinase OS=Mus musculus GN=Tnik PE=1 SV=1	Tnik	0.23	2	2	1	1	1	0
Protein Ppp1r9a OS=Mus musculus GN=Ppp1r9a PE=1 SV=1	Ppp1r9a	0.43	2	1	0	2	1	1
Protein LYRIC OS=Mus musculus GN=Mtdh PE=1 SV=1	Mtdh	0.081	1	3	1	0	1	0
Cofilin-2 OS=Mus musculus GN=Cfl2 PE=1 SV=1	Cfl2	0.14	1	1	0	0	1	1
Disks large-associated protein 3 OS=Mus musculus GN=Dlgap3 PE=1 SV=1	Dlgap3	0.067	0	1	0	2	1	1
Magi1 protein (Fragment) OS=Mus musculus GN=Magi1 PE=1 SV=1	Magi1	0.78	1	1	0	1	1	0
Unconventional myosin-VI OS=Mus musculus GN=Myo6 PE=1 SV=1	Myo6	0.13	0	2	0	2	0	1
60S ribosomal protein L31 OS=Mus musculus GN=Rpl31 PE=1 SV=1	Rpl31	0.069	3	1	2	1	0	0
Guanine nucleotide-binding protein-like 1 OS=Mus musculus GN=Gnl1 PE=1 SV=4	Gnl1	0.069	1	2	0	0	0	0
Transcription elongation factor B polypeptide 1 (Fragment) OS=Mus musculus GN=Tceb1 PE=1 SV=1	Tceb1	0.09	1	1	2	0	0	0
Prefoldin subunit 1 OS=Mus musculus GN=Pfdn1 PE=1 SV=1	Pfdn1	0.3	0	2	0	0	1	0
Cystatin (Fragment) OS=Mus musculus GN=Cst3 PE=1 SV=1	Cst3	0.074	2	0	2	0	0	0
Histone-lysine N-methyltransferase SETD7 OS=Mus musculus GN=Setd7 PE=1 SV=2	Setd7	0.078	2	1	4	0	0	0
Catechol O-methyltransferase domain-containing protein 1 OS=Mus musculus GN=Comtd1 PE=1 SV=1	Comtd1	0.071	0	0	0	1	1	3
Ethanolamine-phosphate cytidylyltransferase OS=Mus musculus GN=Pcyt2 PE=1 SV=1	Pcyt2	0.17	3	0	4	0	0	0
Sodium/potassium-transporting ATPase subunit beta-2 OS=Mus musculus GN=Atp1b2 PE=1 SV=2	Atp1b2	0.19	0	1	0	2	0	2
Glycerol-3-phosphate phosphatase OS=Mus musculus GN=Pgp PE=1 SV=1	Pgp	0.4	2	0	2	0	0	0
Glyceraldehyde-3-phosphate dehydrogenase OS=Mus musculus GN=Gm3839 PE=1 SV=1	Gm3839	0.13	0	1	0	1	1	1
Spermine synthase OS=Mus musculus GN=Sms PE=1 SV=1	Sms	0.13	2	0	3	0	0	0
Fragile X mental retardation syndrome-related protein 2 OS=Mus musculus GN=Fxr2 PE=1 SV=1	Fxr2	0.18	3	0	2	0	0	0
ADP-ribosylation factor GTPase-activating protein 1 OS=Mus musculus GN=Arfgap1 PE=1 SV=1	Arfgap1	0.28	0	0	0	2	0	0
Synaptotagmin II OS=Mus musculus GN=Syt2 PE=1 SV=1	Syt2	0.36	0	1	0	1	1	1
Vesicle transport through interaction with t-SNAREs 1B homolog OS=Mus musculus GN=Vti1b PE=1 SV=1	Vti1b	0.64	1	1	0	0	1	1
Nucleobindin 1, isoform CRA_b OS=Mus musculus GN=Nucb1 PE=4 SV=1	Nucb1	0.095	1	2	2	0	1	1
BRISC complex subunit Abro1 OS=Mus musculus GN=Fam175b PE=1 SV=1	Fam175b	0.2	2	0	2	0	1	0
Dynein light chain roadblock-type 1 OS=Mus musculus GN=Dynlrb1 PE=1 SV=1	Dynlrb1	0.23	1	2	0	0	0	0
Acyl-coenzyme A thioesterase 11 OS=Mus musculus GN=Acot11 PE=1 SV=1	Acot11	0.2	0	2	1	0	0	1

60S ribosomal protein L24 OS=Mus musculus GN=Rpl24 PE=1 SV=2	Rpl24	0.12	1	1	1	1	0	0
Protein Rpl23a-ps3 OS=Mus musculus GN=Rpl23a-ps3 PE=3 SV=1	Rpl23a-ps3	0.15	1	2	1	0	1	1
Rab-like protein 6 OS=Mus musculus GN=Rabl6 PE=1 SV=2	Rabl6	0.73	2	0	0	0	2	1
Rho-related GTP-binding protein RhoG OS=Mus musculus GN=Rhog PE=1 SV=1	Rhog	0.43	2	2	1	0	2	0
Eukaryotic translation initiation factor 2 subunit 1 OS=Mus musculus GN=Eif2s1 PE=1 SV=3	Eif2s1	0.21	1	1	2	0	0	0
UBX domain-containing protein 6 OS=Mus musculus GN=Ubxn6 PE=1 SV=1	Ubxn6	0.43	1	0	4	0	0	0
Formin-2 OS=Mus musculus GN=Fmn2 PE=1 SV=2	Fmn2	0.15	0	1	0	0	3	1
Multifunctional protein ADE2 OS=Mus musculus GN=Paics PE=1 SV=4	Paics	0.49	2	2	0	0	0	2
Rap1 GTPase-activating protein 2 OS=Mus musculus GN=Rap1gap2 PE=1 SV=1	Rap1gap2	0.56	2	2	0	0	1	2
Thioredoxin-related transmembrane protein 4 OS=Mus musculus GN=Tmx4 PE=1 SV=2	Tmx4	0.53	3	0	1	2	0	0
Alpha-actinin-4 OS=Mus musculus GN=Actn4 PE=1 SV=1	Actn4	0.093	0	0	0	0	1	2
MCG7614, isoform CRA_c OS=Mus musculus GN=Srsf5 PE=1 SV=1	Srsf5	0.08	1	0	3	0	0	0
V-type proton ATPase subunit d 1 OS=Mus musculus GN=Atp6v0d1 PE=1 SV=2	Atp6v0d1	0.11	0	3	1	0	0	0
Beta-actin-like protein 2 OS=Mus musculus GN=Actbl2 PE=1 SV=1	Actbl2	0.7	1	0	0	0	0	1
Secretory carrier-associated membrane protein 5 OS=Mus musculus GN=Scamp5 PE=1 SV=1	Scamp5	0.6	0	1	0	0	0	2
Ubiquitin carboxyl-terminal hydrolase isozyme L3 OS=Mus musculus GN=Uchl3 PE=1 SV=2	Uchl3	0.15	2	0	2	0	0	0
Phosphofurin acidic cluster sorting protein 1 OS=Mus musculus GN=Pacs1 PE=1 SV=2	Pacs1	0.52	0	0	0	2	0	2
Eukaryotic translation initiation factor 3 subunit G OS=Mus musculus GN=Eif3g PE=1 SV=2	Eif3g	0.11	0	3	2	0	0	0
Thioredoxin domain-containing protein 17 OS=Mus musculus GN=Txndc17 PE=1 SV=1	Txndc17	0.25	1	0	4	0	0	0
F-box/LRR-repeat protein 16 OS=Mus musculus GN=Fbxl16 PE=1 SV=1	Fbxl16	0.21	0	2	1	0	0	0
Cytosol aminopeptidase OS=Mus musculus GN=Lap3 PE=1 SV=3	Lap3	0.44	0	0	3	3	0	2
Transmembrane protein 163 OS=Mus musculus GN=Tmem163 PE=1 SV=1	Tmem163	0.14	0	0	0	2	2	0
Rap1 GTPase-activating protein 1 OS=Mus musculus GN=Rap1gap PE=1 SV=2	Rap1gap	0.53	0	4	0	0	0	1
Cell cycle control protein 50A OS=Mus musculus GN=Tmem30a PE=1 SV=1	Tmem30a	0.78	0	1	0	0	0	0
Hydroxymethylglutaryl-CoA synthase, cytoplasmic OS=Mus musculus GN=Hmgcs1 PE=1 SV=1	Hmgcs1	0.28	0	0	1	0	0	0
Maleylacetoacetate isomerase OS=Mus musculus GN=Gstz1 PE=1 SV=1	Gstz1	0.07	0	0	0	0	0	0
WW domain binding protein 2, isoform CRA_a OS=Mus musculus GN=Wbp2 PE=1 SV=1	Wbp2	0.6	0	0	2	0	0	0
Inactive phospholipase C-like protein 2 OS=Mus musculus GN=Plcl2 PE=1 SV=2	Plcl2	0.24	1	1	1	1	0	0
Disks large-associated protein 1 OS=Mus musculus GN=Dlgap1 PE=1 SV=1	Dlgap1	0.21	1	1	0	0	0	1
Target of Myb protein 1 OS=Mus musculus GN=Tom1 PE=1 SV=1	Tom1	0.32	1	1	1	1	0	1

Growth arrest-specific protein 7 OS=Mus musculus GN=Gas7 PE=1 SV=1	Gas7	0.38	0	2	0	2	1	1
Receptor expression-enhancing protein OS=Mus musculus GN=Reep5 PE=1 SV=1	Reep5	0.19	2	0	1	1	1	0
Protein NDRG4 OS=Mus musculus GN=Ndrg4 PE=1 SV=1	Ndrg4	0.17	1	1	1	1	1	0
PEX5-related protein OS=Mus musculus GN=Pex5l PE=1 SV=1	Pex5l	0.15	1	1	0	0	0	0
WD repeat-containing protein 37 OS=Mus musculus GN=Wdr37 PE=1 SV=1	Wdr37	0.16	0	0	0	1	1	1
Amyloid beta A4 precursor protein-binding family A member 1 OS=Mus musculus GN=Apba1 PE=1 SV=2	Apba1	0.059	1	1	1	0	1	0
Cytochrome b5 OS=Mus musculus GN=Cyb5a PE=1 SV=2	Cyb5a	0.054	0	0	0	1	1	2
Calcyclin-binding protein OS=Mus musculus GN=Cacybp PE=1 SV=1	Cacybp	0.23	3	2	0	0	0	0
Protein Ppp2r5d OS=Mus musculus GN=Ppp2r5d PE=1 SV=1	Ppp2r5d	0.65	1	2	0	0	0	0
Serrate RNA effector molecule homolog OS=Mus musculus GN=Srrt PE=1 SV=1	Srrt	0.13	1	3	0	0	0	0
Metallothionein-3 OS=Mus musculus GN=Mt3 PE=1 SV=1	Mt3	0.14	0	0	0	3	0	2
Heterogeneous nuclear ribonucleoprotein D-like OS=Mus musculus GN=Hnrnpdl PE=1 SV=1	Hnrnpdl	0.07	0	1	2	0	0	0
Partner of Y14 and mago OS=Mus musculus GN=Pym1 PE=1 SV=2	Pym1	0.059	3	1	0	0	0	0
Craniofacial development protein 1 OS=Mus musculus GN=Cfdp1 PE=1 SV=1	Cfdp1	0.092	2	0	1	0	0	0
Ran-binding protein 3 OS=Mus musculus GN=Ranbp3 PE=1 SV=2	Ranbp3	0.14	2	0	3	0	0	0
Protein 2310035C23Rik OS=Mus musculus GN=2310035C23Rik PE=1 SV=1	2310035C23Rik	0.13	3	2	0	0	0	0
Metabotropic glutamate receptor 5 OS=Mus musculus GN=Grm5 PE=1 SV=1	Grm5	0.13	0	0	0	0	2	2
Apolipoprotein A-IV OS=Mus musculus GN=Apoa4 PE=1 SV=3	Apoa4	0.087	0	3	3	0	0	0
Epsin-2 OS=Mus musculus GN=Epn2 PE=1 SV=1	Epn2	0.22	2	3	0	0	0	0
Phosphatase and actin regulator 1 OS=Mus musculus GN=Phactr1 PE=1 SV=1	Phactr1	0.12	0	1	0	1	0	0
Transcription factor BTF3 homolog 4 OS=Mus musculus GN=Btf3l4 PE=1 SV=1	Btf3l4	0.3	3	0	2	0	0	0
Rho GDP-dissociation inhibitor 2 (Fragment) OS=Mus musculus GN=Arhgdib PE=1 SV=1	Arhgdib	0.23	2	0	2	0	0	0
NFU1 iron-sulfur cluster scaffold homolog, mitochondrial OS=Mus musculus GN=Nfu1 PE=1 SV=1	Nfu1	0.096	0	0	0	0	0	1
Tyrosine-protein kinase OS=Mus musculus GN=Matk PE=1 SV=1	Matk	1	1	1	0	0	1	1
Charged multivesicular body protein 5 OS=Mus musculus GN=Chmp5 PE=1 SV=1	Chmp5	0.21	1	1	1	0	0	0
Serine/threonine-protein kinase DCLK2 OS=Mus musculus GN=Dclk2 PE=1 SV=1	Dclk2	0.68	1	1	0	1	2	0
Tumor protein D54 OS=Mus musculus GN=Tpd52l2 PE=1 SV=1	Tpd52l2	0.66	0	0	1	2	1	0
60S ribosomal protein L13a (Fragment) OS=Mus musculus GN=Rpl13a PE=4 SV=1	Rpl13a	0.66	0	1	1	1	0	1
MAGUK p55 subfamily member 6 OS=Mus musculus GN=Mpp6 PE=1 SV=1	Mpp6	0.1	1	1	0	1	0	0
